# Coordinated alternative splicing decisions via stepwise exon definition

**DOI:** 10.1093/nar/gkag464

**Published:** 2026-05-19

**Authors:** Panajot Kristofori, Zijie Xiao, Simon Braun, Anke Busch, Kathi Zarnack, Julian König, Stefan Legewie, Congxin Li

**Affiliations:** Department of Systems Biology, Institute for Biomedical Genetics (IBMG), University of Stuttgart, 70569 Stuttgart, Germany; Stuttgart Research Center for Systems Biology (SRCSB), University of Stuttgart, 70569 Stuttgart, Germany; Department of Systems Biology, Institute for Biomedical Genetics (IBMG), University of Stuttgart, 70569 Stuttgart, Germany; Stuttgart Research Center for Systems Biology (SRCSB), University of Stuttgart, 70569 Stuttgart, Germany; Institute of Molecular Biology (IMB), Ackermannweg 4, 55128 Mainz, Germany; Institute of Molecular Biology (IMB), Ackermannweg 4, 55128 Mainz, Germany; Buchmann Institute for Molecular Life Sciences (BMLS), Goethe University Frankfurt, Max-von-Laue-Str. 15, 60438 Frankfurt, Germany; Theodor Boveri Institute, University Würzburg, Biocenter, Am Hubland, 97074 Würzburg, Germany; Institute of Molecular Biology (IMB), Ackermannweg 4, 55128 Mainz, Germany; Theodor Boveri Institute, University Würzburg, Biocenter, Am Hubland, 97074 Würzburg, Germany; Department of Systems Biology, Institute for Biomedical Genetics (IBMG), University of Stuttgart, 70569 Stuttgart, Germany; Stuttgart Research Center for Systems Biology (SRCSB), University of Stuttgart, 70569 Stuttgart, Germany; Department of Systems Biology, Institute for Biomedical Genetics (IBMG), University of Stuttgart, 70569 Stuttgart, Germany; Stuttgart Research Center for Systems Biology (SRCSB), University of Stuttgart, 70569 Stuttgart, Germany

## Abstract

Alternative splicing of pre-mRNA is a fundamental step in human gene regulation, and aberrant splicing is frequently linked to complex diseases, including cancer. All splicing events, such as skipping or inclusion of an alternative exon (AE) and intron retention (IR), are regulated by a common molecular machinery, the spliceosome. However, it remains elusive how the spliceosome coordinates different splicing decisions. Here, we analyzed a large-scale mutagenesis screen and transcriptome-wide RNA sequencing data to show that intron retention adjacent to alternative exons most commonly occurs at intermediate inclusion levels. Using data-driven mathematical modeling, we revealed that multistep exon recognition mediated by spliceosome assembly and maturation explains the observed AE-IR dependency for *cis-*acting sequence mutations and upon knockdown of *trans-*acting RNA-binding proteins. Furthermore, we found that multistep exon recognition is commonly perturbed in a transcriptome-wide manner in cancer cells, which leads to the coordinated deregulation of IR and AE decisions. In total, our work suggests that stepwise alternative exon recognition by the spliceosome coordinates AE inclusion and the retention of flanking introns, which may facilitate the search for common molecular mechanisms for mis-splicing in cancer.

## Graphical Abstract

**Graphical Abstract:**
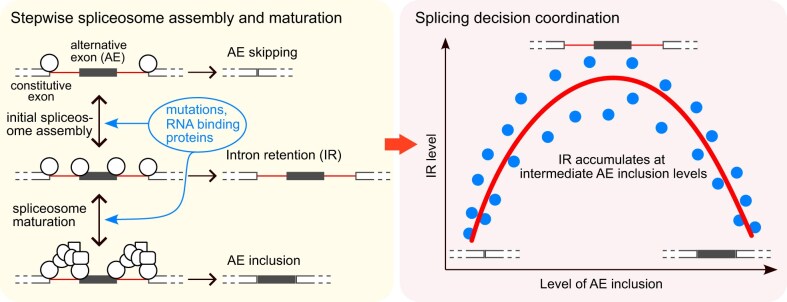

## Introduction

Alternative splicing (AS) of pre-mRNA amplifies the diversity of the human transcriptome and proteome, allowing for robust and versatile gene expression in normal physiology and development. Over 90% of the gene transcripts are alternatively spliced, yielding an average of 3.4 isoforms per gene, and in some cases, thousands of isoforms can emerge [1–3]. The diverse isoforms are produced via a relatively small number of modular AS events, such as the inclusion/exclusion of an alternative exon (AE), intron retention (IR), and alternative use of 5′ or 3′ splice sites (SSs). Among these AS events, IR has long been overlooked in mammalian systems. IR is generally thought of as a mis-splicing incidence triggering the clearance of the IR isoforms via nonsense-mediated mRNA decay (NMD). Recent experimental and computational advances have, however, revealed that IR is essential to regulate many biological functions, e.g. facilitating prompt cell response to stress [4, 5] as well as coordinating gene expression programs in cell differentiation [6, 7]. At the transcriptome-wide level, IR was found to be more prevalent than previously thought, and it is the most affected AS event in most cancer types [8–10]. Thus, there is a pressing need to understand the regulatory rules of splicing decisions involving IR.

Splicing is carried out by the spliceosome, a megadalton ribonucleoprotein complex that progressively assembles on a pre-mRNA and undergoes a series of remodeling steps. Initially, spliceosomal U1 and U2 small nuclear ribonucleoproteins (snRNPs) bind to the 5′SS and the branch point (BP) upstream to the 3′SS, respectively. Further recruitment of the U4/U6.U5 tri-snRNP leads to the formation of a cross-intron complex (B complex), establishing 5′ - 3′SS communication. The B complex then undergoes a series of energy-dependent remodeling steps and becomes active for subsequent intron-removal catalysis. The splicing decision determined by this cross-intron spliceosome assembly is referred to as intron definition [11]. Another spliceosome assembly path is mediated by the formation of a cross-exon complex, termed exon definition. Via extensive ATP-dependent remodeling and structural rearrangements, the cross-exon B-like complex converts to a cross-intron complex for active splicing catalysis [12–16]. The exon definition mechanism is preferentially used in human cells, where most transcripts have short exons and long introns. A common feature of both working modes is the ordered stepwise maturation of the spliceosome driven by energy expenditure, implying the existence of multiple rate-limiting steps. Interestingly, depletion of the spliceosome components and regulators involved in early splice-site recognition steps (e.g. U1 and U2 factors) mainly affects AE usage, whereas perturbations in later steps, such as splice-site communication and spliceosome remodeling, preferentially change IR levels [10]. This suggests that modulating different steps of spliceosome maturation yields different AS outcomes.

Splicing decisions are tightly controlled by *cis-* and *trans-*acting regulators. A compendium of sequence motifs, to which core spliceosome components and specific RNA-binding proteins (RBPs) are recruited, has been identified as enhancers or silencers for various splicing events. Very often, these elements function in a combinatorial and context-dependent manner, posing a challenge to quantitatively predict their effects on the splicing outcomes. Computational algorithms are being actively developed to integrate these sequence features into predictive models employing a wide range of machine learning techniques and deep-learning neural network architectures [17]. Trained by ever-accumulating genomic sequencing data, these models are optimized to predict the splicing decisions directly from primary sequences [18–23]. However, these models mainly focus on binary decisions, e.g. included vs. skipped AE, removed vs. retained intron, and therefore do not describe complex splicing outcomes involving more than two isoforms.

In fact, over one third of the splicing events in humans show complex splicing patterns combining multiple AS decisions at the same splice junction [24, 25]. Large-scale mutagenesis experiments showed that even a three-exon minigene can produce multiple isoforms by combining different splicing decisions already in wildtype (WT) conditions and that mutations may activate cryptic splice sites, further enriching the splicing choices [26–28]. Thus, a single metric, such as PSI (percent spliced-in), describing the inclusion level of an AE, is not sufficient to quantify the complex splicing decisions. To address this issue, kinetic models have been developed to describe the generation of multiple isoforms from the same transcript combining several splicing events [26, 27, 29–31]. These models allow for analyzing the regulation of multiple splicing outcomes. However, a quantitative description of how IR coordinates with other splicing decisions is still missing.

In this work, we show that IR responds in a non-monotonic way when AE recognition is regulated by mutations: IR accumulates most strongly if an AE flanking the retained introns shows intermediate inclusion levels. This regulatory dependency is observed in a minigene for *RON* (*MST1R*) exon 11 subjected to large-scale mutagenesis as well as in transcriptome-wide data from over 20 human tissues. We propose a mechanistic model driven by the *RON* mutagenesis data and find that the multistep AE definition underlies the coordinated AE-IR regulation induced by *cis-*acting mutations and depletion of *trans-*acting RBPs. At the transcriptome-wide level, the multistep AE definition model accounts for IR accumulation observed in many cancer types and explains its coordinated regulation with nearby AE events. Taken together, we provide evidence of coordination between two important splicing events in response to perturbations, including those occurring in cancer compared to normal cells.

## Materials and methods

### Analyzing read coverage *RON* minigene sequencing data

To test whether the observed PSI-efficiency dependence is robust against read coverage filtering during RNA-seq quantification, we performed analysis on RNA-seq data from the high-throughput *RON* mutagenesis screen using different coverage cutoffs. The point mutation data that we used for model calibration in this study were obtained by linear regression analysis on the RNA-seq quantification of *RON* minigenes with randomly combined mutations (on average 3.6 point mutations per minigene) [26]. To find out whether read coverage affects the observed efficiency drop at intermediate PSIs, we reanalyzed the RNA-seq data of *RON* minigenes with combined mutations using different coverage cutoffs. The RNA-seq data were already filtered to keep minigenes with more than 100 reads [26]. To study the AE-specific mutation effects, we selected minigenes harboring mutations only in the AE but not in the outer constitutive exons (1020 minigenes). We indeed observed a pronounced splicing efficiency drop at intermediate PSIs (Supplementary Fig. S1B). To test even larger coverage cutoffs, we used 200, 400, 800, and 1600 reads per minigene and calculated the PSI-efficiency relation. As expected, the PSI-efficiency dependency is robust against read coverage filtering, suggesting that the coordination of AE-IR decisions is a solid observation in the *RON* minigene.

### Detecting PSI-efficiency dependency in *CD19* minigene

We analyzed the PSI-efficiency relationship in our additional mutagenesis dataset of a minigene that consists of *CD19* exon 1 – 3 [27]. Using similar approaches as for *RON*, this work combined high-throughput mutagenesis assay and data-driven modeling to quantify the single mutation effects on *CD19* splicing and thus CART-19 therapy resistance. Five major isoforms were detected in both WT and mutants: exon 2 inclusion and skipping, second intron retention, as well as two inclusion isoform variants using alternative 3′SS on exon 2 and 3. For mutations in/near exon 2 (the AE; -128 nt upstream of the 3′SS and + 148 nt downstream of the 5′SS of exon 2), splicing efficiency indeed drops at intermediate PSIs, but this decline is not as strong as that observed in *RON* (Supplementary Fig. S2). Yet this weak AE-IR coordination can still be captured quantitatively by our two-step AE definition model. Thus, the analysis on the CD19 dataset supports our conclusion of AE-IR dependency and suggests that the dependency strength may vary among genes.

### Analysis of transcriptome-wide RBP KD data by Rogalska *et al*.

In the genome-wide data from Rogalska *et al*. [10], the effect of RBP KDs was analyzed by genome-wide RNA sequencing, yielding quantifications for ∼84.000 binary splicing events in 300 KD conditions. To analyze the PSI-efficiency relation, we extracted all exon skipping events associated with immediately upstream or downstream intron retention events, yielding 12 252 and 12 260 linked quantifications, respectively. Changes in exon skipping (PSI) were related to changes in splicing efficiency, calculating the latter as 1-PIR, where PIR is the percent intron retention junction reads divided by all upstream or downstream junction reads.

When we analyzed the PSI-efficiency relationship on a global scale, i.e. across all conditions and all linked splicing events, we indeed observed that a substantial drop in splicing efficiency below 95% was indeed most commonly observed nearby AEs with intermediate PSI values (Supplementary Fig. S3A), as expected. Furthermore, RBP knockdowns not only regulated PSI and splicing efficiency individually, but caused coordinated changes in both outcomes: to show this, we related the changes in exon skipping (dPSI) and splicing efficiency (dSE) across all RBP knockdowns, and found that substantial efficiency changes (dSE > 5%) were most common for intermediate dPSI bins (Supplementary Fig. S3B). For instance, in the dPSI interval [-30%;-40%], around 15% of the events showed accompanying efficiency changes > 5%. Thus, the coordinated regulation of PSI and splicing efficiency we report in our paper is commonly observed in the Rogalska dataset, even though individual regulation of SE and PSI seems to be the more common outcome of RBP perturbations.

To further analyze coordinated PSI-efficiency changes at the individual event level, we focused on a subset of 34 and 27 events with exon skipping events with accompanying intron retention showing: (i) High sensitivity to RBP KDs, quantified as a PSI standard deviation > 10% across all conditions, ensuring that a sufficiently large PSI range is covered in the data. (ii) Non-zero intron retention is measured in more than 100 knockdown conditions to ensure that splicing efficiency is reliably measured and not dominated by dropouts in which no corresponding RNA could be measured. For these events, we individually plotted the PSI-efficiency relationship as efficiency box plots across PSI bins (Supplementary Fig. S3C and D). In support of our claims in the paper, we frequently observe a pronounced efficiency drop at intermediate PSI levels, although this behavior is not observed for all events. Taken together, the Rogalska dataset supports that the proposed PSI-efficiency relationship can occur at the global and individual event levels.

### Analysis of transcriptome-wide data from MAJIQlopedia

To investigate the Efficiency-PSI dependency, we utilized publicly available datasets from MAJIQlopedia [32]. Data for healthy tissues were obtained from GTEx [33], BLUEPRINT [34], Leucegene [35], and [36–39]. Cancer tissue datasets were collected from TCGA [40], TARGET [41], BEAT-AML [42], and CBTN [43].

Data for healthy and cancer tissues were collected in two separate TSV tables, respectively. Each table contains the identity of local splice variation (LSV) events (IR or non-IR), junction coordinates, indication of intron retention occurrence, and frequencies of LSV events. The data were filtered by the following criteria. First, only LSV with 3 events were considered, with at least one being IR. This requirement for multiple splicing events to be detected likely biases the analysis towards genes with high overall expression levels. In contrast, since the quantification of splicing events is based on junction reads (exon-exon junctions for productive splicing and exon-intron junctions for unproductive splicing), an intron-length-dependent bias is not expected. Second, to exclude events of using alternative 3′ or/and 5′ splice sites, we selected only events where the absolute distance between the two non-IR events exceeded 300 nucleotides (twice the average human exon length). Third, to ensure robust quantification, we removed LSVs where the sum of the lowest two events was less than 1%.

The three events were classified as inclusion (short junction), skipping (long junction), and IR (event denoted as IR). To ensure comparability between datasets, we retained only the common LSVs in both healthy and cancer tissue tables. For this dataset, PSI was calculated as $PSI = \frac{{\textit{inclusion}}}{{\textit{inclusion} + \textit{skipping}}} \cdot 100{\mathrm{\% }}$, and efficiency was calculated as the sum of inclusion and skipping. To obtain the average PSI and efficiency for normal and cancer tissues, we lumped multiple cell types belonging to the same tissue (Supplementary Table S1) and calculated their corresponding mean values.

The original MAJIQlopedia datasets contain only LSVs supported by at least 10 sequencing reads [32]. With the exact sequencing read counts for individual junctions kindly provided from the MAJIQlopedia authors, i.e. the bootstrapped median and mean counts of all detected junction reads (together with quantile counts), we re-quantified the PSI-efficiency relation employing multiple and more stringent filtering criteria on read coverage for normal and cancer tissues, keeping AE-IR LSVs with total junction reads above 10, 20, 50, 80, and 100. The analysis showed that the trend of PSI-efficiency dependency, an efficiency drop at intermediate PSIs, is consistent across different read coverage filtering stringencies (Supplementary Fig. S4A and B). To characterize the AE-IR coordination in quantitative terms, we calculated the bootstrapped difference between median efficiency at intermediate PSIs (40–60%) and that at extreme PSIs (combined 0–10% and 90–100% PSI bins) for these distinctly filtered PSI-efficiency data (Supplementary Fig. S4C). In all normal tissues and most cancer tissues (17 out of 20), the efficiency at intermediate PSIs declines significantly (one-sided Wilcoxon signed-rank test at 95% confidence level), independent of the read coverage used for filtering. For the remaining 3 cancer tissues, the efficiency decline was not always significant across five coverage cutoffs, but the size of efficiency change was consistently close to zero in all cases, regardless of statistical significance, indicating that the PSI-efficiency dependency in these three cancer tissues is inherently weak. These analyses suggest that the PSI-efficiency dependency is not a result of quantification bias, but a robust biological phenomenon. In addition, to test whether the difference between healthy and cancer tissues arises from distinct read coverage, we compared overall coverage per LSV between the two conditions and found that they are very similar to each other, with a median count of around 50 reads (Supplementary Fig. S4D). Thus, the global alteration of the AE-IR coordination in cancer is a robust phenomenon not influenced by RNA sequencing read coverage.

### Mathematical modeling of multiple splicing decisions of *RON* minigene

The pre-mRNAs of WT *RON* minigene (containing exons 10–12) give rise to five splice isoforms: AE inclusion, AE skipping, first, second, and full intron retention (Supplementary Fig. S5). Mutations and RBP KDs (siRNAs listed in Supplementary Table S2) shift the distribution of the five isoforms, where IR accumulates preferentially at intermediate AE inclusion level. To quantitatively explain this coordinated regulation of AE-IR decisions, we studied and compared two models based on our previous work [31], namely one-step and two-step AE definition models, which will be described in detail in the following sections.

### One-step AE definition model

Splicing is carried out by the spliceosome, a highly complex megadalton RNA-protein complex. The human spliceosome frequently operates via a so-called exon definition mechanism. An exon is defined when the early spliceosomal U1 and U2 snRNPs bind to the 5′SS and branch point near the 3′SS, respectively, and form a cross-exon complex. Then the cross-exon spliceosome assembly undergoes a transition to a cross-intron complex and becomes active for splicing catalysis [15, 16]. In the one-step model, we assume that the initial U1 and U2 binding plays a decisive role in splicing decisions and thus the binding patterns determine the splicing outcomes.

In the model, the pre-mRNA is synthesized at a constant rate (*s*), denoted as $P0\_0\_0$ (Supplementary Fig. S5A), where the underline ‘_’ indicates the presence of an intron (unspliced). The spliceosome binds independently to exons 1–3 with association rates ${{k}_1}$, ${{k}_2}$, and ${{k}_3}$, respectively, leading to a total of eight spliceosomal binding states, or exon definition states. All binding reactions are assumed to be reversible, with dissociation rates ${{k}_4}$, ${{k}_5}$, and ${{k}_6}$ for exons 1–3, respectively. Depending on the exon definition states, splicing decisions are made and carried out by irreversible splicing catalysis with rate ${{k}_{{\mathrm{spli}}}}$. Following the exon definition mechanism, a splicing event occurs when an intron or splice junction is flanked by two fully defined exons. For instance, skipping of exon 2 is produced from pre-mRNAs with two outer exons defined and AE undefined, i.e. the $P1\_0\_1$ state. Likewise, removal of the first intron occurs from the species $P1\_1\_0$ and $P1\_1\_1$, resulting in two intermediate states $P11\_0$ and $P11\_1$. If these two intermediate states were exported from the nucleus, they would form the intron 2 retention isoform. Alternatively, they can proceed with further splicing reactions within the nucleus. Upon exon 3 definition, the $P11\_0$ state becomes $P11\_1$ state that gives rise to the AE inclusion isoform after intron 2 removal. Similarly, the AE inclusion and intron 1 retention isoforms are produced via a symmetric pathway starting with the removal of intron 2 mediated by $P0\_11$ and $P1\_11$ states.

We assume that the transcripts with both introns removed ($P111$ and $P101$ states) are exported from the nucleus very rapidly, with no delay after splicing. Those with at least one intron remained (eight states without intron removal and four intermediate states with one intron spliced out) are either further spliced or slowly exported from the nucleus with rate ${{k}_{{\mathrm{ret}}}}$. Therefore, full and partial intron retention isoforms are the outcomes of competition between the splicing reaction and nuclear export. After being exported to the cytoplasm, the splicing products are subject to degradation. We used isoform-specific degradation rates (${{k}_{{\mathrm{incl}}}}$, ${{k}_{{\mathrm{skip}}}}$, ${{k}_{{\mathrm{dr1}}}}$ and ${{k}_{{\mathrm{dr2}}}}$ for AE inclusion, skipping, first and second intron retention, respectively). The degradation rate of the full intron retention isoform is the sum of ${{k}_{{\mathrm{dr1}}}}$ and ${{k}_{{\mathrm{dr2}}}}$.

We translated the one-step model into the following ordinary differential equations (ODEs):


\begin{eqnarray*}
\begin{array}{@{}*{1}{l}@{}} {\displaystyle\frac{{dP0\_0\_0}}{{dt}} = {{k}_4} \cdot P1\_0\_0 + {{k}_5} \cdot P0\_1\_0 + {{k}_6} \cdot P0\_0\_1} { + s - \left( {{{k}_1} + {{k}_2} + {{k}_3} + {{k}_{{\mathrm{ret}}}}} \right) \cdot P0\_0\_0}\\{\displaystyle\frac{{dP1\_0\_0}}{{dt}} = {{k}_1} \cdot P0\_0\_0 + {{k}_5} \cdot P1\_1\_0 + {{k}_6} \cdot P1\_0\_1}{ - \left( {{{k}_2} + {{k}_3} + {{k}_4} + {{k}_{{\mathrm{ret}}}}} \right) \cdot P1\_0\_0}\\ {\displaystyle\frac{{dP0\_1\_0}}{{dt}} = {{k}_2} \cdot P0\_0\_0 + {{k}_4} \cdot P1\_1\_0 + {{k}_6} \cdot P0\_1\_1} {- \left( {{{k}_1} + {{k}_3} + {{k}_5} + {{k}_{{\mathrm{ret}}}}} \right) \cdot P0\_1\_0}\\ {\displaystyle\frac{{dP0\_0\_1}}{{dt}} = {{k}_3} \cdot P0\_0\_0 + {{k}_4} \cdot P1\_0\_1 + {{k}_5} \cdot P0\_1\_1} {- \left( {{{k}_1} + {{k}_2} + {{k}_6} + {{k}_{{\mathrm{ret}}}}} \right) \cdot P0\_0\_1}\\ {\displaystyle\frac{{dP1\_1\_0}}{{dt}} = {{k}_1} \cdot P0\_1\_0 + {{k}_2} \cdot P1\_0\_0 + {{k}_6} \cdot P1\_1\_1} { - \left( {{{k}_3} + {{k}_4} + {{k}_5} + {{k}_{{\mathrm{ret}}}} + {{k}_{{\mathrm{spli}}}}} \right) \cdot P1\_1\_0}\\ {\displaystyle\frac{{dP1\_0\_1}}{{dt}} = {{k}_1} \cdot P0\_0\_1 + {{k}_3} \cdot P1\_0\_0 + {{k}_5} \cdot P1\_1\_1} { - \left( {{{k}_2} + {{k}_4} + {{k}_6} + {{k}_{{\mathrm{ret}}}} + {{k}_{{\mathrm{spli}}}}} \right) \cdot P1\_0\_1}\\ {\displaystyle\frac{{dP0\_1\_1}}{{dt}} = {{k}_2} \cdot P0\_0\_1 + {{k}_3} \cdot P0\_1\_0 + {{k}_4} \cdot P1\_1\_1} { - \left( {{{k}_1} + {{k}_6} + {{k}_5} + {{k}_{{\mathrm{ret}}}} + {{k}_{{\mathrm{spli}}}}} \right) \cdot P0\_1\_1}\\{\displaystyle\frac{{dP1\_1\_1}}{{dt}} = {{k}_1} \cdot P0\_1\_1 + {{k}_2} \cdot P1\_0\_1 + {{k}_3} \cdot P1\_1\_0} { - \left( {{{k}_4} + {{k}_5} + {{k}_6} + {{k}_{{\mathrm{ret}}}} + 2{{k}_{{\mathrm{spli}}}}} \right) \cdot P1\_1\_1} \end{array}
\end{eqnarray*}

(1) \begin{eqnarray*}
\begin{array}{@{}*{1}{l}@{}} {\displaystyle\frac{{dP11\_0}}{{dt}} = {{k}_{{\mathrm{spli}}}} \cdot P1\_1\_0 + {{k}_4} \cdot P1\_1\_1} { - \left( {{{k}_1} + {{k}_{{\mathrm{ret}}}}} \right) \cdot P11\_0}\\ {\displaystyle\frac{{dP11\_1}}{{dt}} = {{k}_1} \cdot P1\_1\_0 + {{k}_{{\mathrm{spli}}}} \cdot P1\_1\_1} {- \left( {{{k}_4} + {{k}_{{\mathrm{spli}}}} + {{k}_{{\mathrm{ret}}}}} \right) \cdot P11\_1}\\ {\displaystyle\frac{{dP0\_11}}{{dt}} = {{k}_{{\mathrm{spli}}}} \cdot P0\_1\_1 + {{k}_6} \cdot P1\_1\_1} { - \left( {{{k}_3} + {{k}_{{\mathrm{ret}}}}} \right) \cdot P0\_11}\\ {\displaystyle\frac{{dP1\_11}}{{dt}} = {{k}_3} \cdot P0\_1\_1 + {{k}_{{\mathrm{spli}}}} \cdot P1\_1\_1} { - \left( {{{k}_6} + {{k}_{{\mathrm{spli}}}} + {{k}_{{\mathrm{ret}}}}} \right) \cdot P1\_11}\\ {\displaystyle\frac{{d{\mathrm{inclusion}}}}{{dt}} = {{k}_{{\mathrm{spli}}}} \cdot \left( {P1\_11 + P11\_1} \right) - {{k}_{{\mathrm{incl}}}} \cdot {\mathrm{inclusion}}}\\ {\displaystyle\frac{{d\mathrm{skipping}}}{{dt}} = {{k}_{{\mathrm{spli}}}} \cdot P1\_0\_1 - {{k}_{{\mathrm{skip}}}} \cdot {\mathrm{skipping}}}\\ {\displaystyle\frac{{d{\mathrm{fullIR}}}}{{dt}} = {{k}_{{\mathrm{ret}}}} \cdot (P0\_0\_0 + P1\_0\_0 + P0\_1\_0 + P0\_0\_1} { + P1\_1\_0 + P1\_0\_1 + P0\_1\_1 + P1\_1\_1)}\\ {\qquad\qquad- \left( {{{k}_{{\mathrm{dr1}}}} + {{k}_{{\mathrm{dr2}}}}} \right) \cdot {\mathrm{fullIR}}}\\ {\displaystyle\frac{{d1IR}}{{dt}} = {{k}_{{\mathrm{ret}}}} \cdot \left( {P0\_11 + P1\_11} \right) - {{k}_{{\mathrm{dr1}}}} \cdot 1IR}\\ {\displaystyle\frac{{d2IR}}{{dt}} = {{k}_{{\mathrm{ret}}}} \cdot \left( {P11\_0 + P11\_1} \right) - {{k}_{{\mathrm{dr2}}}} \cdot 2IR}\\ \end{array}
\end{eqnarray*}

There are, in general, 13 parameters in the one-step model, but to avoid overfitting, we used a fixed value for all spliceosome dissociation rates, i.e. ${{k}_4} = {{k}_5} = {{k}_6} = 0.01\ {\mathrm{per\ second}}$, as well as RNA synthesis rate *s* = 0.001 per second. Thus, the one-step model has a total of nine independent parameters. The steady-state solution of the final splicing products is used to compare with the data.

### Two-step AE definition model

Next, we extended the one-step model by considering stepwise exon definition, which was suggested in several studies that multiple rate-limiting steps may be required for a committed exon definition [14–16]. To parsimoniously model the stepwise cross-exon complex assembly and remodeling, we assume that AE definition requires two consecutive steps while each outer exon undergoes one-step definition (Supplementary Fig. S5B). The two-step AE definition may involve not only early spliceosome components, such as U1 and U2, but also factors at relatively late stages in the spliceosome cycle, such as U5.U4/U6 tri-snRNP. We used ${{k}_{2{\mathrm{a}}}}$ and ${{k}_{2{\mathrm{b}}}}$ to quantify the spliceosome assembly rates for the first and second AE step, respectively, and assigned ${{k}_{5{\mathrm{a}}}}$ and ${{k}_{5{\mathrm{b}}}}$ as the rates for the corresponding reverse reactions (which were fixed to be 0.01 per second to avoid overfitting, same as in the one-step model).

Compared with the one-step model, the extended model has four additional spliceosome assembly states mediating the two AE definition steps. $P0\_{\mathrm{a}}\_0$, $P1\_{\mathrm{a}}\_0$, $P0\_{\mathrm{a}}\_1$ and $P1\_{\mathrm{a}}\_1$ represent the intermediate AE states with neither, first, last, and both outer exons defined, respectively. These AE intermediate states are not catalytically active for splicing, as no stable or committed spliceosome is formed. Thus, they will be exported from the nucleus and become a full IR isoform if they do not proceed to the second AE definition step. Only the fully defined AE states, such as $P1\_1\_1$, can initiate splicing reactions. The splicing decisions of other spliceosome states as well as the degradation of the spliced isoforms, follow the same mechanisms aspt for the one-step model. The ODEs for the two-step model read,


\begin{eqnarray*}
\begin{array}{@{}*{1}{l}@{}}{\displaystyle\frac{{dP0\_0\_0}}{{dt}} = s + {{k}_4} \cdot P1\_0\_0 + {{k}_{5a}} \cdot P0\_a\_0 + {{k}_6} \cdot P0\_0\_1} { - \left( {{{k}_1} + {{k}_{2a}} + {{k}_3} + {{k}_{{\mathrm{ret}}}}} \right) \cdot P0\_0\_0}\\{\displaystyle\frac{{dP1\_0\_0}}{{dt}} = {{k}_1} \cdot P0\_0\_0 - \left( {{{k}_{2a}} + {{k}_3} + {{k}_4} + {{k}_{{\mathrm{ret}}}}} \right) \cdot P1\_0\_0} { {+{k}_{5a}} \cdot P1\_a\_0 + {{k}_6} \cdot P1\_0\_1}\\{\displaystyle\frac{{dP0\_a\_0}}{{dt}} = {{k}_{2a}} \cdot P0\_0\_0} { - \left( {{{k}_1} + {{k}_3} + {{k}_{2b}} + {{k}_{5a}} + {{k}_{{\mathrm{ret}}}}} \right) \cdot P0\_a\_0} { + {{k}_4} \cdot P1\_a\_0 + {{k}_{5b}} \cdot P0\_1\_0 + {{k}_6} \cdot P0\_a\_1}\\{\displaystyle\frac{{dP0\_1\_0}}{{dt}} = {{k}_{2b}} \cdot P0\_a\_0 - \left( {{{k}_1} + {{k}_3} + {{k}_{5b}} + {{k}_{{\mathrm{ret}}}}} \right) \cdot P0\_1\_0} { + {{k}_4} \cdot P1\_1\_0 + {{k}_6} \cdot P0\_1\_1}\\{\displaystyle\frac{{dP0\_0\_1}}{{dt}} = {{k}_3} \cdot P0\_0\_0 - \left( {{{k}_1} + {{k}_{2a}} + {{k}_6} + {{k}_{{\mathrm{ret}}}}} \right) \cdot P0\_0\_1} { + {{k}_4} \cdot P1\_0\_1 + {{k}_{5a}} \cdot P0\_a\_1}\\{\displaystyle\frac{{dP1\_a\_0}}{{dt}} = {{k}_1} \cdot P0\_a\_0 + {{k}_{2a}} \cdot P1\_0\_0} { - \left( {{{k}_{2b}} + {{k}_3} + {{k}_4} + {{k}_{5a}} + {{k}_{{\mathrm{ret}}}}} \right) \cdot P1\_a\_0} { + {{k}_{5b}} \cdot P1\_1\_0 + {{k}_6} \cdot P1\_a\_1}\\{\displaystyle\frac{{dP1\_1\_0}}{{dt}} = {{k}_1} \cdot P0\_1\_0 + {{k}_{2b}} \cdot P1\_a\_0} { - \left( {{{k}_3} + {{k}_4} + {{k}_{5b}} + {{k}_{{\mathrm{ret}}}} + {{k}_{{\mathrm{spli}}}}} \right) \cdot P1\_1\_0} { + {{k}_6} \cdot P1\_1\_1}\\{\displaystyle\frac{{dP1\_0\_1}}{{dt}} = {{k}_1} \cdot P0\_0\_1 + {{k}_3} \cdot P1\_0\_0} { - \left( {{{k}_{2a}} + {{k}_4} + {{k}_6} + {{k}_{{\mathrm{ret}}}} + {{k}_{{\mathrm{spli}}}}} \right) \cdot P1\_0\_1} { + {{k}_{5a}} \cdot P1\_a\_1}\end{array}
\end{eqnarray*}

(2) \begin{eqnarray*}
\begin{array}{@{}*{1}{l}@{}}{\displaystyle\frac{{dP0\_a\_1}}{{dt}} = {{k}_{2a}} \cdot P0\_0\_1 + {{k}_3} \cdot P0\_a\_0} { - \left( {{{k}_1} + {{k}_{2b}} + {{k}_{5a}} + {{k}_6} + {{k}_{{\mathrm{ret}}}}} \right) \cdot P0\_a\_1} {+ {{k}_4} \cdot P1\_a\_1 + {{k}_{5b}} \cdot P0\_1\_1}\\{\displaystyle\frac{{dP0\_1\_1}}{{dt}} = {{k}_{2b}} \cdot P0\_a\_1 + {{k}_3} \cdot P0\_1\_0} {- \left( {{{k}_1} + {{k}_{5b}} + {{k}_6} + {{k}_{{\mathrm{ret}}}} + {{k}_{{\mathrm{spli}}}}} \right) \cdot P0\_1\_1} { + {{k}_4} \cdot P1\_1\_1}\\{\displaystyle\frac{{dP1\_a\_1}}{{dt}} = {{k}_1} \cdot P0\_a\_1 + {{k}_{2a}} \cdot P1\_0\_1 + {{k}_3} \cdot P1\_a\_0} { - ({{k}_{2b}} + {{k}_4} + {{k}_{5a}} + {{k}_6} + {{k}_{{\mathrm{ret}}}}) \cdot P1\_a\_1} { + {{k}_{5b}} \cdot P1\_1\_1}\\{\displaystyle\frac{{dP1\_1\_1}}{{dt}} = {{k}_1} \cdot P0\_1\_1 + {{k}_{2b}} \cdot P1\_a\_1 + {{k}_3} \cdot P1\_1\_0} { - \left( {{{k}_4} + {{k}_{5b}} + {{k}_6} + {{k}_{{\mathrm{ret}}}} + 2 \cdot {{k}_{{\mathrm{spli}}}}} \right) \cdot P1\_1\_1}\\{\displaystyle\frac{{dP11\_0}}{{dt}} = {{k}_{{\mathrm{spli}}}} \cdot P1\_1\_0 + {{k}_4} \cdot P1\_1\_1} { - \left( {{{k}_1} + {{k}_{{\mathrm{ret}}}}} \right) \cdot P11\_0}\\{\displaystyle\frac{{dP11\_1}}{{dt}} = {{k}_1} \cdot P1\_1\_0 + {{k}_{{\mathrm{spli}}}} \cdot P1\_1\_1} { - \left( {{{k}_4} + {{k}_{{\mathrm{spli}}}} + {{k}_{{\mathrm{ret}}}}} \right) \cdot P11\_1}\\{\displaystyle\frac{{dP0\_11}}{{dt}} = {{k}_{{\mathrm{spli}}}} \cdot P0\_1\_1 + {{k}_6} \cdot P1\_1\_1} { - \left( {{{k}_3} + {{k}_{{\mathrm{ret}}}}} \right) \cdot P0\_11}\\{\displaystyle\frac{{dP1\_11}}{{dt}} = {{k}_3} \cdot P0\_1\_1 + {{k}_{{\mathrm{spli}}}} \cdot P1\_1\_1} { - \left( {{{k}_6} + {{k}_{{\mathrm{spli}}}} + {{k}_{{\mathrm{ret}}}}} \right) \cdot P1\_11}\\{\displaystyle\frac{{d{\mathrm{inclusion}}}}{{dt}} = {{k}_{{\mathrm{spli}}}} \cdot \left( {P1\_11 + P11\_1} \right) - {{k}_{{\mathrm{incl}}}} \cdot {\mathrm{inclusion}}}\\{\displaystyle\frac{{d{\mathrm{skipping}}}}{{dt}} = {{k}_{{\mathrm{spli}}}} \cdot P1\_0\_1 - {{k}_{{\mathrm{skip}}}} \cdot {\mathrm{skipping}}}\\{\displaystyle\frac{{d{\mathrm{fullIR}}}}{{dt}} = {{k}_{{\mathrm{ret}}}} \cdot (P0\_0\_0 + P1\_0\_0 + P0\_1\_0 + P0\_0\_1} { + P1\_1\_0 + P1\_0\_1 + P0\_1\_1 + P1\_1\_1} {+ P0\_a\_0}\\ {\qquad\qquad +\, P1\_a\_0 + P0\_a\_1 + P1\_a\_1)} { - \left( {{{k}_{{\mathrm{dr1}}}} + {{k}_{{\mathrm{dr2}}}}} \right) \cdot {\mathrm{fullIR}}}\\{\displaystyle\frac{{d1IR}}{{dt}} = {{k}_{{\mathrm{ret}}}} \cdot \left( {P0\_11 + P1\_11} \right) - {{k}_{{\mathrm{dr1}}}} \cdot 1IR}\\{\displaystyle\frac{{d2IR}}{{dt}} = {{k}_{{\mathrm{ret}}}} \cdot \left( {P11\_0 + P11\_1} \right) - {{k}_{{\mathrm{dr2}}}} \cdot 2IR}\end{array}
\end{eqnarray*}

The two-step model has just one more free parameter than the one-step model (${{k}_{2{\mathrm{a}}}}$ and ${{k}_{2{\mathrm{b}}}}$compared to ${{k}_{2}}$ in the one-step model; two reverse reaction rates ${{k}_{5{\mathrm{a}}}}$ and ${{k}_{5{\mathrm{b}}}}$ being fixed to 0.01 per second), for a total of 10 independent parameters. The detailed descriptions of all parameters in both models are listed in Supplementary Table S3. The steady-state solution of the final splicing products is used to compare with the data.

### Error model

To robustly quantify the uncertainty in *RON* isoform frequencies, we implemented an error model based on the three replicates of WT and single mutants of the *RON* minigene in HEK293T cells. The error model is based on the statistical nature of the observables. PSI or isoform frequencies as proportions follow the scaling law [44–46], in which the isoform frequency uncertainty distribution is generally not symmetric except around PSI = 50%. However, the skewness of the error distribution also depends on the amplitude of the uncertainty (or perturbations). The distribution is highly skewed (with long tails) near 0 or 100% isoform frequency when the error is very large, whereas the distribution is mainly symmetric across all frequency values if the error is small (Supplementary Fig. S6A). In our *RON* dataset, the standard deviations of isoform frequencies in most single mutants are well below 15% (Supplementary Fig. S6B-G), which suggests a symmetric error distribution. To directly test this, we calculated the isoform frequency errors (each replicate minus the mean value; *n* = 3) from the data and plotted them against the frequency means. The errors indeed were distributed symmetrically across all frequency levels for every isoform (Supplementary Fig. S6A). This symmetric error distribution supports the use of normally distributed standard deviations, which are quantified by the error model, to weigh the distance between the experimental data and the mechanistic exon definition models (Chi-square method).

The RNA-seq data of *RON* minigenes (with randomly combined mutations) were already processed with a 100-read cutoff, which effectively reduces the uncertainty in isoform quantification [26]. To test whether the read coverage affects isoform frequency errors, we calculated the correlation coefficient between the standard deviation of isoform frequency and the isoform sequencing read counts and found little correlation between the two (Supplementary Fig. S6G). Next, we performed the same correlation analysis using more strict read cutoffs (200, 300, 400, and 500) and arrived at the same conclusion (Supplementary Fig. S6G). Therefore, the large read coverage cutoff used in our RNA-seq data quantification greatly reduces the isoform frequency uncertainty.

Though all splice isoforms in the *RON* dataset show a qualitatively similar, bell-shaped error-mean relation, the isoforms exhibit quantitative differences in their error model (Supplementary Fig. S6C-G). Using the coverage analyses above, we excluded that isoform-specific errors arise from isoform-specific read coverage (Supplementary Fig. S6H and I).

Based on the analyses above, we employed the isoform-specific error model based on the scaling law [26, 44, 45]:


(3) \begin{eqnarray*}
{\mathrm{st}}{{{\mathrm{d}}}_i} = \frac{{{\mathrm{mea}}{{{\mathrm{n}}}_i}}}{{{\mathrm{mea}}{{{\mathrm{n}}}_i} + \left( {1 - {\mathrm{mea}}{{{\mathrm{n}}}_i}} \right) \cdot {{\alpha }_i}}} - {\mathrm{mea}}{{{\mathrm{n}}}_i} + {{b}_{0,i}}
\end{eqnarray*}

where *i* is the index for five *RON* isoforms. The factor *α* determines the amplitude and shape of the error-mean scaling curve (Supplementary Fig. S6A). The intercept *b*_0_ describes the absolute error independent of the mean isoform frequency. We applied this calibrated error model to quantify the weights of all isoform frequencies in constructing the objective function for fitting the one-step and two-step models to the *RON* data (HEK293 and RBP KD datasets). The model fitting was performed using the lsqnonlin function in MATLAB.

### Detecting signatures of nonsense-mediated decay in the *RON* minigene

Splice isoforms of the *RON* minigene may exhibit different half-lives. Especially if the minigene transcripts are subject to translation and contain premature stop codons, nonsense-mediated decay (NMD) might lead to strong shifts in isoform distributions.

To address whether NMD occurs in the *RON* minigene, we analyzed (i) whether the requirements of NMD are fulfilled in the wt sequence; (ii) whether stop codons in mutated minigene variants lead to strong shifts in isoform abundance. Specifically, we reasoned that stop codons in the AE should lead to degradation of the inclusion isoform if NMD occurs, thereby giving rise to a strong dominance of skipping.

(i) No indication for NMD in the wt *RON* minigene: We found that the wt sequence contains no in-frame stop codon in the inclusion and skipping isoforms, whereas full IR, first IR, and second IR contain early stop codons. Since NMD requires the deposition of an EJC, i.e. a successful splicing event downstream of a stop codon [47–49], only the first IR is a possible candidate for NMD [47, 50, 51]. However, NMD is still unlikely to occur in the wt *RON* sequence, since in-frame start codons giving rise to ORFs and thus active translation are not observed in *RON* exons 10–12. Even though the plasmid sequence contains a potential in-frame start codon upstream of the *RON* minigene, this is located significantly upstream (100 nt) of the putative transcription start site, and therefore very likely not relevant for translation and NMD. All in all, this suggests that translation and NMD do not occur in any of the *RON* minigene transcript isoforms.

(ii) Exon 2 stop mutations affect splice-regulatory elements, but do not lead to NMD: To further validate the claim that premature stop codons do not trigger transcript degradation, we analyzed mutated *RON* minigenes, searching for the emergence of in-frame STOP codons in exon 2. In the presence of NMD, these should lead to degradation of the inclusion but not the skipping isoform, thus shifting the PSI (= inclusion/(inclusion + skipping)). We found that the inclusion isoform contains a premature stop codon in 94 out of 5791 minigene variants, suggesting that NMD would, in any case, be a rare phenomenon. The PSI in this minigene population is substantially lower than in minigenes harboring no stop codons or those harboring stop codons in exons 10 or 12, which would simultaneously affect both inclusion and skipping degradation. Thus, it is possible that stop codons in exon 2 indeed trigger NMD or that they perturb splicing-effective *cis*-regulatory elements.

To distinguish the scenarios, we performed a closer inspection and found that the PSI shift in the small population of 94 minigenes with stop codons in exon 2 mainly relies on a bias from two particular point mutations (C415T, C442T). One of these mutations is located 1 nt away from the downstream splice site of exon 2 (C442T), and another substitution at the same position that does not give rise to a stop codon (C442G) gives rise to a similar PSI drift, suggesting a splicing change and excluding a pure NMD effect (Supplementary Data 4 in Braun *et al*., 2018) [26]. The other mutation (C415T) lies within a putative exonic splicing enhancer in which several nearby mutations (T413G, A416C) similarly lower the PSI (see Supplementary Data 4 in Braun *et al*., 2018) [26]. Thus, these mutation effects are consistent with splicing regulation, but do not necessarily support NMD. Accordingly, for the remaining four mutations that introduce a stop in exon 11 in more than 5 minigenes (T327A, C363A, C364T, T440A) no or much weaker PSI drifts are observed in the primary minigene data (Supplementary Data 4 in Braun *et al*., 2018) [26]. Taken together, these analyses suggest that mutations affect splice-regulatory elements, not NMD.

### Quantitative comparison between one-step and two-step AE definition models

To quantitatively compare the one-step and two-step models, we fit both models to the data recording the frequencies of the five isoforms of *RON* minigene in WT and 405 single mutants by minimizing the standard ${{\chi }^2}$ metric,


(4) \begin{eqnarray*}
{{{{\chi }}}^2} &=& \mathop \sum \limits_{{{i}} = 1}^5 {{\left( {\frac{{{{X}}_{{{wt}}}^{{{isoform}}{\mathrm{\ }}{{i}}} - {{Y}}_{{{wt}}{\mathrm{\ }}}^{{{isoform}}{\mathrm{\ }}{{i}}}}}{{{{\sigma }}_{{{wt}}}^{{{isoform}}{\mathrm{\ }}{{i}}}}}} \right)}^2} + \mathop \sum \limits_{{{j}} = 1}^{{M}} \mathop \sum \limits_{{{i}} = 1}^5 {{\left( {\frac{{{{X}}_{{{mutant}}{\mathrm{\ }}{{j}}}^{{{isoform}}{\mathrm{\ }}{{i}}} - {{Y}}_{{{mutant}}{\mathrm{\ }}{{j}}}^{{{isoform}}{\mathrm{\ }}{{i}}}}}{{{{\sigma }}_{{{mutant}}{\mathrm{\ }}{{j}}}^{{{isoform}}{\mathrm{\ }}{{i}}}}}} \right)}^2}
\end{eqnarray*}

where $X_{{\mathrm{wt}}}^{{\mathrm{isoform}}\ i}$ is the mean frequency of WT *RON* isoform *i*, with *i* = 1, …, 5 representing AE inclusion, AE skipping, first, second and full intron retention isoforms, respectively, and $Y_{{\mathrm{wt}}}^{{\mathrm{isoform}}\ i}$ is the computed frequency of the same isoform ($X_{{\mathrm{wt}}}^{{\mathrm{isoform}}\ i}$) from the one-step or two-step model. $\sigma _{{\mathrm{wt}}}^{{\mathrm{isoform}}\ i}$ is the standard deviation of the frequency of WT *RON* isoform *i* calculated by the error model at the mean value $X_{{\mathrm{wt}}}^{{\mathrm{isoform}}\ i}$. $X_{{\mathrm{mutant}}\ j}^{{\mathrm{isoform}}\ i}$, $Y_{{\mathrm{mutant}}\ j}^{{\mathrm{isoform}}\ i}$ and $\sigma _{{\mathrm{mutant}}\ j}^{{\mathrm{isoform}}\ i}$ are the counterparts of $X_{{\mathrm{wt}}}^{{\mathrm{isoform}}\ i}$, $Y_{{\mathrm{wt}}}^{{\mathrm{isoform}}\ i}$ and $\sigma _{{\mathrm{wt}}}^{{\mathrm{isoform}}\ i}$ in the single mutant *j*, respectively, where in total 405 effective single mutations were included in the model fitting.

To constrain the parameters for the mutation effects, we assume that a single mutation only affects the definition of its residing (if the mutation is exonic) or nearest (if the mutation is intronic) exon. Therefore, each mutant and WT differ only in one exon definition parameter (${{k}_1}$, ${{k}_2}$ or ${{k}_3}$) and share all the other parameters. For the two-step model, a mutation affects both definition rates (${{k}_{2{\mathrm{a}}}}$ and ${{k}_{2{\mathrm{b}}}}$) by the same factor, leaving their ratio unchanged (as in the WT). In total, 413 independent parameters for the one-step exon definition and 414 for the two-step exon definition model 413 independent parameters (Supplementary Table S3) were inferred from 2030 data points. The computational inference was performed using Data2Dynamics (D2D), an open source MATLAB package [52]. To search for the best parameters, we employed the Latin hypercube sampling (1000 samples) for the starting parameter values.

### Identifying regulatory roles of RBPs by model discrimination

RBPs are known to regulate different splicing events, but it is often difficult to pinpoint which specific splicing steps are modulated by RBPs. It is also not clear how RBPs control the coordinated AE and IR decisions. To identify the RBPs’ regulatory modes for *RON* minigene, we derived a family of two-step AE definition model variants, where an RBP may modulate individual or all possible combinations of the definition steps of the three exons. To compare these models to RBP KD data, we parameterized the models as follows:


(5) \begin{eqnarray*}
\begin{array}{@{}*{1}{l}@{}} {{{k}_{1,{\mathrm{KD}}}} = {{ \epsilon }^i}{{k}_1}}\\ {{{k}_{2{\mathrm{a}},{\mathrm{KD}}}} = {{ \epsilon }^j}{{k}_{\mathrm{2a}}}}\\ {{{k}_{2{\mathrm{b}},{\mathrm{KD}}}} = {{ \epsilon }^m}{{k}_{\mathrm{2b}}}}\\ {{{k}_{3,{\mathrm{KD}}}} = {{ \epsilon }^n}{{k}_3}} \end{array}
\end{eqnarray*}

where ${{k}_{x,{\mathrm{KD}}}}$ (*x* = 1, 2a, 2b, and 3) is the rate of exon definition step *x* upon RBP KD from the WT *RON* minigene (without mutations). $\epsilon $ quantifies the effect of RBP KD on individual exon definition rates. Binary integers *i, j, m*, and *n* indicate whether an RBP modulates an exon definition step (values being 1) or not (being 0). To avoid parameter non-identifiability, we used the same $\epsilon $ value for multiple steps when an RBP regulates all these steps. We derived a total of 15 (2^4^ – 1) model variants describing different regulatory modes of an RBP.

Next, we quantitatively select the best models that faithfully capture the KD data of five RBPs (PRPF6, PUF60, HNRNPH1, SMU1, and SRSF2). For each RBP KD, we fit all 15 model variants to the data of RBP KD control (without mutations) and cross-validate the results by predicting the single-mutation effects (Supplementary Table S4). The single mutation effects (on the exon definition parameters) inferred from mutagenesis data of WT *RON* minigene (Supplementary Table S3) were used for the model prediction. The goodness of fit and prediction were quantified by both the root mean square error (RMSE) and the ${{\chi }^2}$ value relative to the critical ${{\chi }^2}$ value defined by the degree of freedom of each model variant at 95% confidence level (Supplementary Fig. S9). This approach identifies the effects of RBP KDs on splicing outcomes and thus provides a systematic understanding of splicing regulation by different perturbations.

### Toy models of multistep AE recognition

To systematically investigate how multistep AE recognition affects AE inclusion and splicing efficiency, we implemented toy models with a varying number of recognition steps (from 1 to 7).

In each model variant, we assume rapid and complete definition of the outer exons and hence consider an initial species, in which the constitutive exons are defined but the AE is undefined (P101). P101 is produced at a constant rate, which we, for simplicity, assume to be 1 in all models. On the way to the final species with a fully defined AE (P111) there are one (one-step model), two (two-step model), or *n* (*n*-step model) irreversible AE recognition steps, proceeding with the first-order rate constant *α*^i-1^*k*_2_ for the *i*-th step. Here, the cooperativity factor *α* reflects that the AE recognition may be progressively faster (*α* > 1) or slower (*α* < 1) along the multistep recognition chain, which proceeds through the partially defined AE states termed P1a1, P1b1, …, P1{n-1}1. In molecular terms, *α* < 1 implies that late spliceosome assembly steps during AE recognition are slower than the early ones (and the opposite is true for *α* > 1).

In line with the full splicing model introduced in Supplementary Fig. S5, we assume that productive splicing is not possible in the partially defined species. Hence, while intron retention is produced from all intermediates in the models (P101, P1a1, P1b1, …, P1{n-1}1, P111) with the first-order rate constant *k*_ret_, AE skipping and inclusion only arise with first-order kinetics (*k*_spl_) from p101 and p111, respectively.

For the one-step model, the differential equations are given by:


(6) \begin{eqnarray*}
\begin{array}{@{}*{2}{l}@{}} {\displaystyle\frac{{dP101}}{{dt}}}&{ = 1 - \left( {{{k}_{{\mathrm{ret}}}} + {{k}_2} + {{k}_{{\mathrm{spl}}}}} \right)P101}\\ {\displaystyle\frac{{dP111}}{{dt}}}&{ = {{k}_2}{{P}_{101}} - \left( {{{k}_{{\mathrm{ret}}}} + {{k}_{{\mathrm{spl}}}}} \right)P111} \end{array}
\end{eqnarray*}

Setting the above equations to zero yields the steady-state levels of P101 and P111:


(7) \begin{eqnarray*}
\begin{array}{@{}*{2}{l}@{}} {P101}&{ = \displaystyle\frac{1}{{{{k}_{{\mathrm{spl}}}} + {{k}_{{\mathrm{ret}}}} + {{k}_2}}},}\\ {P111}&{ = \displaystyle\frac{{{{k}_2}{{P}_{101}}}}{{{{k}_{{\mathrm{spl}}}} + {{k}_{{\mathrm{ret}}}}}}.} \end{array}
\end{eqnarray*}

Using Equation 7, we can calculate PSI and efficiency. To this end, we define PSI as the fraction of inclusion over all productive isoforms and efficiency as the fraction of productive isoforms over all terminal splicing outcomes:


(8) \begin{eqnarray*}
\begin{array}{@{}*{2}{l}@{}} {{\mathrm{PSI}}}&{ = \displaystyle\frac{{{\mathrm{inclusion}}}}{{{\mathrm{inclusion}} + {\mathrm{skipping}}}}}\\ {{\mathrm{Efficiency}}}&{ = \displaystyle\frac{{{\mathrm{inclusion}} + {\mathrm{skipping}}}}{{{\mathrm{inclusion}} + {\mathrm{skipping}} + {\mathrm{retention}}}}} \end{array}
\end{eqnarray*}

Using the equations above, we obtain:


(9) \begin{eqnarray*}
\begin{array}{@{}*{2}{l}@{}} {{\mathrm{PSI}}}&{ = \displaystyle\frac{{{{k}_2}}}{{{{k}_2} + {{k}_{{\mathrm{spl}}}} + {{k}_{{\mathrm{ret}}}}}}}\\ {{\mathrm{Efficiency}}}&{ = \displaystyle\frac{{{{k}_{{\mathrm{spl}}}}}}{{{{k}_{{\mathrm{ret}}}} + {{k}_{{\mathrm{spl}}}}}}} \end{array}
\end{eqnarray*}

From Equation 9, increasing AE recognition *k*_2_ leads to an increase in PSI with a Michaelis-Menten-type curve (half-saturation point: *k*_2_ = *k*_spl_ + *k*_ret_). Hence, the PSI curve as a function of *k*_2_ exhibits an effective Hill coefficient *h* = 1. On the other hand, the splicing efficiency is independent of *k*_2_. It is determined by the competing rates of retention (*k*_ret_) and productive splicing (*k*_spl_). Hence, the one-step model shows no splicing efficiency drop at intermediate *k*_2_.

Likewise, for the two-step model, the differential equations are given by,


(10) \begin{eqnarray*}
\begin{array}{@{}*{2}{l}@{}} {\displaystyle\frac{{dP101}}{{dt}}}&{ = 1 - \left( {{{k}_{{\mathrm{ret}}}} + {{k}_2} + {{k}_{{\mathrm{spl}}}}} \right)P101}\\ {\displaystyle\frac{{dP1{\mathrm{a}}1}}{{dt}}}&{ = {{k}_2}{\mathrm{P}}101 - \left( {{{k}_{{\mathrm{ret}}}} + \alpha {{k}_2}} \right){\mathrm{P}}1{\mathrm{a}}1}\\ {\displaystyle\frac{{dP111}}{{dt}}}&{ = \alpha {{k}_2}{\mathrm{P}}1{\mathrm{a}}1 - \left( {{{k}_{{\mathrm{ret}}}} + {{k}_{{\mathrm{spl}}}}} \right){\mathrm{P}}111} \end{array}
\end{eqnarray*}

The steady-state equations are:


(11) \begin{eqnarray*}
\begin{array}{@{}*{2}{l}@{}} {P101}&{ = \displaystyle\frac{1}{{{{k}_{{\mathrm{spl}}}} + {{k}_{{\mathrm{ret}}}} + {{k}_2}}}}\\ {P1a1}&{ = \displaystyle\frac{{{{k}_2}P1\_0\_1}}{{\alpha {{k}_2} + {{k}_{{\mathrm{ret}}}}}} = \displaystyle\frac{{{{k}_2}}}{{\left( {\alpha {{k}_2} + {{k}_{{\mathrm{ret}}}}} \right)\left( {{{k}_{{\mathrm{spl}}}} + {{k}_{{\mathrm{ret}}}} + {{k}_2}} \right)}}}\\ {P111}&{ = \displaystyle\frac{{\alpha {{k}_2}P1a1}}{{{{k}_{{\mathrm{spl}}}} + {{k}_{{\mathrm{ret}}}}}} = \displaystyle\frac{{\alpha k_2^2}}{{\left( {{{k}_{{\mathrm{spl}}}} + {{k}_{{\mathrm{ret}}}}} \right)\left( {\alpha {{k}_2} + {{k}_{{\mathrm{ret}}}}} \right)\left( {{{k}_{{\mathrm{spl}}}} + {{k}_{{\mathrm{ret}}}} + {{k}_2}} \right)}}} \end{array}
\end{eqnarray*}

According to Equation 8, PSI and efficiency read:


(12) \begin{eqnarray*}
\begin{array}{@{}*{2}{l}@{}} {{\mathrm{PSI}}}&{ = \displaystyle\frac{{\alpha k_2^2}}{{\alpha k_2^2 + \left( {\alpha {{k}_2} + {{k}_{{\mathrm{ret}}}}} \right)\left( {{{k}_{{\mathrm{spl}}}} + {{k}_{{\mathrm{ret}}}}} \right)}},}\\ {{\mathrm{Efficiency}}}&{ = \displaystyle\frac{{{{k}_{{\mathrm{spl}}}}\left( {P1\_0\_1 + P1\_1\_1} \right)}}{{{{k}_{{\mathrm{spl}}}}\left( {P1\_0\_1 + P1\_1\_1} \right) + {{k}_{{\mathrm{ret}}}}\left( {P1\_0\_1 + P1\_a\_1 + P1\_1\_1} \right)}}.} \end{array}
\end{eqnarray*}

To estimate the Hill coefficient of the PSI response curve, we used the following formula from the literature [53]:


(13) \begin{eqnarray*}
h = \frac{{{\mathrm{log}}\left( {81} \right)}}{{{\mathrm{log}}\left( {\frac{{{{k}_{2,90}}}}{{{{k}_{2,10}}}}} \right)}},
\end{eqnarray*}

where *k*_2,10_ and *k*_2,90_ are the values of the AE recognition parameter required to reach PSI = 10% and PSI = 90%, respectively. For a very switch-like response, *k*_2,10_ and *k*_2,90_ are close, leading to a high Hill coefficient according to Equation 13.

In Equation 12, the PSI curve of the two-step model is a Hill-like equation in *k*_2_, in which the denominator contains a mixture of linear and quadratic *k*_2_-terms. For large *α*, i.e. *αk*_2_ >> *k*_ret_ this equation simplifies to Equation 9 describing PSI of the one-step model. For small *α* (slow second step), the linear term can be neglected, and the equation approaches a Hill equation with a Hill coefficient of 2:


(14) \begin{eqnarray*}
{\mathrm{PSI}} \approx \frac{{\alpha k_2^2}}{{\alpha k_2^2 + {{k}_{{\mathrm{ret}}}}( {{{k}_{{\mathrm{spl}}}} + {{k}_{{\mathrm{ret}}}}} )}}
\end{eqnarray*}

Therefore, the two-step model exhibits a Hill coefficient between *h* = 1 and *h* = 2. In contrast to the one-step model (Equation 9), the efficiency of the two-step model (Equation 12) is no longer independent of *k*_2_, exhibiting a drop at intermediate PSI values.

For the *n*-step model, we can write a similar differential equation system as above and obtain the following steady state equations:


(15) \begin{eqnarray*}
\begin{array}{@{}*{1}{l}@{}} {P101 = \displaystyle\frac{1}{{{{k}_{{\mathrm{spl}}}} + {{k}_{{\mathrm{ret}}}} + {{k}_2}}}}\\ {P1a1 = \displaystyle\frac{{{{k}_2}P101}}{{\alpha {{k}_2} + {{k}_{{\mathrm{ret}}}}}} = \displaystyle\frac{{{{k}_2}}}{{\left( {\alpha {{k}_2} + {{k}_{{\mathrm{ret}}}}} \right)\left( {{{k}_{{\mathrm{spl}}}} + {{k}_{{\mathrm{ret}}}} + {{k}_2}} \right)}}}\\ {P1b1 = \displaystyle\frac{{\alpha {{k}_2}P1a1}}{{{{\alpha }^2}{{k}_2} + {{k}_{{\mathrm{ret}}}}}} = \displaystyle\frac{{\alpha k_2^2}}{{\left( {{{\alpha }^2}{{k}_2} + {{k}_{{\mathrm{ret}}}}} \right)\left( {\alpha {{k}_2} + {{k}_{{\mathrm{ret}}}}} \right)\left( {{{k}_{{\mathrm{spl}}}} + {{k}_{{\mathrm{ret}}}} + {{k}_2}} \right)}}}\\ {P1c1 = \displaystyle\frac{{{{\alpha }^2}{{k}_2}P1b1}}{{{{\alpha }^3}{{k}_2} + {{k}_{{\mathrm{ret}}}}}} } { = \displaystyle\frac{{{{\alpha }^3}k_2^2}}{{\left( {{{\alpha }^3}{{k}_2} + {{k}_{{\mathrm{ret}}}}} \right)\left( {{{\alpha }^2}{{k}_2} + {{k}_{{\mathrm{ret}}}}} \right)\left( {\alpha {{k}_2} + {{k}_{{\mathrm{ret}}}}} \right)\left( {{{k}_{{\mathrm{spl}}}} + {{k}_{{\mathrm{ret}}}} + {{k}_2}} \right)}}}\\ \vdots \\ {P111 = P1n1 = \displaystyle\frac{{{{\alpha }^{n - 1}}k_2^{n - 1}P1\left[ {n - 1} \right]1}}{{{{k}_{{\mathrm{spl}}}} + {{k}_{{\mathrm{ret}}}}}}} { = \displaystyle\frac{{{{\alpha }^{n\left( {n - 1} \right)/2}}k_2^n}}{{\left( {{{k}_{{\mathrm{spl}}}} + {{k}_{{\mathrm{ret}}}}} \right) \cdots \left( {{{\alpha }^2}{{k}_2} + {{k}_{{\mathrm{ret}}}}} \right)\left( {\alpha {{k}_2} + {{k}_{{\mathrm{ret}}}}} \right)\left( {{{k}_{{\mathrm{spl}}}} + {{k}_{{\mathrm{ret}}}} + {{k}_2}} \right)}}.} \end{array}
\end{eqnarray*}

PSI and efficiency read:


(16) \begin{eqnarray*}
\begin{array}{@{}*{1}{l}@{}} {{\mathrm{PSI}} = \displaystyle\frac{{{{{{\alpha }}}^{{{n}}\left( {{{n}} - 1} \right)/2}}{{k}}_2^{{n}}}}{{{{{{\alpha }}}^{{{n}}\left( {{{n}} - 1} \right)/2}}{{k}}_2^{{n}} + \left( {{{{{k}}}_{{\mathrm{spl}}}} + {{{{k}}}_{{\mathrm{ret}}}}} \right) \cdots \left( {{{{{\alpha }}}^2}{{{{k}}}_2} + {{{{k}}}_{{\mathrm{ret}}}}} \right)\left( {{{\alpha }}{{{{k}}}_2} + {{{{k}}}_{{\mathrm{ret}}}}} \right)}},}\\ {{\mathrm{Efficiency}} = \displaystyle\frac{{{{k}_{{\mathrm{spl}}}}\left( {P101 + P111} \right)}}{{{{k}_{{\mathrm{spl}}}}\left( {P101 + P111} \right) + {{k}_{{\mathrm{ret}}}}\left( {P101 + P1a1 + \cdots + P1\left[ {n - 1} \right]1 + P111} \right)}}} \end{array}
\end{eqnarray*}

Equation 16 was used to plot PSI as a function of the AE recognition strength *k*_2_ for varying numbers of AE recognition steps (*n* = 3 – 7), and the effective Hill coefficient was estimated using Equation 13. In the same way, the efficiency curves (*n* = 3 – 7) were obtained by varying *k*_2_, and the efficiency was plotted as a function of PSI. The following parameter values were assumed for all model variants: *k*_spl_ = 1, *k*_ret_ = 0.2 and *α* = 1/30.

According to Equation 16, PSI is a Hill-like equation in *k*_2_ which contains a polynomial of *n*-th order in the denominator. Thus, for a larger number of AE recognition steps (*n*), the Hill coefficient increases but is smaller than the number of molecular steps *n*.

To confirm the robustness of our simulation results concerning switch-like alternative splicing and the splicing efficiency drop, we performed random parameter sampling for the one- to seven-step models. To this end, the model parameters were sampled 2000 times around their starting values (*k*_spl_ = 1, *k*_ret_ = 0.2, and *α* = 1/30), assuming a log-normal distribution with a standard deviation of 0.5.

For each parameter sample, *k*_2_ varied over a broad range, and the effective Hill coefficient *h* of the PSI for varying *k*_2_ was calculated using Equation 13 to plot distributions of the Hill coefficient across the whole parameter sample for each model. In a related fashion, the ratio of *k*_2,90_ and *k*_2,10_, as also defined in the context of Equation 13, is calculated for the whole parameter sample as a more intuitive measure reflecting the change in *k*_2_ required to switch PSI from a low to a high value. The Hill coefficients of the parameter sample are related to the corresponding minimal splicing efficiency, defined as the lowest splicing efficiency across the full range of AE recognition values *k*_2_.

From the parameter sampling, we concluded that the gain in switch-like behavior (*h*) for an increasing number of AE recognition steps (*n*) is limited (*h* << *n* for large *n*). Moreover, switch-like behavior in multistep AE recognition models comes at a trade-off, as the splicing efficiency at intermediate PSI values is reduced for increasing Hill coefficients *h*.

### Modeling transcriptome-wide PSI-efficiency relation in normal and cancer tissues

The dependency of splicing efficiency on AE inclusion (PSI) was also observed as a general trend transcriptome-wide by analyzing RNA-seq data across different tissues (Supplementary Fig. S4). As these data were obtained by short-read sequencing, they lack the information at the RNA isoform level. To model the PSI-efficiency relation, we simplified the full two-step model (Supplementary Fig. S5B) and derived an explicit expression of efficiency as a function of PSI. To this end, we assume that the spliceosome assembly is much more rapid than the splicing catalysis reaction. Thus, the splicing outcome is tightly determined by the spliceosome binding states. Following our previous work on exon definition [31], the recognition probabilities of the two outer exons, *α* (the first exon) and *γ* (the third exon), in the three-exon module of a transcript can be formulated by,


(17) \begin{eqnarray*}
\begin{array}{@{}*{1}{l}@{}} {\alpha = \displaystyle\frac{{{{K}_1}}}{{1 + {{K}_1}}}}\\ {\gamma = \displaystyle\frac{{{{K}_3}}}{{1 + {{K}_3}}}} \end{array}
\end{eqnarray*}

where *K*_1_ (= *k*_1_/*k*_4_) and *K*_3_ (= *k*_3_/*k*_6_) are the association constants of spliceosome binding for exons 1 and 3, respectively. For the sequential two-step AE definition, two recognition probabilities arise,


(18) \begin{eqnarray*}
\begin{array}{@{}*{1}{l}@{}} {{{\beta }_1} = \displaystyle\frac{{{{K}_{2a}}}}{{1 + {{K}_{2a}} + {{K}_{2a}}{{K}_{2b}}}}}\\ {{{\beta }_2} = \displaystyle\frac{{{{K}_{2a}}{{K}_{2b}}}}{{1 + {{K}_{2a}} + {{K}_{2a}}{{K}_{2b}}}}} \end{array}
\end{eqnarray*}

where *β*_1_ and *β*_2_ are the probabilities of partially (after one step) and fully (after two steps) recognized AE, respectively. Using these exon recognition probabilities, we can formulate the frequencies of the spliceosome binding states,


(19) \begin{eqnarray*}
\begin{array}{@{}*{1}{l}@{}} {{{x}_{000}} = \left( {1 - \alpha } \right)\left( {1 - {{\beta }_1} - {{\beta }_2}} \right)\left( {1 - \gamma } \right)}\\ {{{x}_{100}} = \alpha \left( {1 - {{\beta }_1} - {{\beta }_2}} \right)\left( {1 - \gamma } \right)}\\ {{{x}_{001}} = \left( {1 - \alpha } \right)\left( {1 - {{\beta }_1} - {{\beta }_2}} \right)\gamma }\\ {{{x}_{010}} = \left( {1 - \alpha } \right){{\beta }_1}\left( {1 - \gamma } \right)}\\ {{{x}_{020}} = \left( {1 - \alpha } \right){{\beta }_2}\left( {1 - \gamma } \right)}\\ {{{x}_{110}} = \alpha {{\beta }_1}\left( {1 - \gamma } \right)}\\ {{{x}_{011}} = \left( {1 - \alpha } \right){{\beta }_1}\gamma }\\ {{{x}_{120}} = \alpha {{\beta }_2}\left( {1 - \gamma } \right)}\\ {{{x}_{021}} = \left( {1 - \alpha } \right){{\beta }_2}\gamma }\\ {{{x}_{101}} = \alpha \left( {1 - {{\beta }_1} - {{\beta }_2}} \right)\gamma }\\ {{{x}_{111}} = \alpha {{\beta }_1}\gamma }\\ {{{x}_{121}} = \alpha {{\beta }_2}\gamma } \end{array}
\end{eqnarray*}

where *x_ijk_* represents the frequency of an exon-definition state. The binary index *i* indicates whether the first exon is recognized (*i* = 1) or unrecognized (*i* = 0). In the same way, the subscript *k* describes the definition state of the third exon. For the AE, the index *j* has three values, 0, 1, and 2, indicating undefined, partially, and fully defined AE states, respectively. The splicing active states *x*_121_, *x*_101_, *x*_021_, and *x*_120_ give rise to AE inclusion, skipping, first and second intron retention isoforms, respectively. We assume that the pre-mRNA nuclear export rate (*k*_ret_; Supplementary Fig. S5B) is very low, and the remaining eight states are not productive and become full intron retention products.

Using the same definitions for PSI and efficiency as in previous sections,


(20) \begin{eqnarray*}
\begin{array}{@{}*{1}{l}@{}} {{\mathrm{PSI}} = \displaystyle\frac{{{\mathrm{inclusion}}}}{{{\mathrm{inclusion}} + {\mathrm{skipping}}}}}\\ {{\mathrm{efficiency}} = {\mathrm{inclusion}} + {\mathrm{skipping}}} \end{array}
\end{eqnarray*}

We have,


(21) \begin{eqnarray*}
\begin{array}{@{}*{1}{l}@{}} {{\mathrm{PSI}} = \displaystyle\frac{{{{K}_{2{\mathrm{a}}}}{{K}_{2{\mathrm{b}}}}}}{{1 + {{K}_{2{\mathrm{a}}}}{{K}_{2{\mathrm{b}}}}}}}\\ {{\mathrm{efficiency}} = \alpha \gamma \displaystyle\frac{{1 + {{K}_{2{\mathrm{a}}}}{{K}_{2{\mathrm{b}}}}}}{{1 + {{K}_{2{\mathrm{a}}}} + {{K}_{2{\mathrm{a}}}}{{K}_{2{\mathrm{b}}}}}}} \end{array}
\end{eqnarray*}

Let *λ* be the ratio between the second and first AE definition rates,


(22) \begin{eqnarray*}
{{K}_{2{\mathrm{b}}}} = \lambda {{K}_{2{\mathrm{a}}}}
\end{eqnarray*}

then Equation 21 becomes


(23) \begin{eqnarray*}
\begin{array}{@{}*{1}{l}@{}} {{\mathrm{PSI}} = \displaystyle\frac{{\lambda K_{2{\mathrm{a}}}^2}}{{1 + \lambda K_{2{\mathrm{a}}}^2}}}\\ {{\mathrm{efficiency}} = \alpha \gamma \displaystyle\frac{{1 + \lambda K_{2{\mathrm{a}}}^2}}{{1 + {{K}_{2{\mathrm{a}}}} + \lambda K_{2{\mathrm{a}}}^2}}} \end{array}
\end{eqnarray*}

Solving the PSI (in Equation 23) for *K*_2a_ yields,


(24) \begin{eqnarray*}
{{K}_{2{\mathrm{a}}}} = \sqrt {\frac{{{\mathrm{PSI}}}}{{\lambda \left( {1 - {\mathrm{PSI}}} \right)}}}
\end{eqnarray*}

Substituting this result into the equation of efficiency (Equation 23), we obtained efficiency as a function of PSI,


(25) \begin{eqnarray*}
{\mathrm{efficiency}} = \alpha \gamma \frac{1}{{1 + \sqrt {{\mathrm{PSI}}\left( {1 - {\mathrm{PSI}}} \right)/\lambda } }}
\end{eqnarray*}

This equation suggests that the recognition of the two constitutive exons can compensate for each other in shaping the efficiency-PSI dependency (the product of *α* and *γ* as a scaling factor). To avoid parameter non-identifiability, we lumped *αγ* as one parameter, i.e. *αγ *= *δ*^2^, and the final equation is,


(26) \begin{eqnarray*}
{\mathrm{efficiency}} = {{\delta }^2}\frac{1}{{1 + \sqrt {{\mathrm{PSI}}\left( {1 - {\mathrm{PSI}}} \right)/\lambda } }}
\end{eqnarray*}

The simplified model has two independent parameters *λ* and *δ. δ* represents the average recognition probability of the constitutive exons, and it determines the maximum efficiency values at extreme PSIs (0 and 100%). *λ* reflects the relative speed (or cooperativity) of the two consecutive AE recognition steps. It determines the level and position (at which PSI) of the lowest efficiency, and therefore the overall shape of the efficiency-PSI curve.

Next, we use this equation to fit the efficiency-PSI relation to the transcriptome-wide data, where the efficiencies were calculated as the median in the PSI bins (10 bins of equal width). For each of the 20 tissues, the efficiency-PSI data for both normal and malignant conditions were fitted jointly, assuming both parameters (*λ* and *δ*) may be affected in cancerous cells. The best parameters were optimized using the least squares method. The difference in parameters between normal and malignant tissues implies how and which exon definition steps were perturbed in cancers (Supplementary Fig. S10).

## Results

### Alternative exon inclusion and intron retention are coordinately regulated

**Figure 1.**
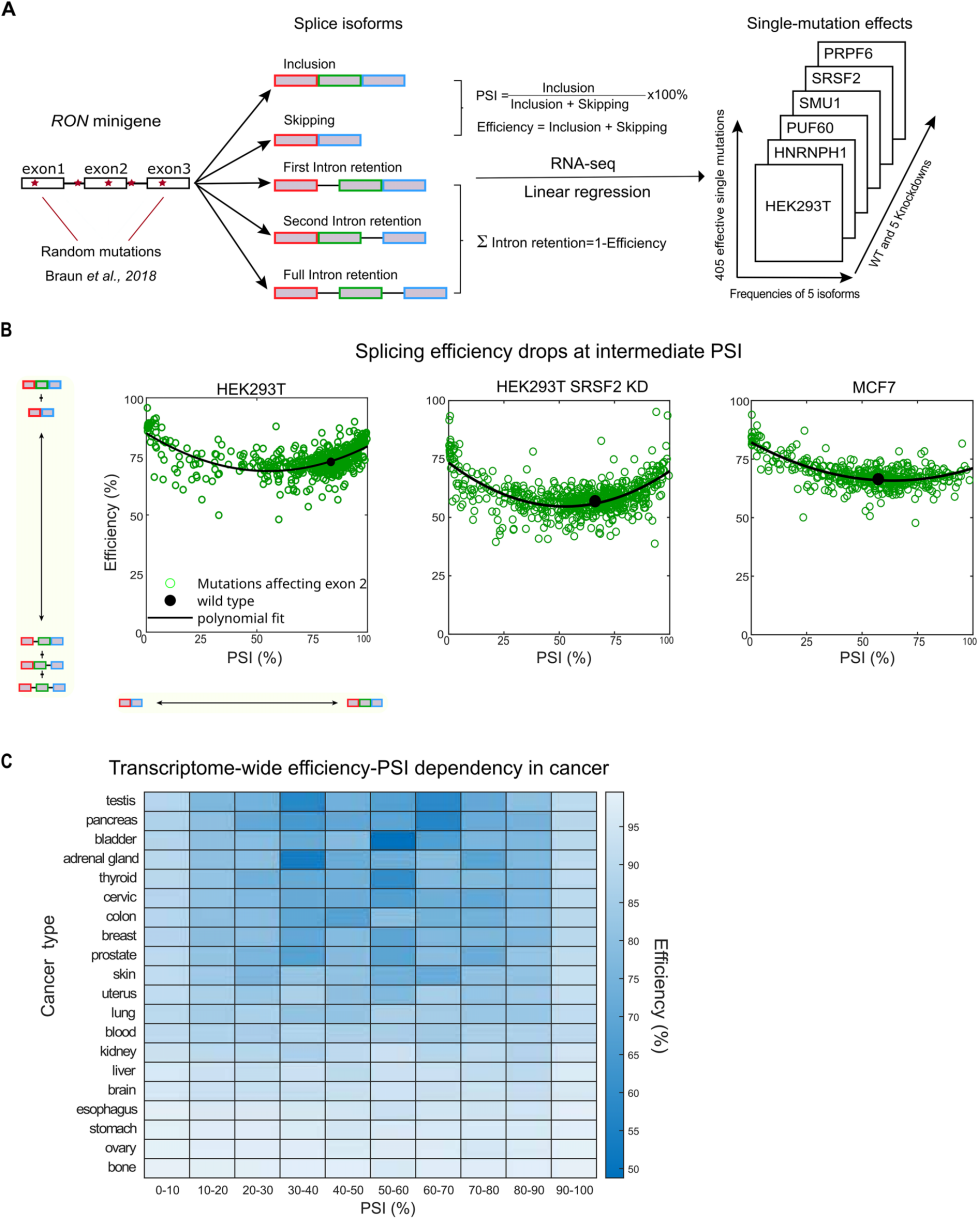
Splicing efficiency drops at intermediate alternative exon inclusion levels. (**A**) High-throughput mutagenesis data were used in this study. Braun *et al*. performed linear regression analysis on the random mutagenesis data of the *RON* minigene comprising exons 10–12 (here designated as exons 1–3) to quantify the single-mutation effects on abundance of five main splice isoforms, AE inclusion, AE skipping, first, second, and full intron retention [26]. Two metrics were used to characterize splicing outcomes for each point mutation: PSI quantifies the binary AE usage, and the splicing efficiency quantifies the summed frequencies of AE inclusion and skipping and is complementary to the sum of all intron retention isoforms. The analyzed data from HEK293T cells is a matrix containing the frequencies of five splice isoforms for 405 effective single point mutations, for control and five RBP knockdowns, namely HNRNPH1, PUF60, SMU1, SRSF2, and PRPF6 [26, 31]. (**B**) Splicing efficiency drops at intermediate PSI for various experimental conditions in the *RON* minigene. The plots depict PSI and efficiency across a spectrum of 274 single-point mutations specifically affecting exon 2(green dots). The black dot represents the WT *RON* minigene without mutations. The black line represents a polynomial fit for the AE mutations. Left: HEK293T cell line; middle: SRSF2 knockdown in HEK293T cells; right: MCF7 cells. (**C**) Splicing efficiency drops transcriptome-wide in cancer. Heatmap shows the median efficiency at different bins of PSIs in 20 cancers from different tissues, where the data were acquired from MAJIQlopedia [32]. The cancer datasets were from TCGA [40], TARGET [41], BEAT-AML [42], and CBTN [43].

To investigate the interplay between multiple splicing events, we made use of our previously published data from a high-throughput mutagenesis screen of minigenes harboring *RON* exons 10–12 [26, 31]. The pre-mRNA of the *RON* minigene is alternatively spliced into five major isoforms – AE inclusion, AE skipping, first intron retention (IR1), second intron retention (IR2) and full intron retention (fullIR), and we had identified 405 single point mutations (out of 1800 mutations in total) that significantly change the abundance of these isoforms (frequency change of at least one isoform is larger than 10%) [26, 31]. We characterize the interdependency of AE and IR events using two metrics, the percent spliced-in (PSI) for the AE decision and the efficiency for intron removal (Fig. 1A). PSI is defined as *PSI* = 100% × *f*_inclusion_ / (*f*_inclusion_ + *f*_skipping_), where *f*_inclusion_ and *f*_skipping_ represent frequencies of AE inclusion and skipping isoforms, respectively. The PSI quantifies the specificity of the binary AE decision, ranging from full skipping (PSI = 0%) to full inclusion (PSI = 100%). The efficiency of intron removal, defined as *efficiency* = *f*_inclusion_ + *f*_skipping_, measures the overall occurrence of active splicing and it is complementary to the total level of IR, i.e. *efficiency *= *f*_inclusion_ + *f*_skipping_ = 100% - (*f*_IR1_ + *f*_IR2_+ *f*_fullIR_).

We observed two modulation modes of IR and AE events: the change in IR level can be either insensitive or coupled to the AE decision. As reported previously [31], point mutations in the outer constitutive exons of the *RON* minigene promote the retention of the first or second introns (low efficiency), while having little or mild effect on PSI (Supplementary Fig. S1A, blue and red circles). In contrast, point mutations located in the AE not only effectively affect the AE inclusion/skipping, but also modulate the retention of both flanking introns, leading to the accumulation of the fullIR isoform (Fig. 1B, green circles; Supplementary Fig. S1A, green circles). Specifically, we observed a drop to ∼ 65% efficiency for AE mutations, establishing intermediate PSI levels when compared to the much higher splicing efficiencies (90%) observed for mutations inducing extreme PSIs (0 or 100%). To exclude that the splicing efficiency drop at intermediate PSIs is a *RON*-specific phenomenon, we analyzed the splicing outcomes of another minigene reporter containing *CD19* exons 1–3, subjected to a large-scale mutagenesis screen very similar to that for *RON* [27]. Interestingly, we observed the same pattern of PSI-efficiency dependency upon mutations affecting the AE (exon 2; Supplementary Fig. S2; Materials and methods). Taken together, these data suggest that in addition to independent modulation, IR can be regulated coordinately with AE decision, and this type of IR regulation is largely unexplored.

To further test whether this AE-IR coordination is controlled by *trans*-regulators, we analyzed *RON* mutagenesis data in different cellular contexts, including siRNA-mediated knockdown (KD) of several RBPs (HNRNPH1, PUF60, SMU1, SRSF2, and PRPF6; Fig. 1A) and using another cell type (MCF7 cells; Materials and methods). The non-monotonic dependency of splicing efficiency on PSI was indeed observed in all data sets (Fig. 1B and Supplementary Fig. S1A). Interestingly, the efficiency drop is quantitatively different across conditions. Some KDs show a dependency curve similar to that in unperturbed HEK293T cells, though individual minigenes (e.g. the unmutated WT minigene) shift in the PSI-efficiency space (HNRNPH1 and PUF60). In contrast, others (SRSF2, SMU1, and PRPF6) show a more pronounced efficiency drop at intermediate PSIs (Supplementary Fig. S1A). Thus, the splicing efficiency drop is an intrinsic property of AE regulation that is subject to regulation by *trans-*acting factors.

To explore beyond minigene reporters, we analyzed transcriptome-wide RNA-seq data of alternative splicing subjected to systematic knockdowns of 305 splicing factors/RBPs [10]. We extracted all exon skipping events associated with immediately upstream or downstream intron retention events, yielding a total of 24 512 AE-IR modules. For quantification, we calculated the average splicing efficiency of all exons within 10 PSI bins ranging from near-complete skipping (PSI 0–10%) to near-complete inclusion (90–100%). We indeed observed a marked drop in splicing efficiency for AE at intermediate PSI values on a global scale (i.e. across all conditions and exon skipping events) as well as at the individual event level (Supplementary Fig. S3; Materials and methods). In support of active regulation by trans-acting factors, RBP knockdowns not only perturbed PSI and splicing efficiency individually but also caused coordinated changes in both outcomes (Supplementary Fig. S3B; Materials and methods).

To assess whether the efficiency-PSI coordination is also observed in other genome-wide RNA sequencing datasets, we extracted and analyzed splice junctions showing both AE and IR events from 20 human tissues in normal and malignant conditions assembled in MAJIQlopedia [32] (Materials and methods; Supplementary Table S1). In line with previous observations, the average splicing efficiency drops at intermediate PSIs in all 20 cancer types (Fig. 1C) as well as their healthy counterparts (Supplementary Fig. S4). Interestingly, the amplitude of the efficiency drop varies across cancers, indicating that the AE-IR coordination is tissue- and cancer-type dependent (Fig. 1C and Supplementary Fig. S4). Notably, very similar results were obtained when performing the above-mentioned cancer tissue and *RON* minigene analyses using different sequencing read coverage cutoffs for each alternative splicing event and minigene variant, respectively (Supplementary Fig. S4; Supplementary Fig. S1B). This suggests that the observed PSI-efficiency dependency does not arise from sequencing biases but is a robust biological phenomenon.

Taken together, we found that the AE regulation affects coordinately the retention of its flanking introns, where balanced AE inclusion and skipping is associated with an increase in the percentage of non-spliced IR products. This phenomenon was not only observed in the minigene reporters (e.g. *RON* and *CD19*) but also transcriptome-wide in various genetic and cellular contexts. This raises questions about the molecular mechanism underlying this behavior.

### Two-step exon definition explains interdependence of AE and IR events

**Figure 2.**
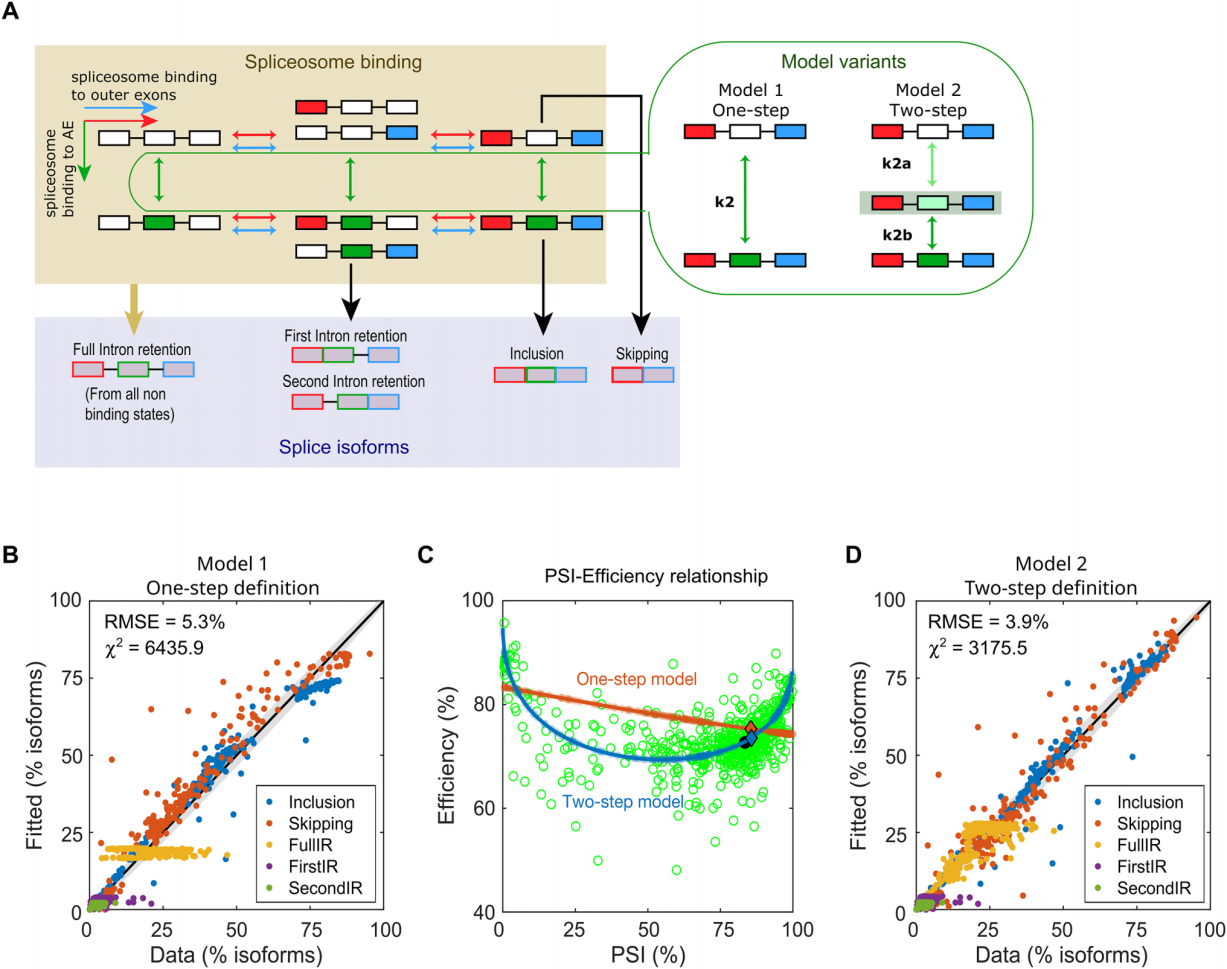
The two-step AE definition model explains the splicing efficiency drop at intermediate PSI levels. (**A**) Schemes of one-step and two-step AE definition model variants. The illustration inside the brown shading depicts the independent definition of the three exons in the minigene by spliceosome binding following the exon definition mechanism. Empty and filled boxes represent undefined and defined exons, respectively. The two model variants differ only in the AE definition mechanism, with one or two rate-limiting steps (green bubble and arrows), while the definition of the two outer exons follows the same one-step process (blue and red arrows). Exon definition states that determine splicing outcomes (blue shading): pre-mRNAs with all three exons defined give rise to the AE inclusion isoform, and those with only AE undefined skip the AE in the final product. If the first and the last exon are left undefined, first and second intron retention isoforms will be generated, respectively. All spliceosome binding states can be converted to the full intron retention isoform in both models. (**B**) The one-step model fails to fully describe the single-mutation effects on *RON* splicing. The one-step model was fitted to the frequencies of all five *RON* isoforms for 405 effective single mutations (274 mutations affecting the AE were shown here). Black lines: the diagonal line representing perfect data-model agreement; shade: isoform variability predicted by an error model estimated from the data (Materials and methods). RMSE: root of mean-squared error; χ^2^: weighted least squares. (**C**) Coordinated AE-IR regulation is described by the two-step model and not the one-step model. The data in green circles represent the mutations in the AE of the *RON* minigene, and the lines are simulations using the best fits of the one-step (red) and two-step (blue) models for varying AE recognition, respectively. Black dot: WT. (**D**) The two-step model faithfully captures the single-mutation data. The two-step model was fitted to the frequencies of all five *RON* isoforms for 405 effective single mutations. Same representation as in B.

To understand the mechanism of the interplay between AE and IR events, we developed quantitative mechanistic models and statistically tested their abilities to faithfully capture the data of point-mutation effects on *RON* splicing. Our previous work showed that the *RON* minigene, despite harboring short introns (87 and 80 nt), undergoes splicing preferentially via exon definition rather than intron definition [31]. Based on this finding, we studied an AE-definition model of *RON* splicing, in which the AE (exon 11) of the minigene is defined via cooperative binding of spliceosomal U1 and U2 snRNPs and subsequent formation of a cross-exon complex (Fig. 2A). The spliceosome assembly across the AE is therefore described as a one-step reversible association-dissociation process (Fig. 2A, one-step model in the green bubble). Note that the first and last exons of the minigene construct lack the upstream and downstream intronic sequences, respectively, and may thus be recognized by an intron definition mechanism unless the mRNA cap or the polyadenylation signal aids in the formation of cross-exon complexes [54, 55]. However, from a mathematical point of view, intron definition and exon definition are equivalent in describing the spliceosome recruitment to these constitutive exons, as both can be modeled by a single, reversible binding step, irrespective of the specific molecular mechanism of spliceosome assembly.

Assuming independent spliceosome binding of the three exons, we obtained 8 binding states and reasoned that these states dictate splicing outcomes. Specifically, a pre-mRNA with all three exons bound gives rise to the AE inclusion isoform, while the AE is skipped when spliceosomes only bind to the two outer exons. An unbound first or last exon, with the other two exons fully bound, leads to first or second intron retention, respectively. All binding states can be converted to the full IR isoform to reflect unproductive splicing or rapid nuclear export of unspliced pre-mRNA in the model (detailed model scheme see Supplementary Fig. S5A). As the spliceosome binding is determined by sequence context and RBPs, the model can be used to study the effects of genetic perturbations, e.g. mutations and RBP depletion, on splicing outcomes.

We translated the one-step AE definition model into ordinary differential equations (ODEs) and obtained the steady-state solution for the frequencies of the five splice isoforms (Materials and methods). To test whether this model can explain the single-mutation effects on *RON* splicing, we jointly fitted the simulated steady-state isoform frequencies to the corresponding measurements in HEK293T cells, taking into account data of the unmutated WT *RON* minigene and 405 single-mutants (standard *χ*^2^ fit employing an error model, Supplementary Fig. S6 and Materials and methods). In the model fitting, we assumed that each mutation only affects spliceosome binding to the nearest exon (Materials and methods). For instance, mutations within the AE or close to the borders of AE (-40 nt upstream of the 3′SS or + 40 nt downstream of the 5′SS) are assumed to solely affect the AE definition rate (*k*_2_ in Fig. 2A). In addition, even though no signatures of nonsense-mediated decay (NMD) were found in *RON* minigene isoforms via premature stop codon analysis, we introduced isoform-specific lifetimes as free model parameters that potentially regulate the isoform abundance (Supplementary Fig. S7; Materials and methods). While the isoform frequencies of the best fit seemed to follow the tendency of the data (Fig. 2B  Supplementary Fig S8A), the one-step AE definition model failed to capture the efficiency drop at intermediate PSIs upon AE mutations (Fig. 2C). Indeed, the model only allows for nearly independent regulation of AE and IR events by inner and outer exon mutations, respectively (Fig. 2C, the red line; Supplementary Fig S8B, red lines). Thus, the one-step AE definition model is intrinsically limited in describing the interplay between the two splicing decisions, even with optimized parameters.

To overcome this limitation, we extended the AE definition model. Recent studies reported that the spliceosome undergoes extensive energy-dependent conformational change before splicing catalysis [13–16], which may lead to multiple rate-limiting steps during exon definition. To incorporate this stepwise spliceosome assembly and maturation, we developed a parsimonious model in which the AE definition takes two consecutive steps with different rates (Fig. 2A, two-step model in the green bubble). The intermediate state between the two steps reflects immature or uncommitted spliceosome assembly that potentially hinders subsequent splicing catalysis and thus only yields the unspliced isoform, i.e. full IR. We fitted this model with the same *RON* point mutation data, assuming that U1 and/or U2 act in both steps of AE definition and thus a mutation within or nearby the AE affects the rates of both steps (Materials and methods). Notably, the two-step AE definition model outperformed the one-step model by faithfully recapitulating the effects of mutations in all three exons, as judged by the root mean square error (RMSE) and χ^2^ value (Fig. 2D; Supplementary Fig. S8). In particular, the best fit is a two-step model that quantitatively describes the splicing efficiency drop at intermediate AE inclusion level (Fig. 2C, blue arc). In conclusion, by iterative data-driven modeling, we showed that the two-step AE definition model reproduces the coordinated occurrence between AE and IR events, suggesting that multiple rate-limiting steps shape spliceosome decisions.

### Two-step exon recognition combines isoform switching with high splicing efficiency

**Figure 3.**
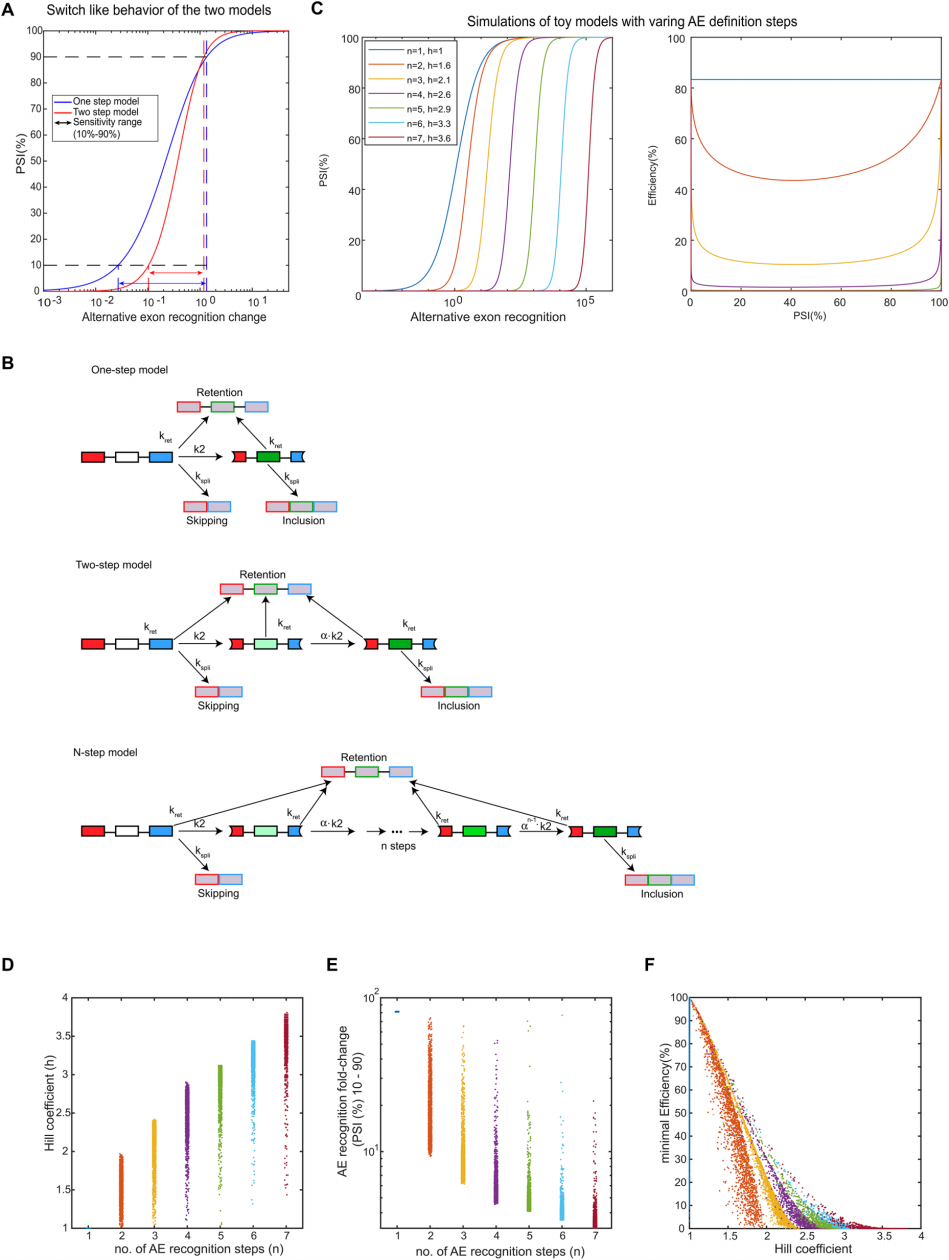
Two-step model balances a trade-off between sensitive switching and high efficiency in splicing regulation. (**A**) Response of PSI to changes in the AE definition rate simulated by the best-fit one-step (blue curve) and two-step (red curve) models from Figs. 2B and D. The simulation was performed by varying only the AE definition rates (*k*_2_ in the one-step model; proportional change in *k*_2a_ and *k*_2b_ in the two-step model) starting from the best-fit parameters. To switch the AE inclusion level (PSI) from 10% to 90% (horizontal dashed lines), a 50 (blue dashed lines) and 11 (red dashed lines) fold-change in the AE definition parameters is required in the one-step and two-step models, respectively. (**B**) Toy models describing one-step, two-step, and *n*-step AE recognition. The two outer exons were assumed to be strongly recognized (i.e. constitutive exons), and only AE recognition was modeled with different numbers of binding steps (from 1 to 7). For multi-step models (*n* ≥ 2), the recognition rates were assumed to change stepwise by the same fold-change factor (*a*) between consecutive steps, i.e. rate of the *i*th step $ = {{k}_2}{{\alpha }^{i - 1}}$, and *α*<<1 was assumed to reflect efficient proofreading. The fully defined and undefined AE states give rise to AE inclusion and skipping isoforms, respectively, with a common splicing rate *k*_spli_. Another alternative splicing decision possible in all recognition states is the retention of introns in the final product (with the rate *k*_ret_). (**C**) Multi-step AE definition strengthens the switch-like AE regulation at the cost of reducing splicing efficiency. Left panel: dose-response curves of PSI as a function of AE recognition strength (*k*_2_) were simulated for a range of intermediate AE recognition steps (from 1 to 7). The one-step model showed the weakest switch-like regulation (Hill coefficient *h* = 1), whereas multi-step models allowed for a sharper switch (Hill coefficient *h* ranging from 1.6 to 3.8) with increasing number of steps (from 2 to 7). Right panel: However, the same simulations showed that the splicing efficiency drops if the number of intermediate steps is larger than two, which reflects strong accumulation of unproductive splicing products. (**D–F**) Systematic parameter sampling confirms the trade-off between switch-like AE regulation and splicing efficiency for a large range of parameter values. All parameters of the toy models were randomly sampled (see Materials and methods), and for each simulation, the Hill coefficient and the minimal splicing efficiency for varying *k*_2_ were calculated as in (**C**). In line with the results in (**C**), the switch-like regulation (quantified by the Hill coefficient) increases (**D**), and the sensitive switching interval of AE recognition strength (causing 10% to 90% change in PSI) decreases (**E**) with the number of intermediate AE recognition steps. Furthermore, the trade-off between switch-like AE modulation and splicing efficiency still holds true (**F**). Same color code as in (**C**).

While the two-step exon recognition well explained the AE-IR dependency (Fig. 2), it is unclear whether and how this mechanism provides any functional significance. To investigate the potential benefit of the two-step regulation for alternative splicing, we performed numerical simulations in which we induced a shift from skipping to inclusion by varying the AE recognition parameters (from the best-fit values). In these simulations, we compared one- and two-step mechanisms with respect to the PSI response curve. Even though altered recognition of the AE induces a complete shift from skipping to inclusion in both models (PSI from 0 to 100%), the PSI responds more sharply in the two-step model (Hill coefficient *h* = 1.8) than in the one-step model (*h* = 1.1). Thus, in the two-step model, a relatively small change in AE recognition, due to mutations or other regulatory mechanisms, is sufficient to effectively switch splicing outcomes from skipping to inclusion. Specifically, an 11-fold change in the AE recognition parameter switches the PSI from 10% to 90% (Fig. 3A), whereas the one-step model requires a much larger change in AE recognition [50-fold) to achieve the same PSI switching. Therefore, the two-step AE definition enables a more efficient splicing switch.

Spliceosome assembly involves the recruitment of more than 100 proteins [56], immediately raising the question whether this process needs to be modeled using more than two rate-limiting steps. To address this question, we focused on the core module of AE definition and formulated toy model variants with different complexity by varying the number of intermediate recognition steps (one to seven) during the transition from an undefined to a fully defined AE (Fig. 3B). In all model variants, the first and the last species in the chain give rise to skipping and inclusion products, respectively, whereas no productive splicing, but only IR, is possible from the partially defined AE intermediates.

By simulations using the best-fit AE definition parameters, we observed almost the same Hill coefficients in the one- and two-step toy models as in the corresponding full models, confirming that the toy models well characterize AE switching from skipping to inclusion. For the model variants with three to seven AE definition steps, the simulated Hill coefficients range from 2.1 to 3.6, implying that a 6- to 3-fold change in AE recognition is required to switch the PSI from 10 to 90%, respectively (Fig. 3C, left). Compared to the difference in switch-like behavior between the one- and two-step models, these values of the three to seven-step models are close to those of the two-step model. However, increasing the number of AE recognition steps comes at a cost for splicing efficiency, as the IR levels drastically increase with the number of molecular steps (Fig. 3C, right). Thus, a trade-off appears to exist, in which a larger number of AE recognition steps leads to a gain in switch-like splicing behavior and, at the same time, to a loss in splicing efficiency.

To test this result in a more general context, we randomly sampled the kinetic parameters in all toy model variants and quantified the PSI response for each parameter set to estimate the range of Hill coefficients (Fig. 3D; Materials and methods). Indeed, the gain in isoform switching realized by one additional molecular step continuously decays with the increasing number of molecular steps (Fig. 3E). Hence, increasing the number of exon definition steps beyond two has a limited effect on switch-like PSI modulation and therefore the potency of splicing regulation for a given fold-change in AE recognition. Moreover, for models with more than two molecular steps, we observed a very strong accumulation of unspliced precursors at intermediate PSI (Fig. 3F). Thus, a higher number of intermediate steps leads to problems with splicing execution, slowing down the reaction and/or leading to the accumulation of products with terminal IR.

Taken together, we introduced model variants with different numbers of intermediate AE definition steps to explore the biological consequences of multi-step splicing and showed that two-step AE recognition meets a trade-off, combining sensitive isoform switching with high splicing efficiency. Models with a larger number of intermediate steps enable a strong AE inclusion switch, but they may severely compromise the production of functional isoforms (IR dominating). Thus, the two-step model is most consistent with the experimental data, suggesting that it serves as a reasonable simplification of multistep spliceosome assembly.

### Modulating individual AE definition steps by RBPs strengthens AE-IR dependency

Next, we sought to understand how *trans-*acting regulators (e.g. RBPs) control the coordinated AE-IR decisions. To reflect our *RON* mutagenesis data, in which RBP KD effects are assessed across the whole spectrum of mutations (see below and Supplementary Fig. S1), we simulated how the efficiency-PSI dependency shifts when an RBP controls molecular steps in the best-fit full model of two-step AE definition (Fig. 2D and Materials and methods).

**Figure 4.**
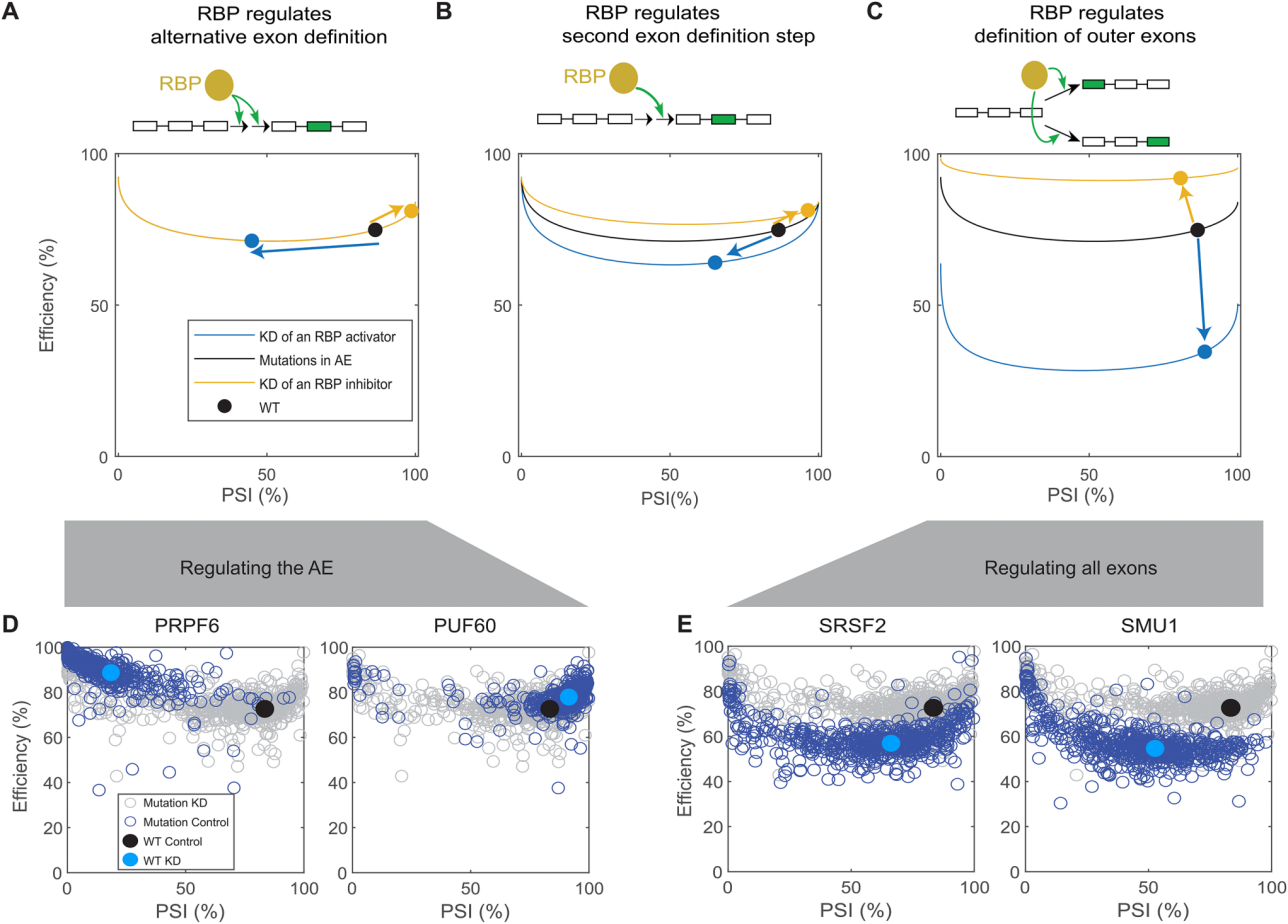
RBP KDs have qualitatively different effects on efficiency-PSI dependency. (**A**–**C**) Qualitative simulations were performed to mimic knockdowns of RBPs regulating different exon-definition steps in the two-step model. The parameters from the best-fit of the *RON* minigene (Fig. 2D) were altered according to the following regulation modes: RBPs can exclusively modulate AE by changing proportionally both AE definition rates (**A**) or only one of them (**B**). Alternatively, RPBs can also affect the definition of the two outer exons (**C**). The black dot and the black curve represent the efficiency-PSI dependencies in the unmutated WT control and simulated exon 2 mutants, respectively. The blue and yellow curves indicate corresponding shifts simulated for a KD of RBPs acting as an activator (blue) or a repressor (yellow). The three curves coincide in (**A**). (**D** and **E**) PSI-efficiency shifts observed in RBP KD data for the *RON* minigene resemble the simulations in (A–C). (**D**) Compared to scrambled KD control (grey circles: mutants; black dot: WT), the PRPF6 and PUF60 KDs (blue circles and light blue dots) mainly shift the position of unmutated WT minigenes (dots), while leaving the trend of mutated minigenes unaffected (circles). This behavior resembles exclusive AE regulation simulation in (**A**). (**E**) SRSF2 and SMU1 KDs globally change the efficiency-PSI relation for both unmutated and mutant minigenes, mimicking a combined modulation on both AE and outer exons (combination of simulation scenarios in B and C). Same representation as in (**D**).

RBPs regulating AE recognition can show two distinct modes, depending on whether they perturb both steps jointly (Fig. 4A) or one of them individually (Fig. 4B). In the joint modulation mode, an RBP alters both AE definition rates proportionally without changing their ratio. KD of this RBP is predicted to coordinately shift the efficiencies and PSIs of mutated and unmutated *RON* minigenes (dots in Fig. 4A) along the same efficiency-PSI dependency curve (overlayed curves in Fig. 4A). Therefore, the joint modulation on AE definition steps does not change the overall AE-IR coordination. Alternatively, an RBP may regulate only one of the two AE definition steps, e.g. modulating only the second step (Fig. 4B). In contrast to the joint modulation mode, regulation of the individual AE definition step reshapes how strongly the IR products accumulate at intermediate PSI values (distinct curves in Fig. 4B). For example, slowing down the second AE definition step (KD of an activator) reduces the minimal splicing efficiency while leaving high efficiencies at extreme PSIs unaffected, thereby strengthening the efficiency-PSI dependency (blue curve in Fig. 4B). RBPs may also regulate the definition of the outer (constitutive) exons. In our simulations, the KD of such an RBP causes an overall splicing efficiency shift (vertically along the efficiency axis) at all PSI levels, without a pronounced change in PSIs (horizontal shift) for both WT and mutant *RON* minigenes (Fig. 4C). Beyond these basic types of RBP-dependent regulation, our model also allows us to simulate more complex scenarios, in which both alternative and constitutive exon recognition are regulated upon KD (see below).

Interestingly, in the *RON* RBP KD data in HEK293T cells, we observed altered efficiency-PSI dependencies that resembled the above model predictions. First, the PRPF6 and PUF60 KDs leave the overall efficiency-PSI curve unaffected, along which the WT and mutants reallocating their positions (Fig. 4D). This is in line with the joint modulation of both AE definition steps (Fig. 4A). Second, SRSF2 and SMU1 KDs induce changes in both the lowest efficiency at intermediate PSI and efficiencies at border PSIs, indicating a combined regulation of the individual AE definition step and outer-exon definition (i.e. Fig. 4E resembles a combination of simulated effects in Fig. 4B and C). These observations suggest that quantitative changes in the efficiency-PSI relationship can reveal which splicing step(s) an RBP regulates and whether it acts as an activator or inhibitor.

**Figure 5.**
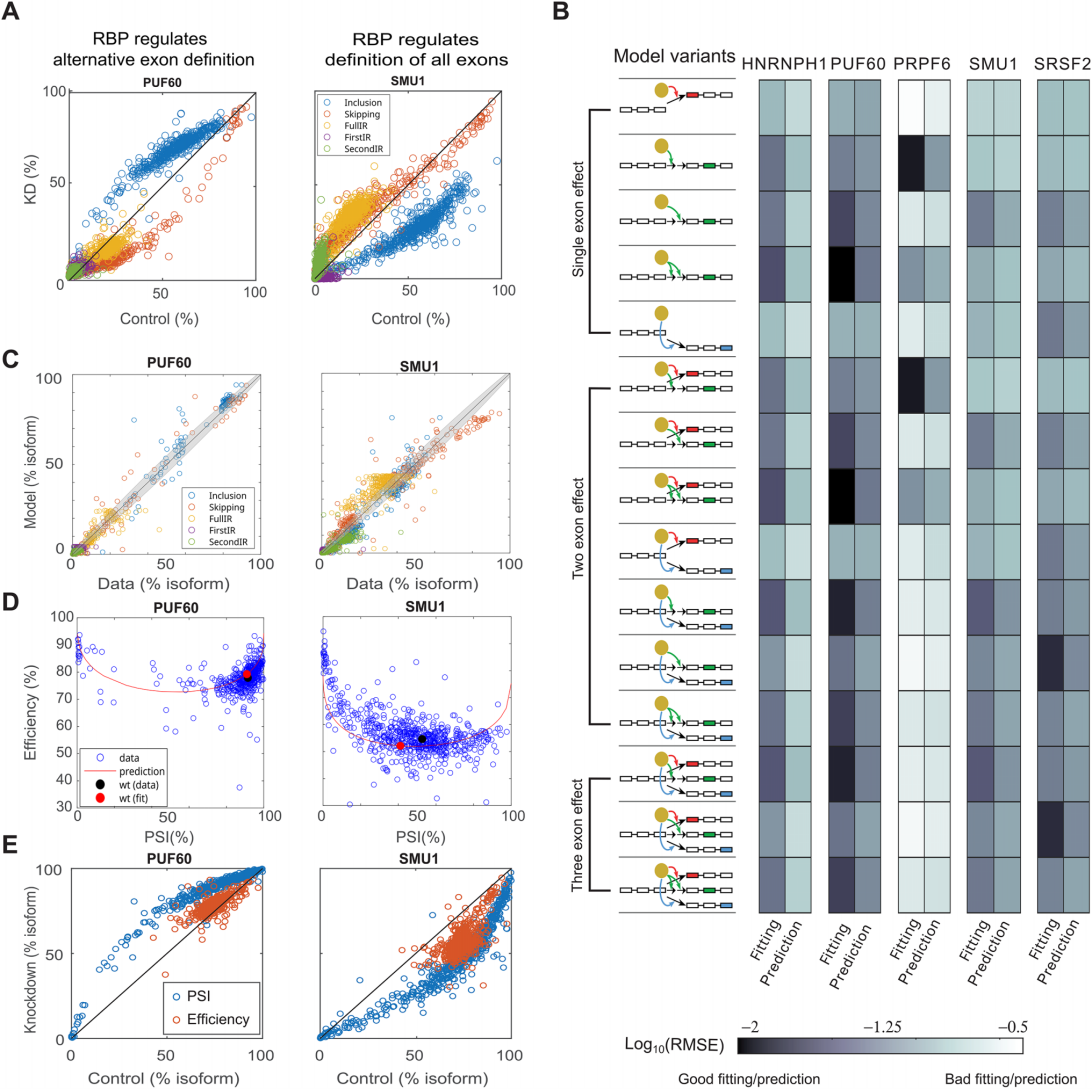
Systematic model selection reveals distinct regulatory roles of RBPs. (**A**) Data of the frequencies of the five *RON* isoforms in KD scrambled control vs. RBP KD conditions in the 405 splicing-effective single mutants (each circle representing a single mutation). PUF60 KD mainly changes the AE inclusion (blue) and skipping (red) isoforms (left panel), whereas SMU1 KD additionally shifts IR isoforms (right panel). (**B**) Computational model selection identifies the regulation modes of distinct RBPs. 15 model variants were generated according to all possible combinations of RBP modulation on each exon-definition step, as indicated by the schemes on the left (RBP: yellow dot; RBP-mediated regulation: arrows). For each RBP KD, the unmutated WT minigene was fitted by all 15 model variants, starting from the best-fit model (Fig. 2D) and re-fitting only the assumed RBP-regulated parameters. The mutant data (274 single AE mutations) were used for cross-validation to test the predictive power of each model variant (see Materials and methods for details). Model variants are compared with respect to the fitted and the predicted RMSE values (Log_10_RMSE for color code). (**C**) Best models combining good fit and high predictive power selected in (**B**) successfully capture the frequencies of all five splice isoforms in 274 single AE mutants and the unmutated minigene for PUF60 (left) and SMU1 (right) KDs shown as examples. Color code: five isoforms. (**D**) Coordinated efficiency-PSI regulation is reproduced by the best-fit models in (**C**). Black and red filled circles represent measured and fitted unmutated WT minigene, respectively. Blue open circles and red curves are the measured data points and model-predicted curves for single point mutants in exon 2, respectively. (**E**) PSI (blue) and efficiency (orange) jointly describe the regulatory roles of RBPs when comparing KD scrambled control (x) and RBP-targeted KD (y) for WT and all single mutants (circles). PSI alone can only identify the directionality of an RBP affecting the AE, e.g. PUF60 as a repressor (left) and SMU1 as an activator (right). Additional quantification on efficiency adds another dimension for identifying more detailed modes of regulation (PUF60 KD: no efficiency change; SMU1 KD: efficiency reduction).

Further inspection of the full data, considering all five splice isoforms, supported our hypothesis that coordinated shifts of splicing outcomes can be used to distinguish modes of RBP regulation. For example, the KD of PUF60, PRPF6, or HNRNPH1 mainly affects inclusion and skipping isoforms (Fig. 5A, left panel; Supplementary Fig. S9A), whereas SMU1 or SRSF2 KDs lead to an accumulation of full intron retention at the expense of inclusion isoforms (Fig. 5A, right panel; Supplementary Fig. S9A). To identify the RBPs’ regulatory roles in a more quantitative and systematic way, we built a family of models (15 model variants in total) in which an RBP can modulate all possible combinations of exon-definition steps and confronted all model variants with isoform data for each RBP KD (Fig. 5B). To parameterize the RBP KD effects, we introduced a single parameter in each model variant that quantifies the fold change in the RBP-regulated exon definition rate(s) upon RBP KD. If an RBP modulates multiple steps, we assume that the rates of these steps share the same fold change (details see Materials and methods).

Next, we selected the best model(s) for each RBP KD by ranking both the goodness-of-fit and predictive power of the model variants, quantified by RMSE. We first fitted each model variant to the frequencies of five isoforms of unmutated *RON* minigene upon RBP KD (columns labeled fitting in Fig. 5B; Supplementary Table S4). Then, we cross-validated the fitted RBP KD effects by predicting the isoform frequencies for 274 effective *RON* single mutants (specifically affecting AE) in the KD condition. Here, it is assumed that the single-mutation effects on the exon definition are the same for control and the RBP KD condition in HEK293T cells (Materials and methods; columns labeled prediction in Fig. 5B). This unbiased systematic model selection identified three regulation modes for the tested RBPs: exclusive modulation on both (HNRNPH1 and PUF60) and single (PRPF6) AE definition steps, as well as combined modulations on outer exons and an individual AE definition step (SMU1 and SRSF2). The fitting and prediction from the best models agreed well with the KD data for all tested RBPs (PUF60 and SMU1 KDs in Fig. 5C; others in Supplementary Fig. S9A). Consistent conclusions were obtained when using both RMSE and *χ*^2^ value as model discrimination metrics (Supplementary Fig. S9B, C). The model also successfully predicted the characteristic shifts in efficiency-PSI dependency for distinct regulator types (Fig. 5D compared with Fig. 4A–C).

Importantly, the consideration of five splice isoforms in our model fitting to RBP KD data leads to more detailed insights into the RBPs' mechanism-of-action compared to conventional PSI analysis: the change in PSI solely informs on the directionality of the regulation, classifying RBPs into activators (SMU1) or inhibitors (PUF60) of AE recognition (Fig. 5E, blue dots). Complementing this by a quantification of the splicing efficiency (or IR) reveals whether the outer constitutive exons are additionally regulated (SMU1) or not (PUF60), thereby better distinguishing the regulatory modes of different RBPs (Fig. 5E, red dots). At the level of five individual splice isoforms, the RBPs' mechanisms of action can be resolved further (Fig. 5A), especially when combined with rigorous model selection (Fig. 5B).

Taken together, we found that the dependency of splicing outcomes can be differentially modulated by RBPs acting in distinct exon-definition steps, which provides a basis for quantitatively discriminating the regulatory roles of RBPs in alternative splicing regulation.

### Two-step exon recognition is commonly perturbed in cancers

**Figure 6.**
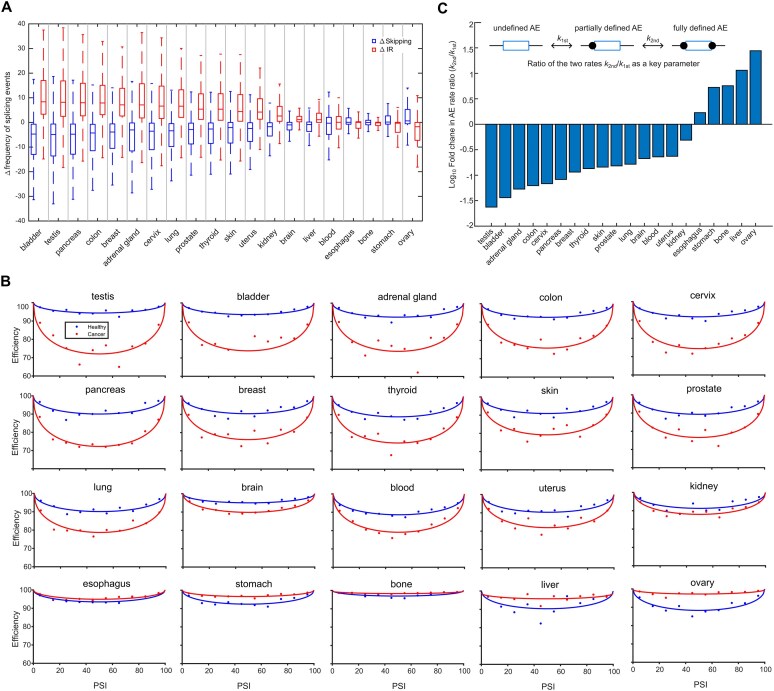
AE definition steps are differentially perturbed in cancers. (**A**) IR accumulates in cancer. MAJIQ-processed transcriptome-wide RNA-seq data from 20 human tissues are shown as a boxplot of ΔAE skipping and ΔIR values, i.e. differences between cancerous and normal tissue, across > 400 000 splicing events combining AE and IR decisions (the quantification of AE skipping and IR events see Materials and methods). IR (red) increases and AE skipping (blue) declines in most cancer types. Data source: normal tissue data are from GTEx [33], BLUEPRINT [34], Leucegene [35], and Ranzani *et al*. [37]; cancer data from TCGA [40], TARGET [41], BEAT-AML [42], and CBTN [43]. Boxplot shows median (horizontal line), interquartile range (box), and non-outlier extreme values (whiskers). (**B**) A core two-step AE definition model explains the tissue-dependent efficiency-PSI relation shift in cancers. The median splicing efficiency across all splicing events combining AE and IR decisions in 10 PSI bins drops at intermediate PSIs in 15 out of 20 tissues (first three rows), whereas five other tissues showed the opposite effect (last row). To quantify this tissue-dependent transcriptome-wide efficiency-PSI relation, a simplified two-step model was derived from the full model for *RON* minigene (Materials and methods). This core model was fitted to the data in the 20 normal (blue) and cancerous (red) tissues. (**C**) The first and second AE definition steps are strongly modulated in cancers compared to normal tissues. The parameter inference from the best fits in (**B**) is shown as fold-change in the relative rate (or rate ratio) of the two AE definition steps (*k*_2nd_/*k*_1st_) of 20 cancer types over the corresponding normal counterparts. Increased IR compared to normal tissue in 15 out of 20 cancer types is explained by an accelerated early or/and slowed late AE definition step (a decrease in the ratio between the rates of second and first step), while the other 5 cancers showed the opposite (an increase in the rate ratio).

The PSI-dependent IR accumulation is observed not only in *RON* but also transcriptome-wide across human tissues and, remarkably, it is altered in many cancers (Fig. 1C and Supplementary Fig. S4). The frequency of IR events relative to productive splicing events is known to increase in most cancer types [9]. We confirmed that this result also holds in complex splicing events combining IR and AE, where the change of IR levels (median value up to 9%) is generally larger than that of AE skipping (median value < 5%) in each cancer type (Fig. 6A).

To explain the transcriptome-wide AE-IR relation in cancers, we analyzed a core model of two-step AE definition simplified from the full model for the *RON* minigene (Supplementary Fig. S10A; Materials and methods). Since no full isoform information is available in transcriptome-wide data, we reduced the number of parameters in this core model to a minimum. For instance, we assumed that all reversible exon definition steps reach equilibrium rapidly and that the two outer exons share the same definition rate, which results in only two independent parameters for the core model (Materials and methods).

To identify the regulatory mode(s) of the IR accumulation in cancers, we fit the core model separately to experimental data from both normal and cancer samples in each tissue, using the measured median splicing efficiencies for all binned PSI intervals (Materials and methods). The core model well captured the efficiency-PSI dependency in all 20 normal and cancer tissues (Fig. 6B). The most varied model parameter (from the best fit) between cancer and normal tissues is the ratio of the two AE definition rates, suggesting that either the first or the second AE definition step is preferentially perturbed in cancers (Fig. 6C and Supplementary Fig. S10B). Interestingly, this AE modulation is fine-tuned to exhibit distinct IR accumulation patterns in different tissues (Fig. 6C). 15 out of 20 tissues showed a slower late or a faster early AE definition step in cancer, which leads to a stronger splicing efficiency drop at intermediate PSI values (Fig. 4). The remaining 5 tissues showed the opposite effect. When the change in rate ratio is mild (2- to 10-fold), the IR (or efficiency) shifts almost uniformly across different AE levels (breast, thyroid, skin, prostate, lung, brain, blood, and uterus with an increase in IR; stomach and bone with a decrease in IR). However, in some tissues, the IR preferentially accumulates or declines at intermediate AE inclusion levels (testis, bladder, adrenal gland, colon, cervix, and pancreas with IR increase; liver and ovary with IR decrease). The model explains this PSI level-dependent IR by a greater change in the rate ratio of the two AE definition steps in cancer compared to normal samples (Figs. 6B and C).

Taken together, we show that the AE-dependent IR accumulation is a global trend at the transcriptome-wide level, and its perturbation in cancers can be explained by the modulation of only a small number of the rate-limiting steps in AE definition, implying a potential direction to search for the common factors responsible for splicing dysregulation in cancers.

## Discussion

Alternative splicing diversifies the human transcriptome by producing different RNA and protein isoforms from the same gene. These complex splicing decisions are carried out by a common molecular machinery, the spliceosome [10, 34]. How general spliceosome factors determine specific splicing fates is still a puzzling question in the field [10, 35]. In this work, we provide evidence that stepwise spliceosome maturation allows for the coordination of different splicing decisions.

We showed that AE and retention of its flanking introns are coordinately modulated, where IR occurs preferentially at intermediate AE inclusion levels. This AE-IR dependency was observed in large-scale mutagenesis studies of *RON* and *CD19* minigenes, and in transcriptome-wide data, in which either *trans*-regulators were extensively perturbed or normal and malignant tissues were compared. Importantly, these data indicate that the splicing efficiency is not only modulated by the alteration of overall spliceosome activity in the cell, but that IR is additionally coupled to the skipping/inclusion ratio when mutations modulate the strengths of (*RON)* AE recognition. For a different mode of regulation induced by mutations weakening the outer constitutive exons of the *RON* minigene, we observed that the change in IR level can be uncoupled from the AE decision (Supplementary Fig. S1). However, the IR-AE coupling was unexpected and may serve as an important mechanism of intron retention in cells, since AE recognition is subject to strong regulation across cell types and in cancer development.

To explain this phenomenon, we implemented mechanistic models describing stepwise spliceosome maturation. We built our models based on the splicing outcomes of the three-exon *RON* minigene, which leads to the production of five isoforms, AE inclusion and skipping, as well as three intron retention isoforms. The *RON* minigene is an artificial system that cannot fully recapitulate the complex splicing events occurring in the endogenous pre-mRNA, yet it harbors intact *cis*-regulatory elements of the AE (exon 11) and thus provides a reliable basis for dissecting the regulation of local AE-IR decisions. To study how the spliceosome controls these splicing decisions, we tested two model variants: the one-step and two-step AE definition models (Fig. 2A). The kinetic steps are mathematical abstractions quantifying multiple rate-limiting processes during early recognition and commitment of the AE, prior to splicing catalysis. They are not intended to map specific molecular events, but to capture the overall effect and sequential nature of early and late steps in spliceosome assembly (e.g. U1/U2 binding, stable splice-site communication via U4/U6.U5 tri-snRNP assembly). While the spliceosome maturation was often characterized as the behavior of the cross-intron complex, recent studies revealed that the formation of the cross-exon spliceosome is highly similar to that of the cross-intron complex, including U1-U2, U4/U6.U5 tri-snRNP assembly and their compositional remodeling [14–16]. This supports our model assumption that multiple rate-limiting steps may exist in the exon definition process.

Both models explained the splicing regulation by mutations in outer exons, and they even captured a weak negative correlation between efficiency and PSI (Supplementary Figs S1 and S8). In our models, the pre-mRNAs were first spliced into the first or second IR intermediates and then are either further spliced into the AE inclusion isoform or exported from the nucleus as mature IR isoforms (Supplementary Fig. S5). A mutation weakening the recognition of either outer exon consistently represses the skipping isoform. However, despite the mutation in one outer exon, the definition of the other outer exon can still efficiently proceed, thereby promoting partial IR and, via the stepwise intron removal, the AE inclusion isoform, capturing the observed weak negative correlation between PSI and efficiency for mutations in the outer exons.

For mutations in the AE or close to the AE borders, our analysis showed that the one-step model only allows for nearly independent regulation on AE and IR decisions (Fig. 2C). In contrast, the two-step model by nature enables IR product accumulation (or drop in splicing efficiency) at intermediate AE inclusion levels, therefore better accounting for the data of all five *RON* isoforms (Fig. 2D). At its core, the two-step model provides a kinetic-proofreading-like mechanism that disfavors a splicing decision for AEs with intermediate recognition levels, and instead leads to spliceosome rejection, intron retention, and potentially non-functional products [57]. In the literature, it has been shown that stalled pre-mRNA-spliceosome complexes can indeed undergo nuclear exit to the cytoplasm [58, 59], where they are engaged in active translation or degraded [57]. Alternatively, stalled spliceosome intermediates can be removed by nuclear degradation pathways [60–62]. Other mRNA degradation pathways rely on the detection of premature stop codons (NMD) or codon optimality [63, 64]. Thus, another possible proofreading mechanism is the preferential degradation of certain splicing products or/and intermediate spliceosome/pre-mRNA assembly states. Without assuming specific pathways of degradation, our models explicitly incorporate distinct lifetimes of *RON* minigene isoforms, but this mechanism alone (e.g. in the one-step AE definition model) was not sufficient to capture the coordinated PSI-efficiency relation observed in the data (Fig. 2C). Further arguing against a strong impact of degradation, our *in silico* screening for premature stop codons in *RON* minigene variants did not reveal any indications for isoform- and/or mutation-specific nonsense-mediated decay (Supplementary Fig. S7).

Alternatively, it is possible that intermediate-PSI exons with a high rate of unsuccessful splicing attempts show excessive degradation of intermediate states, alongside the accumulation of intron-containing precursors not engaged by the spliceosome (Supplementary Fig. S11A–C). Interestingly, this alternative model is very similar to our AE definition model, as it involves a degradation-mediated quality control over AE decisions along the multistep chain of spliceosome assembly (Supplementary Fig. S11D). Integrating the mechanism of preferential degradation of spliceosome assembly intermediates into our two-step AE definition model generated little difference in describing the efficiency drop at intermediate PSIs (Supplementary Fig. S11E). This suggests that the precise implementation of kinetic proofreading does not seem to be important for the qualitative behavior of PSI-efficiency dependency. Direct measurements of transcript stability are required to further test the proposed mechanisms.

AE proofreading enables a splicing switch, in which moderate changes in AE recognition are sufficient to induce pronounced transition from complete skipping (PSI = 0%) to complete AE inclusion (PSI = 100%) (Fig. 3A). Kinetic proofreading is widely observed in many biological processes with high fidelity (e.g. T cell receptor recognizing antigens), and it is often featured by multiple proofreading steps [65]. The spliceosome assembly involves the recruitment of more than 100 proteins, so more than two rate-limiting steps can easily be imagined. For instance, spliceosomal proofreading has been reported to occur at the level of U2 assembly [66–68], before the first catalytic step [69], or before the second catalytic step [57]. By analyzing model variants with different numbers of intermediate AE definition steps, we showed that the two-step model arrives at a trade-off combining AE inclusion/skipping, switching, and efficient splicing. Models with a larger number of intermediate steps enable a highly sigmoidal AE inclusion switch, but they may severely compromise the production of functional isoforms (IR dominating). Thus, the two-step model studied in the paper is most consistent with the experimental data, suggesting that it serves as a reasonable simplification of multistep spliceosome assembly.

At the single-cell level, this splicing switch allows for two modes of isoform expression regulation, digital (all-or-none) or analogue (gradual) modulation. In the digital mode, a cell exclusively produces either the AE inclusion or skipping isoform. The average PSI of AE in a cell population or a tissue depends on the fraction of cells expressing inclusion or skipping isoforms, respectively. In contrast, the gradual modulation allows simultaneous production of both AE inclusion and skipping isoforms in every single cell, and the population-level PSI is regulated via changing the isoform distribution within individual cells. To analyze which strategy has evolved in splicing regulation, we performed stochastic simulation [70] of the multi-step model of *RON* using the fitted parameters (Fig. 2) and found a gradual shift from skipping to inclusion when varying the strength of AE recognition (Supplementary Fig. S12). Our finding of gradual splicing decision-making in single cells is in line with our previous modeling work [71] and a single-molecule RNA-FISH study, which reported a unimodal distribution of alternative splicing outcomes across cells [72]. For single-cell RNA-seq studies, both unimodal and bimodal splicing outcomes have been described [73–75], but it remains to be seen how much these findings are influenced by limited read coverage, which may lead to apparently bimodal distributions at low read counts.

The degree of PSI-dependent IR accumulation is subject to regulation, as shown by the varied amplitude of splicing efficiency drop in *RON* minigene upon RBP KDs (Fig. 4). Qualitative simulations and systematic model discrimination revealed that the efficiency drop at intermediate PSIs is most sensitive to modulation on one of the two AE definition steps. The drop is most pronounced when the late step is much slower than the early step during spliceosome assembly and maturation, as this leads to stronger ‘proofreading’ of the AE. More specifically, it is the ratio of the first and second definition rates rather than their absolute values that determines the amplitude of the efficiency drop. Therefore, a strong efficiency decline can result from an RBP KD that either speeds up the first step, e.g. SMU1 KD (Fig. 5B), or slows down the second step, e.g. SRSF2 KD (Fig. 5B). In addition, both SMU1 and SRSF2 were also inferred to regulate the two outer exons, but this type of regulation generally lowers the splicing efficiency across all PSI values. Interestingly, a recent study conducting systematic KDs of ∼ 300 spliceosome components and RBPs reported that KDs of factors involved in the late stage of the spliceosome cycle preferentially led to elevated IR accumulation transcriptome-wide, while early-stage factor KDs showed the opposite effect [10]. This IR regulation directionality agrees with our model prediction concerning early and late AE recognition steps, suggesting that the differential regulation on the rate-limiting steps in the spliceosome assembly cycle might be a general mechanism for coordinating splicing decisions.

Indeed, we observed PSI-dependent IR accumulation transcriptome-wide in 20 normal and malignant human tissues and found that the splicing efficiency at intermediate PSIs is altered in most cancers. Interestingly, the majority of the cancer types (15 out of 20) showed an amplified splicing-efficiency drop, while a small subset (5 out of 20) showed the opposite tendency. This cancer-type-specific variation of the efficiency decline can be quantitatively explained by a selective modulation of one of the two AE definition steps in cancer. In the same vein as in the *RON* minigene, if the late step becomes slower or the early step becomes faster in cancer compared to its normal counterpart, IR will further accumulate at intermediate PSI. With opposite modulations, IR decreases instead, as observed for some cancers (5 out of 20). The molecular mechanisms of early vs. late AE definition regulation remain elusive, but it is known that RBPs controlling AE definition are commonly mutated in many cancer types, such as SF3B1 [76–81]. SF3B1 regulates the stepwise U2 assembly and 3′SS commitment till the late stages of the spliceosome cycle, and its mutation could strongly disturb the balance between early and late effects. Another possibility is that the relative abundance of different RBPs fails to be maintained in cancer due to loss in homeostasis of gene expression [82, 83], and this disruption therefore causes unbalanced control of distinct splicing steps. In addition, early and late spliceosome states may rely on different energy supplies, where the late stage of spliceosome commitment and remodeling costs more ATPs than the early recruitment stage [84–86]. In cancer cells, energy supply is generally in high demand for many upregulated cellular processes and may be limited for splicing. Therefore, the energy-demanding late step of spliceosome maturation might be more sensitive to the reduced energy supply, resulting preferentially in IR accumulation.

There are certainly open questions for future investigation. For example, even though the PSI-efficiency dependency is a transcriptome-wide trend in many cellular contexts, it is still unclear how widespread this coordinated regulation is at the individual gene or event level. We expect variation between individual exons based on their specific regulatory sequences, which may determine model parameters such as the stepwise AE definition rates. For instance, for the *CD19* minigene, we observed a less pronounced PSI-efficiency dependency compared to *RON* (Supplementary Fig. S2). Likewise, in the transcriptome-wide dataset by Rogalska *et al*. [10], we observed some splicing events showed a coordinated change of PSI and efficiency upon RBP knockdowns, whereas for others the PSI was changed independently of intron retention (Supplementary Fig. S3). We also examined 18 endogenous genes with a three-exon architecture (which matches the minigene construct) in the MAJIQlopedia data and found distinct PSI-efficiency relations (Supplementary Fig. S13). The observed differences between cancer types likely arise from a combination of both gene expression changes and tissue-specific changes in splicing regulator abundance. Furthermore, it will be interesting to see whether splicing efficiency and overall gene expression are coupled, as some groups reported that splicing efficiency increased with increased RNA expression [87], whereas others reported the opposite [88].

Another important question is whether the increase in IR isoform frequency is due to (i) inefficient processing and precursor accumulation, but (mostly) unchanged splicing product concentrations, or (ii) due to enhanced formation of terminal intron retention products and decreased amounts of splicing products. In the *RON* mutagenesis screen, we enriched polyA-containing mRNA before sequencing, which would suggest that we measure readily processed mRNAs, implying that our data favors the latter scenario (ii). For more rigorous testing on a genome-wide level, transient transcriptome profiling (TTseq) may be employed, as with this method, newly synthesized pre-mRNA precursors can be discriminated from the older terminal retention products by pulse labeling [89].

In total, we suggest that the AE and IR decisions are coordinated when the rate-limiting AE recognition steps, i.e. spliceosome assembly and maturation, are modulated. We expect that the stepwise model could be further extended to include additional types of splicing decisions, such as alternative 3′ and 5′ splice-site choice or mutually exclusive AE, so that the coordination of more complex splicing events can be studied in normal cell physiology as well as in search of novel therapeutics for diseases, including cancer.

## Supplementary Material

gkag464_Supplemental_Files

## Data Availability

RNA-seq data were published previously as part of [26] and [31] and can be accessed via ArrayExpress accession numbers E-MTAB-8816 and E-MTAB-6217. Experimental details and primary data analysis were described in [26] and [31]. The scripts of mathematical modeling and data analyses are publicly available via DOI: https://doi.org/10.5281/zenodo.19733073.
