# Supplementary material for: Coordinated alternative splicing decisions via stepwise exon definition
Source: Nucleic Acids Res. 2026 May 19;54(9):gkag464. doi: 10.1093/nar/gkag464 (PMC13183682; doi:10.1093/nar/gkag464)
Supplement: gkag464_Supplemental_Files [file gkag464_supplemental_files.zip › Supplementary_figures.pdf]

Supplementary Figure S1

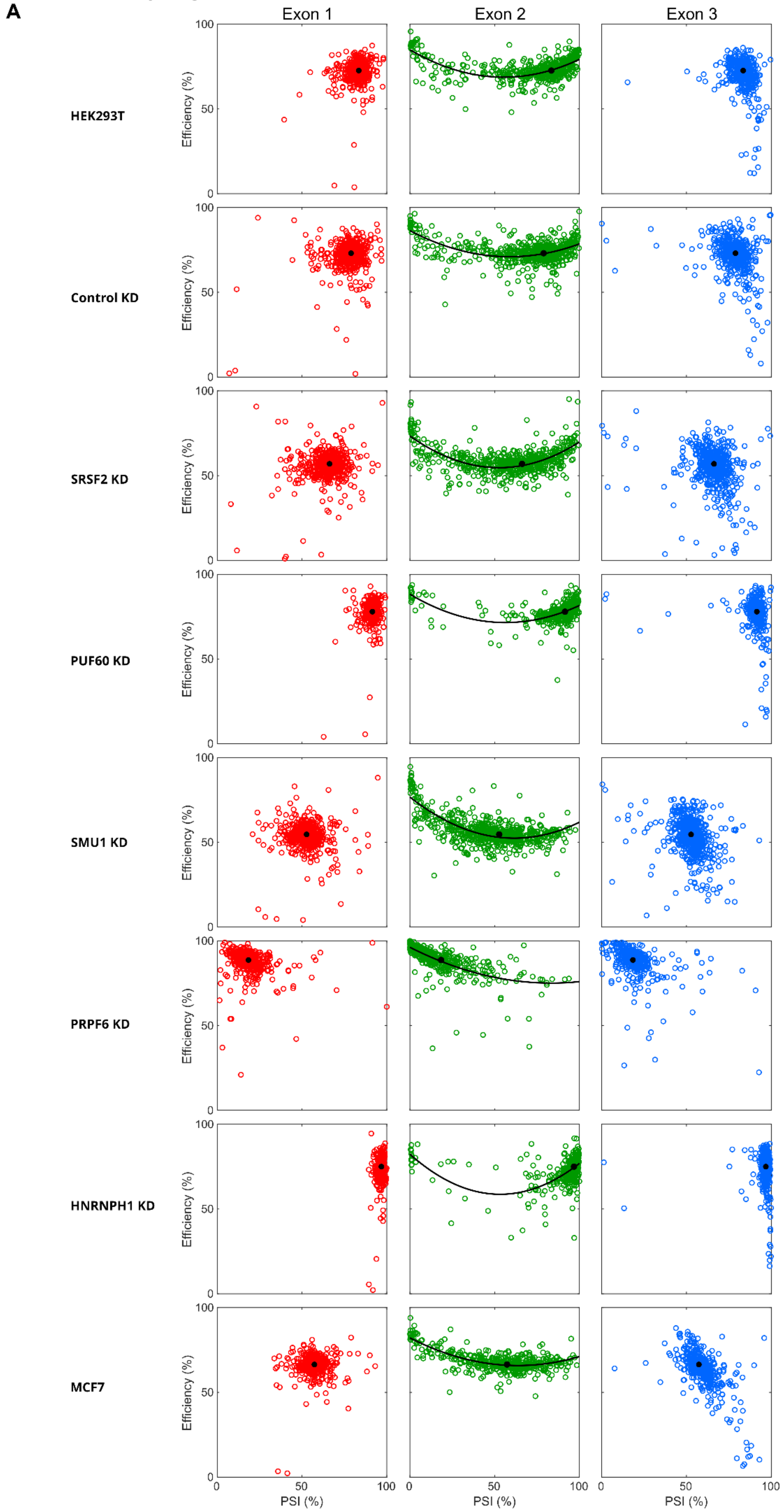

(Supplementary Figure S1 continued)

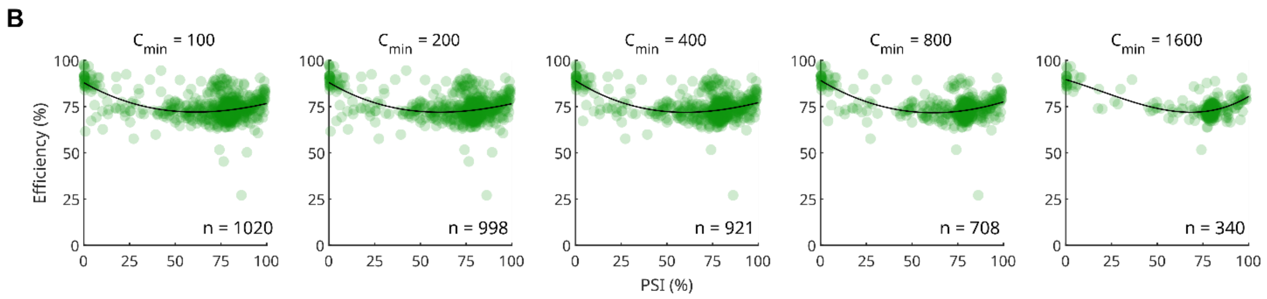

Supplementary Figure S1. PSI-efficiency relationship in the *RON* minigene mutagenesis dataset in HEK293T cells for RBP KDs and non-targeting control

(**A**) PSI-efficiency relation for single point mutants of *RON* minigene in WT, RBP KD and control conditions. Color code represents mutations affecting exon 1 (red, first column), 2 (green, middle column) and 3 (blue, third column), respectively.

(**B**) PSI-efficiency relation is unaffected by read coverage filtering applied to RNA-seq data of *RON* minigenes harboring random mutations. Distinct coverage cutoffs (100, 200, 400, 800 and 1600) were used to keep minigenes with minimal total read count (C_{min}) of the five splice isoforms. Green dots: splicing outcomes of 1020 *RON* minigenes with one or multiple mutations only in exon 2 (not in exon 1 or 3). Black curves: polynomial fit. *n*: number of minigenes after coverage filtering.

Supplementary Figure S2

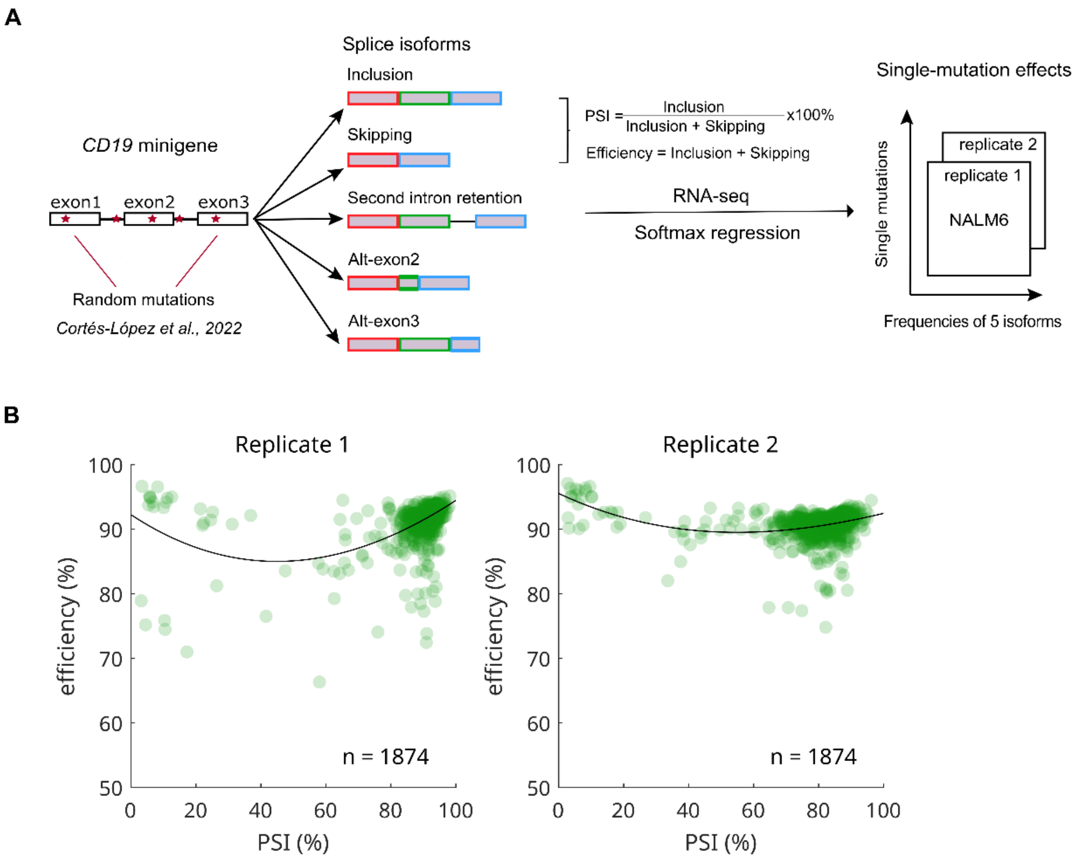

Supplementary Figure S2. PSI-efficiency dependency in *CD19* minigene splicing.

(**A**) High-throughput mutagenesis data of *CD19* minigene. Cortés-López et al. performed regression analysis on the random mutagenesis data of the minigene reporter harboring *CD19* exons 1-3 to quantify the single-mutation effects on abundance of five main splice isoforms, AE inclusion, AE skipping, second intron retention, and two inclusion variants using alternative 3' splice sites in exon 2 and exon 3, respectively. Two metrics were used to characterize splicing outcomes for each mutation: PSI quantifies the binary AE decision, and the splicing efficiency quantifies the summed frequencies of AE inclusion and skipping. The analyzed data from NALM6 cells (with two replicates) is a matrix containing the frequencies of five splice isoforms for 1874 single mutations (including indels) affecting the AE.

(**B**) Splicing efficiency drops at intermediate PSI values in both replicates. Green dots: single mutations within exon 2 or close to the borders of exon 2 (-128 nt upstream of the 3'SS and +148 nt downstream of the 5'SS). Black curves: polynomial fit.

Supplementary Figure S3

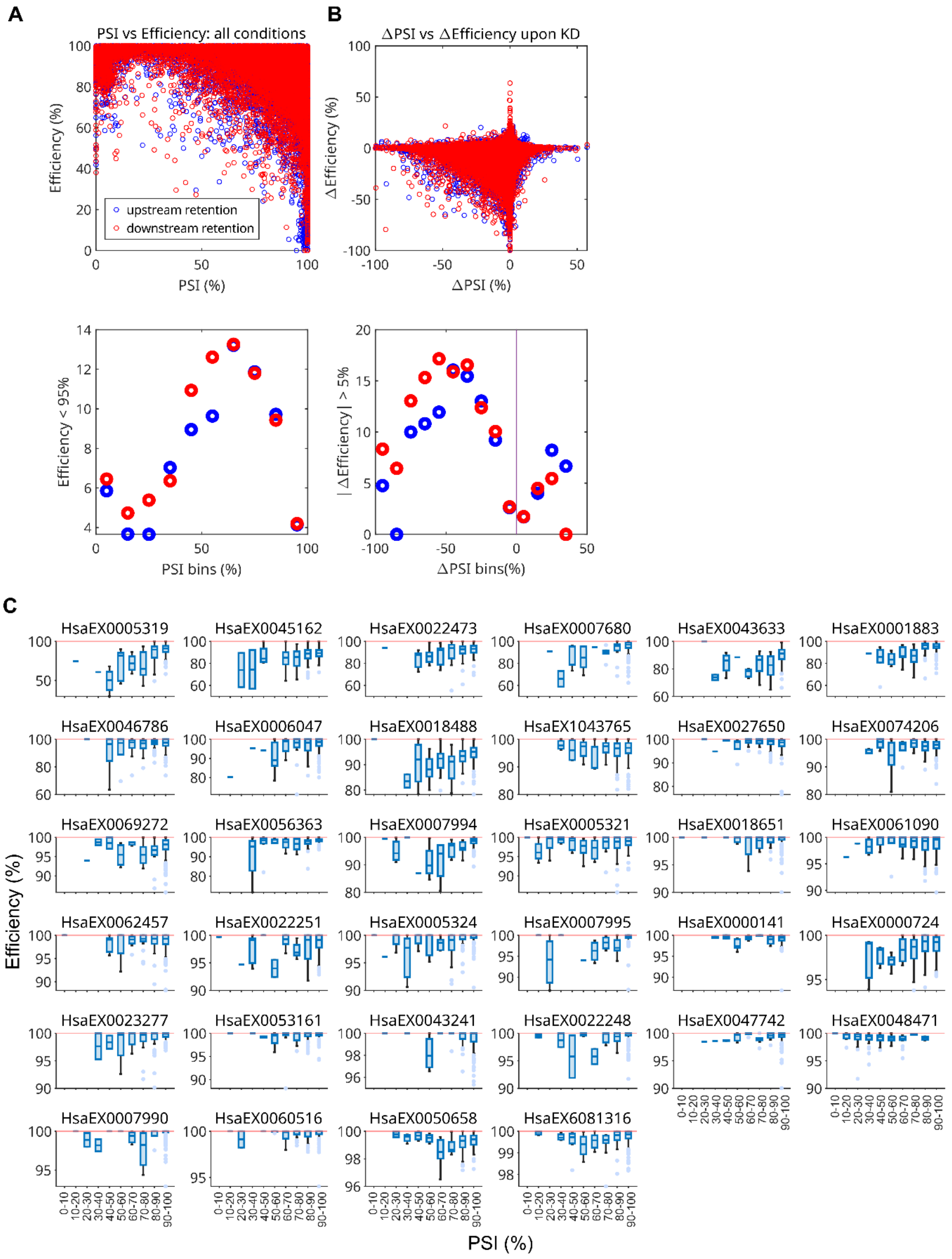

(Supplementary Figure S3 continued)

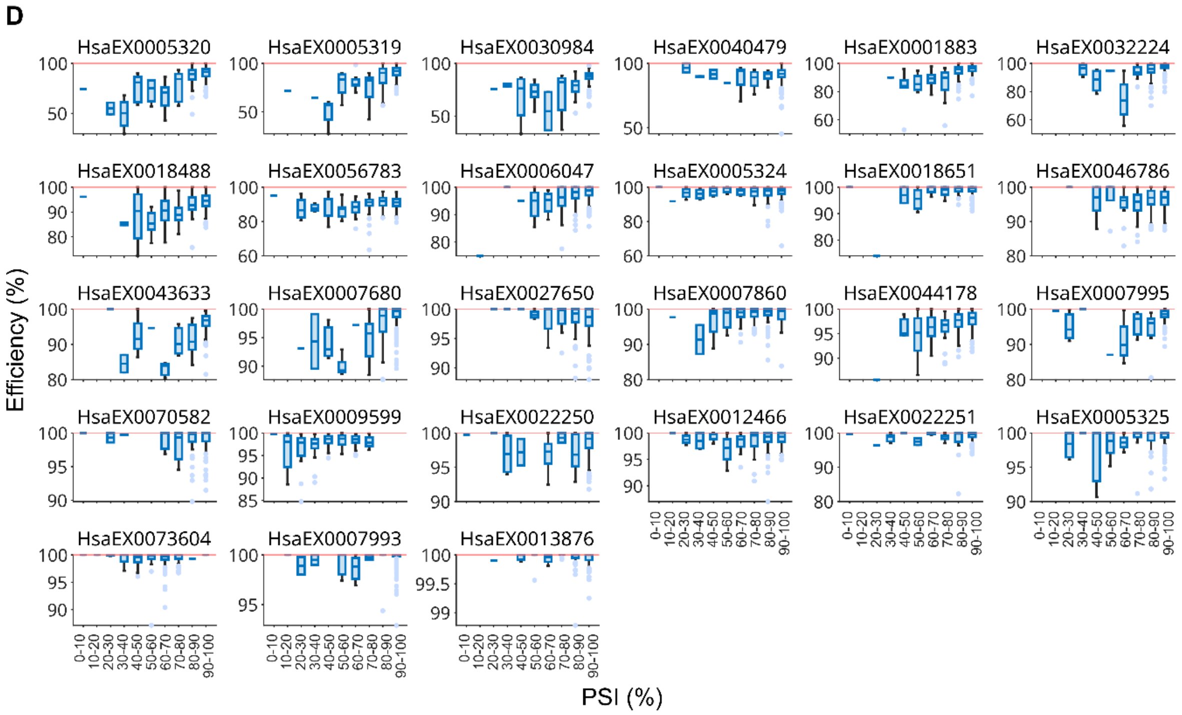

Supplementary Figure S3. Analysis of PSI-efficiency relation in Rogalska dataset (Rogalska et al., 2024) (10).

(**A**) Top: Relationship of PSI and efficiency across all 312 KD and control conditions for 12252 and 12260 exon skipping events with immediately upstream (blue) or downstream (red) intron retention events, respectively. Bottom: Percentage of splicing events with limited efficiency (defined as $100\% \times \left(1 - \frac{\sum \text{intron retention junction reads}}{\sum \text{all junction reads}}\right)$ < 95%) across 10 PSI bins. The data in the top panel was binned according to PSI and the percentage of datapoints with efficiencies < 95% was counted. Most events with limited efficiency are observed for mid PSI values.

(**B**) Top: Relationship of changes in PSI (ΔPSI) and efficiency (ΔEfficiency) across all 312 KD for the events shown in (A top). Bottom: Percentage of events with splicing efficiency changes (|ΔEfficiency| < 5%) across ΔPSI bins ranging from (0-10%) to (90-100%). The data in the top panel was binned according to ΔPSI and the fraction of datapoints with efficiency changes was counted. For ΔPSI ≈ -50%, approximately 15% of events are associated with efficiency changes, and only a small fraction of events with stable PSI show decoupled efficiency changes.

(**C**) and (**D**) Event-specific analysis of PSI-efficiency relation in Rogalska dataset. Each boxplot shows the splicing efficiency as a boxplot across up to 10 PSI bins for the indicated exon skipping events (whose identifiers beginning with 'HsaEX') merging all 312 knockdown and control conditions. Events were considered if they exhibit (i) a PSI standard deviation > 10% across all conditions, ensuring coverage of a sufficiently large PSI range. (ii) non-zero intron retention is measured in more than 100 knockdown conditions to ensure that reliable splicing efficiency estimates. For the 12252 and 12260 exon skipping linked to upstream and downstream intron retention this yielded 34 (**C**) and 27 (**D**) events, respectively, which are sorted with respect to descending standard deviation of splicing efficiency across all conditions (i.e., the first events show strong efficiency variation than the later ones). PSI bins cover a range of 10% (0-10%, 10-20% etc.) and were left empty in the absence of corresponding data. It can be seen that the splicing efficiency towards intermediate PSI values for many but not all splicing events.

Supplementary Figure S4

A

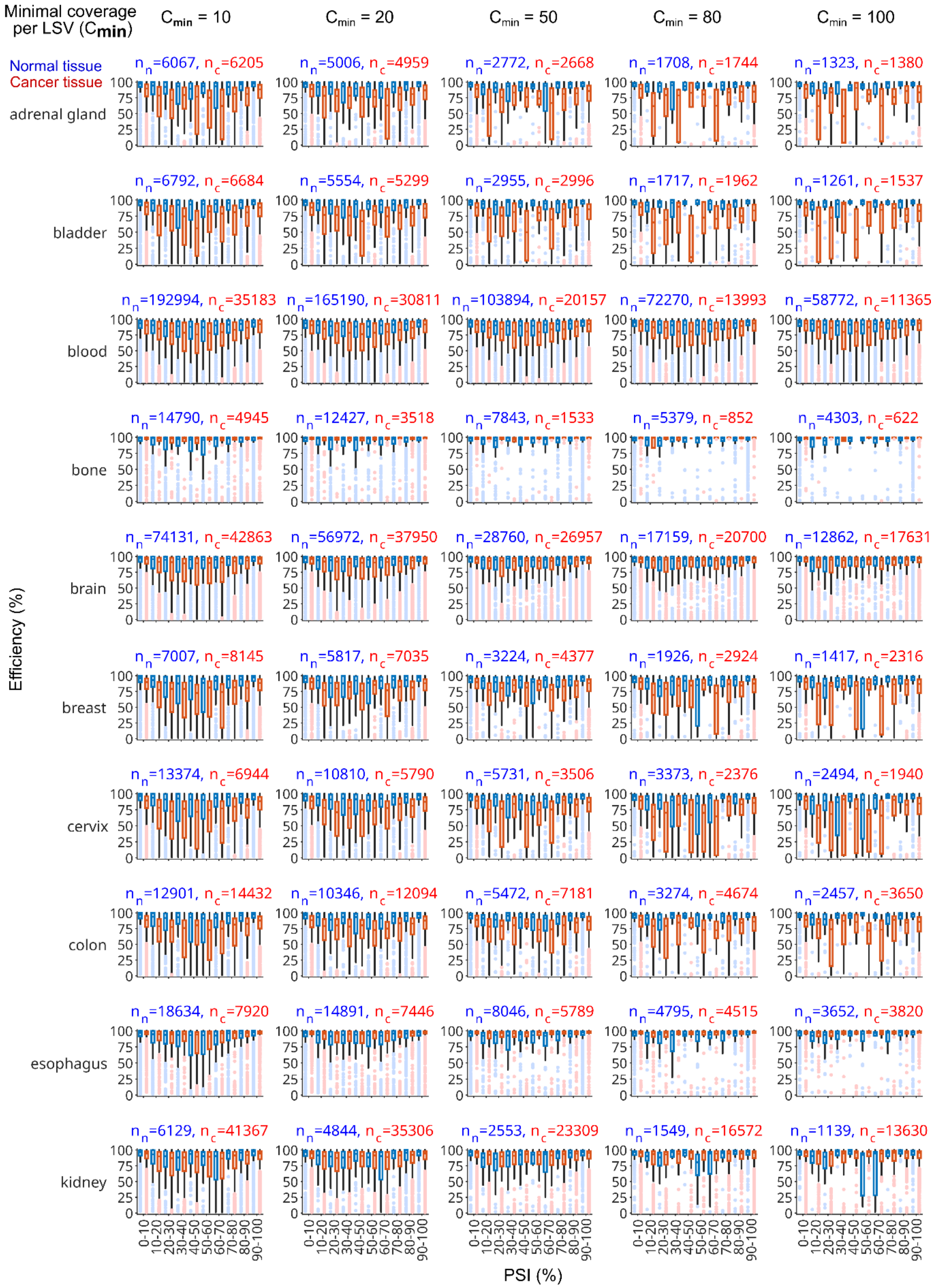

(Supplementary Figure S4 continued)

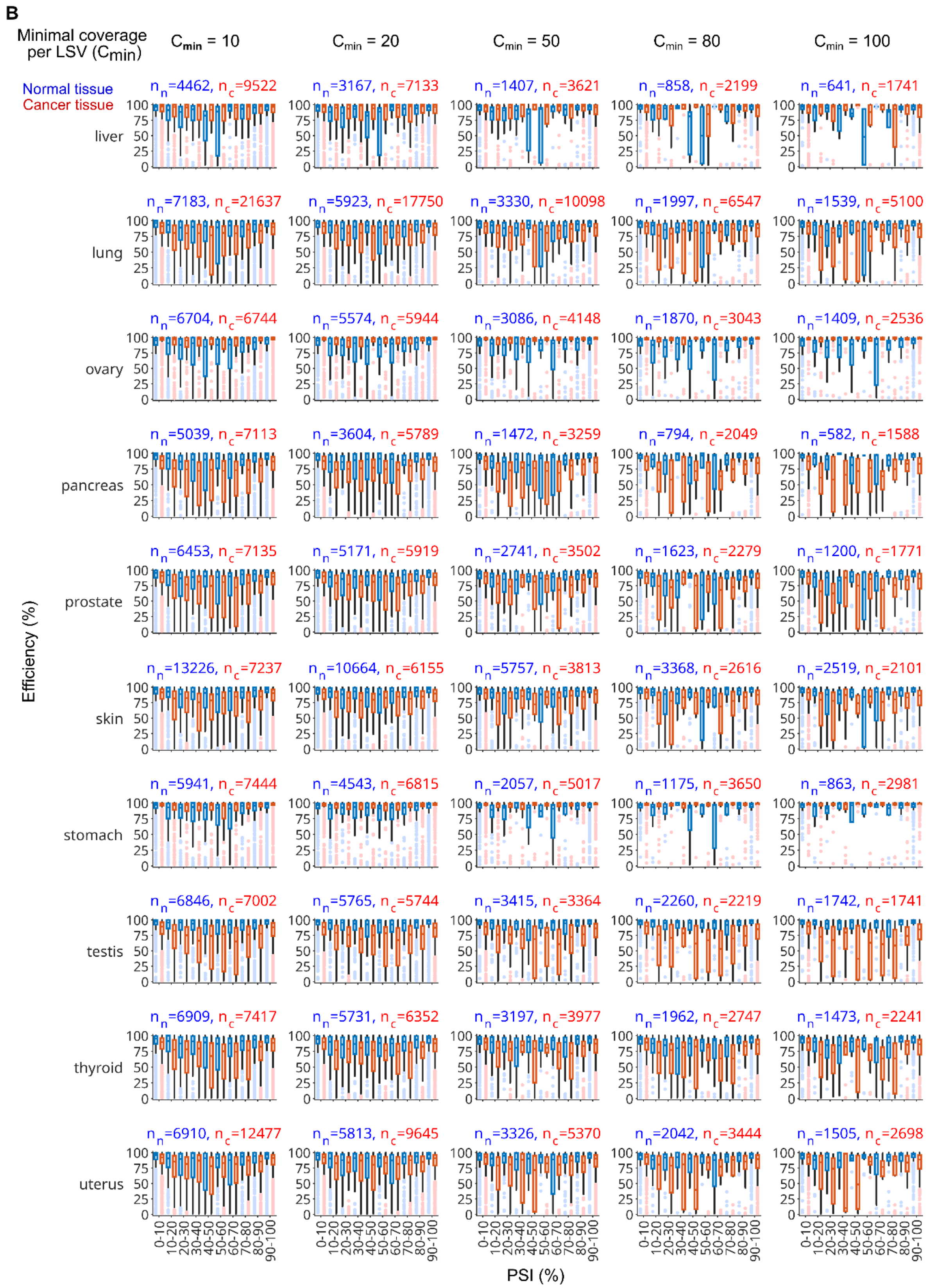

(Supplementary Figure S4 continued)

C

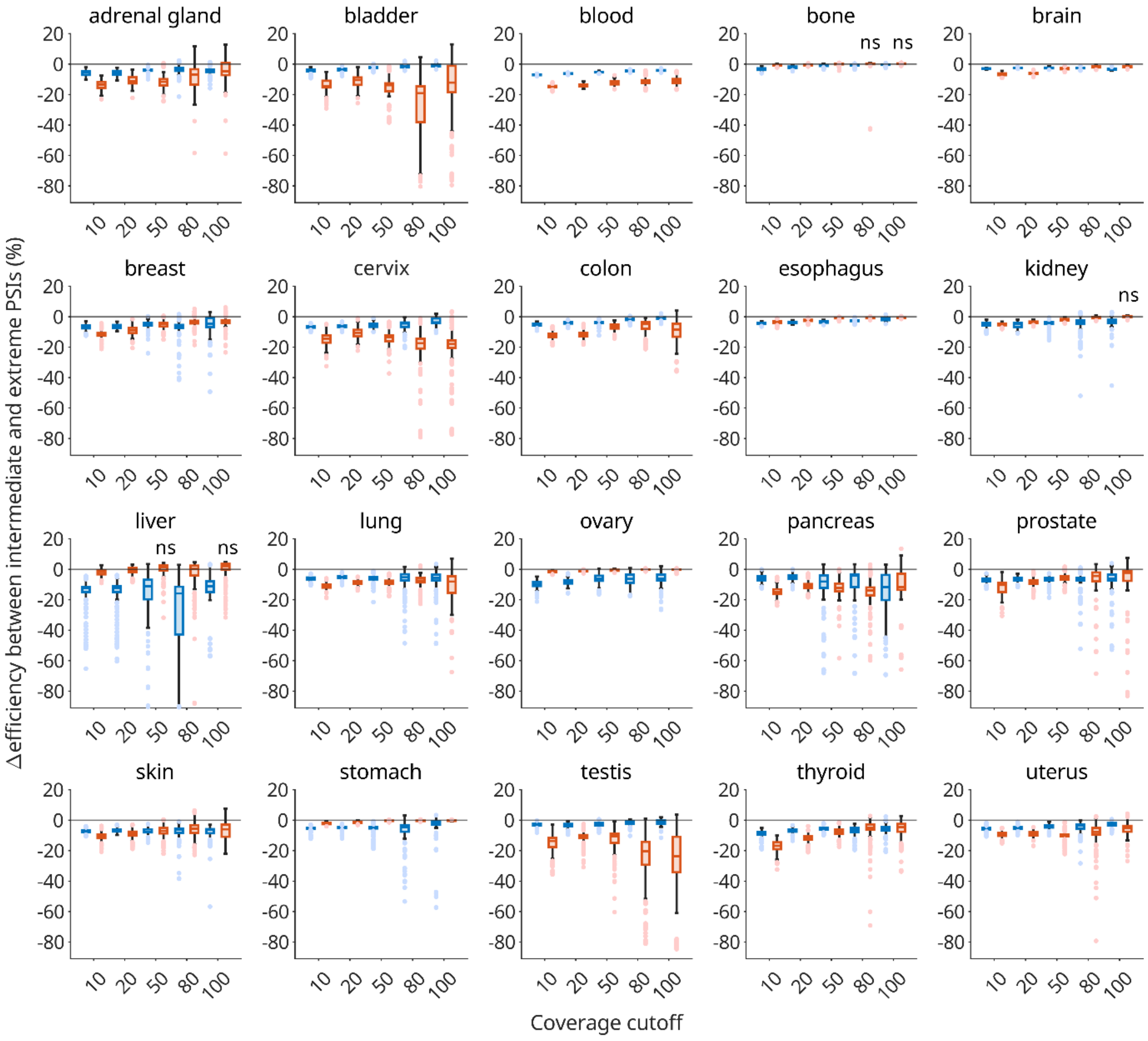

D

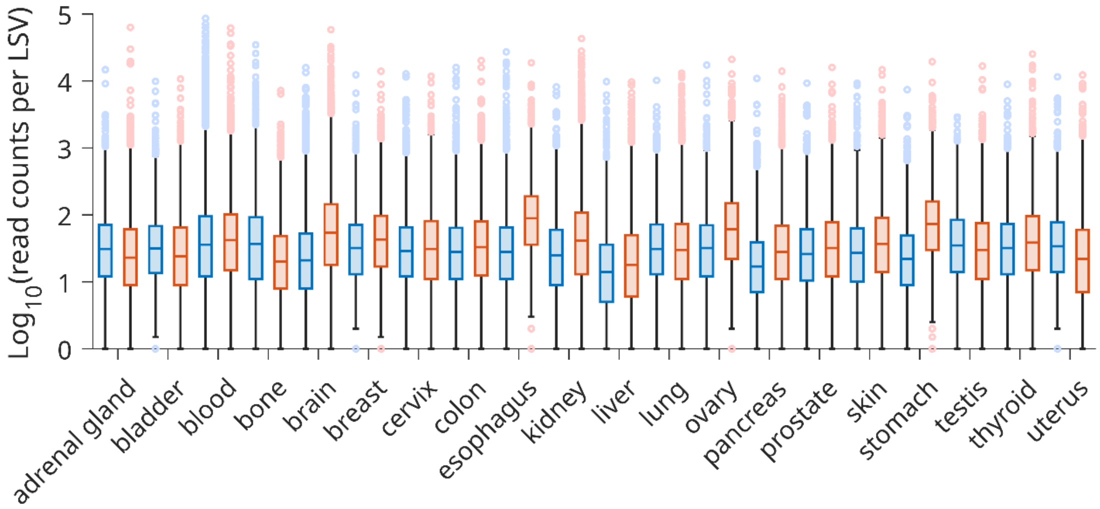

Supplementary Figure S4. PSI-efficiency dependency in normal and cancer tissues in the MAJIQLOPEDIA dataset is robust to read coverage cutoffs.

(**A**) – (**B**) Boxplots showing the distribution of splicing efficiency for uniformly binned PSIs varying from 0-100% for different normal (blue) and malignant tissues (red), tissue origin being indicated on

the left of each row. Different read coverage cutoffs were applied to keep AE-IR LSVs with total junction reads above 10, 20, 50, 80 and 100 (C_{min} for each column). n_n and n_c indicate the total number of splicing events investigated in each normal and cancer tissue, respectively. The lines inside the boxes: median efficiency in each PSI bin. Box: interquartile range; Dots: outliers; whiskers: non-outlier extreme values.

(**C**) Significantly different splicing efficiency between exons with intermediate and extreme PSI values. Bootstrapped difference between median efficiency at intermediate PSIs (40 – 60%) and that at extreme PSIs (combined 0 – 10% and 90 – 100% PSI bins) for distinctly filtered PSI-efficiency data in (A) and (B). 1000 bootstrap samples were generated for each coverage filtered dataset of normal and cancer tissues. One-sided Wilcoxon signed-rank test was performed to test whether the difference in median efficiency is smaller than zero at 95% confidence level. The efficiency decline is significant in all 200 tested datasets except 5 cases (labled with ns).

(**D**) Overall coverage per LSV for the normal (blue) and cancer tissues (red) across all AE-IR events in the MAJIQLOPEDIA dataset.

Supplementary Figure S5

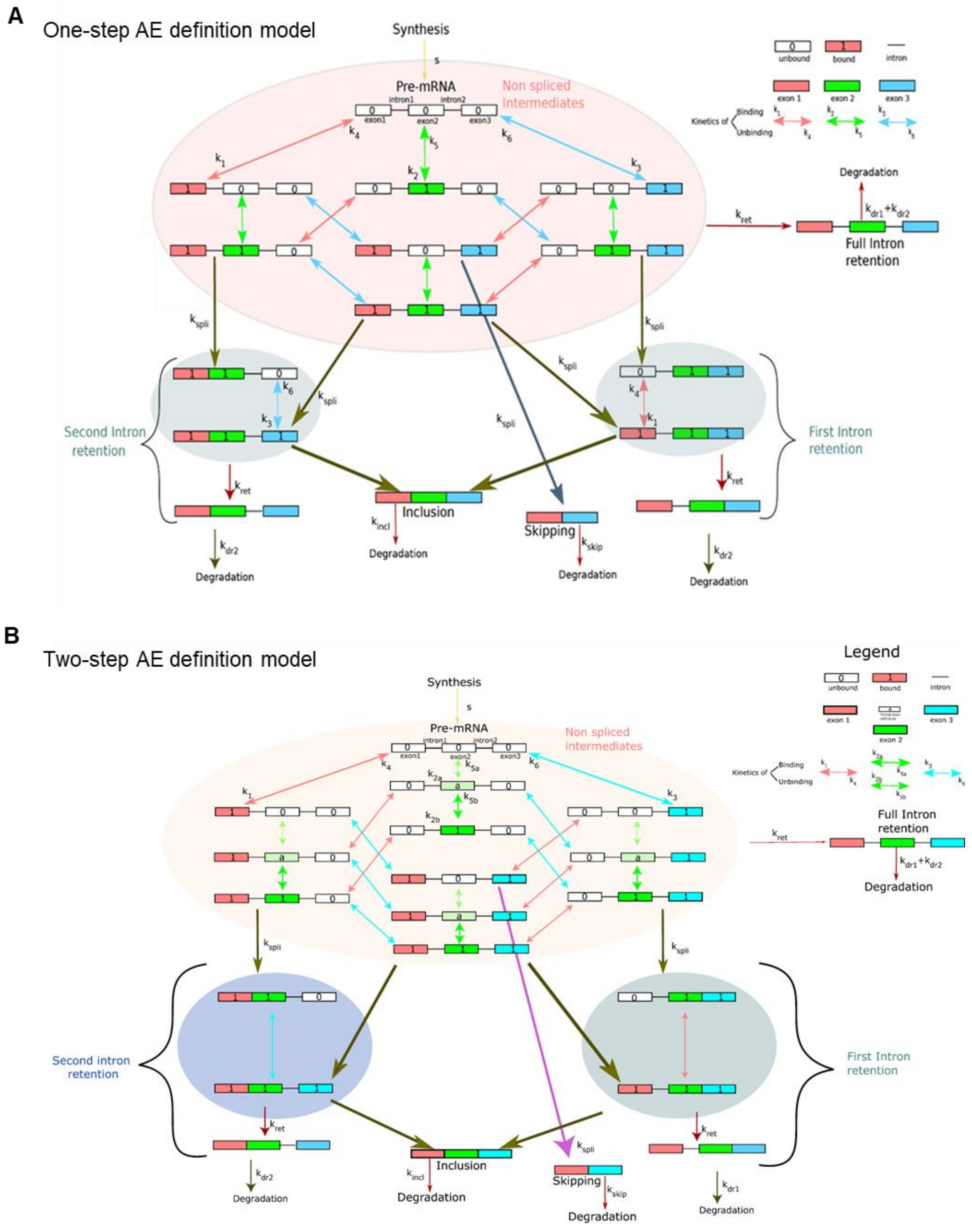

Supplementary Figure S5. Full schemes of the one-step and two-step AE definition models.

(**A**) One-step AE definition model. The spliceosome reversibly assembles across three exons of the newly synthesized pre-mRNA in a combinatorial fashion, yielding8 binding intermediates. The spliceosome binding to exon 1, 2, and 3 were described by a reversible one-step process, with association rates k_1, k_2 and k_3 for exon 1, 2, and 3, respectively, and corresponding dissociation rates k_4, k_5 and k_6. Depending on how these exons are bound, multiple splicing outcomes are possible:

exon 2 inclusion, skipping, first, second and full IR. Splicing occurs when adjacent exons are successfully defined, leading to either intron removal or exon skipping (as shown by the dark green arrows labeled by k_{spli}). The final splicing products are degraded at isoform-specific rates (k_{incl}, k_{skip}, k_{dr1}, and k_{dr2} for AE inclusion, skipping, first and second intron retention, respectively). The degradation rate of the full intron retention isoform is the sum of k_{dr1} and k_{dr2} which reflects that both introns may contain a destabilizing premature stop codon.

(**B**) Two-step AE definition model. Most spliceosome assembly and catalysis steps are described in the same way as in the one-step model, except for the process of AE definition which now requires two consecutive steps while each outer exon still undergoes one-step definition. We used the two rate constants k_{2a} and k_{2b} to quantitatively describe the spliceosome assembly for the first and second AE step, respectively, and assigned k_{5a} and k_{5b} as the rates for the corresponding reverse reactions. There are in total 12 spliceosome binding states in the two-step model. The partially defined AE states (light green AE box) are not committed for active splicing catalysis. Only the fully defined or fully undefined AE can control splicing decisions.

Supplementary Figure S6

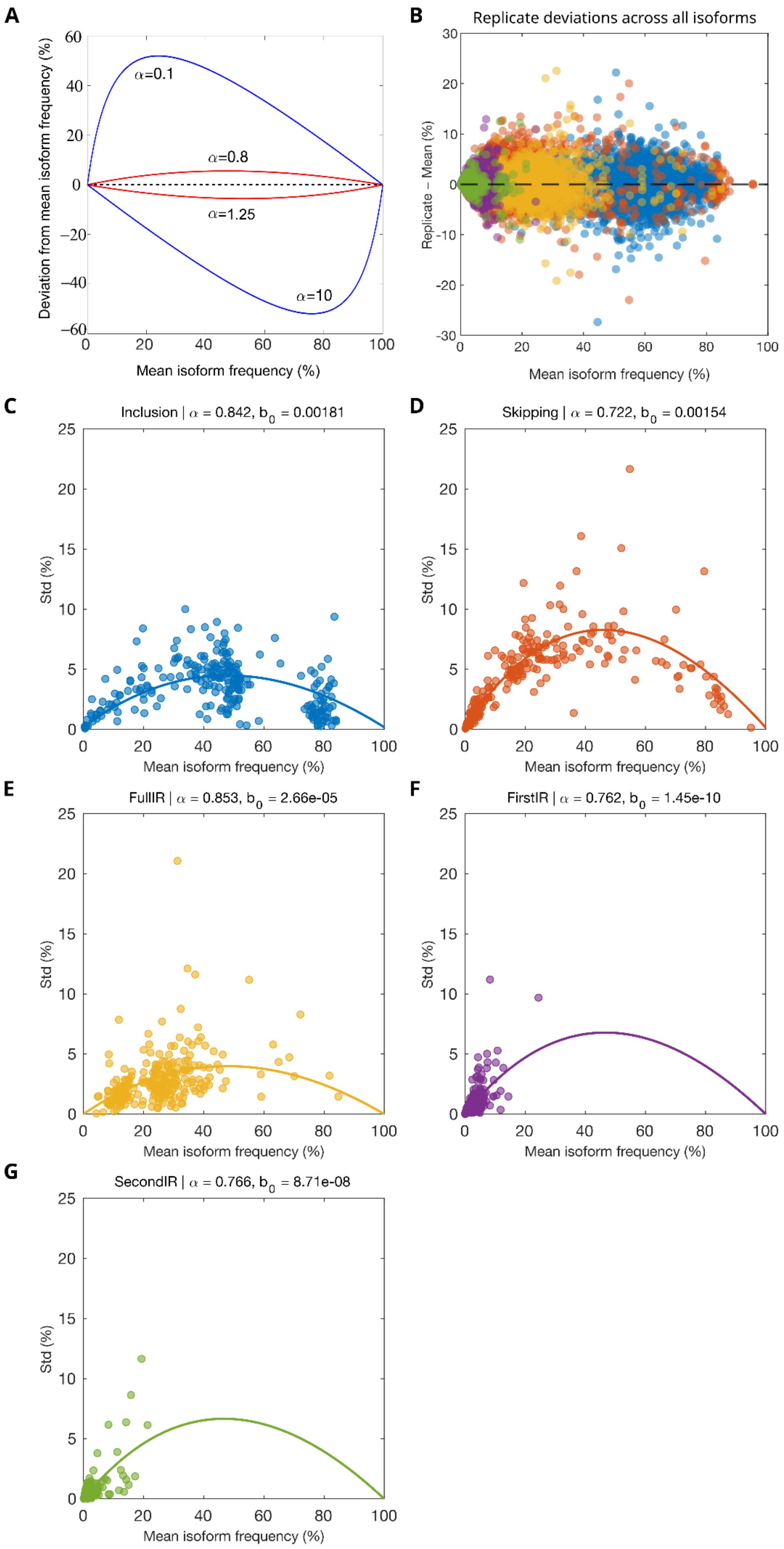

(Supplementary Figure S6 continued)

H

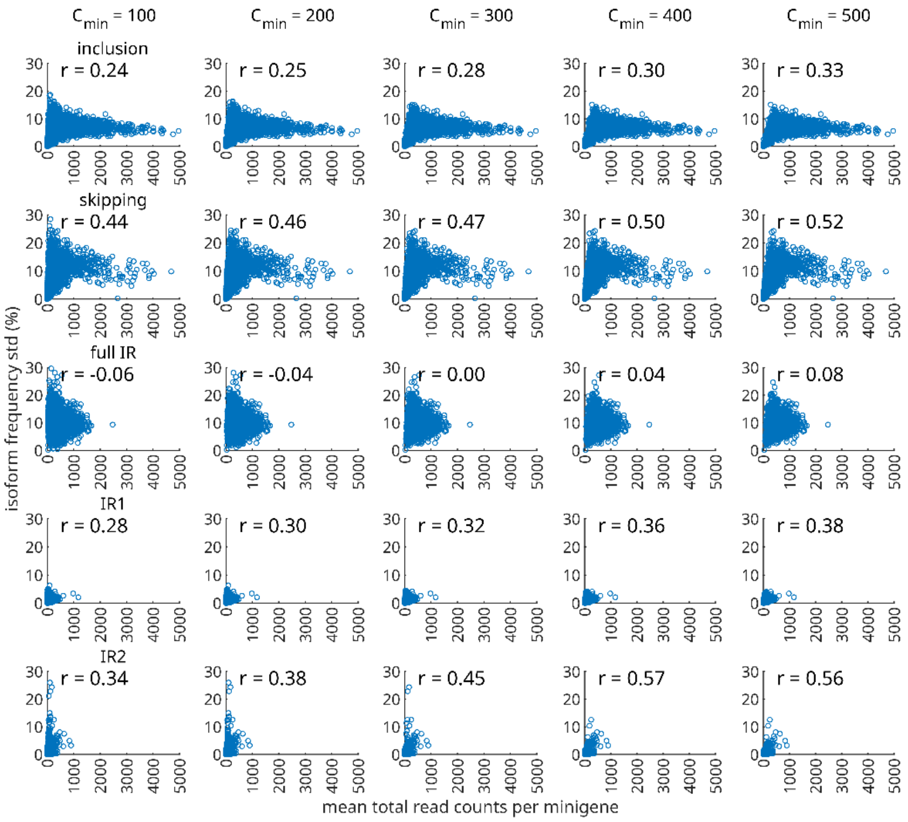

I

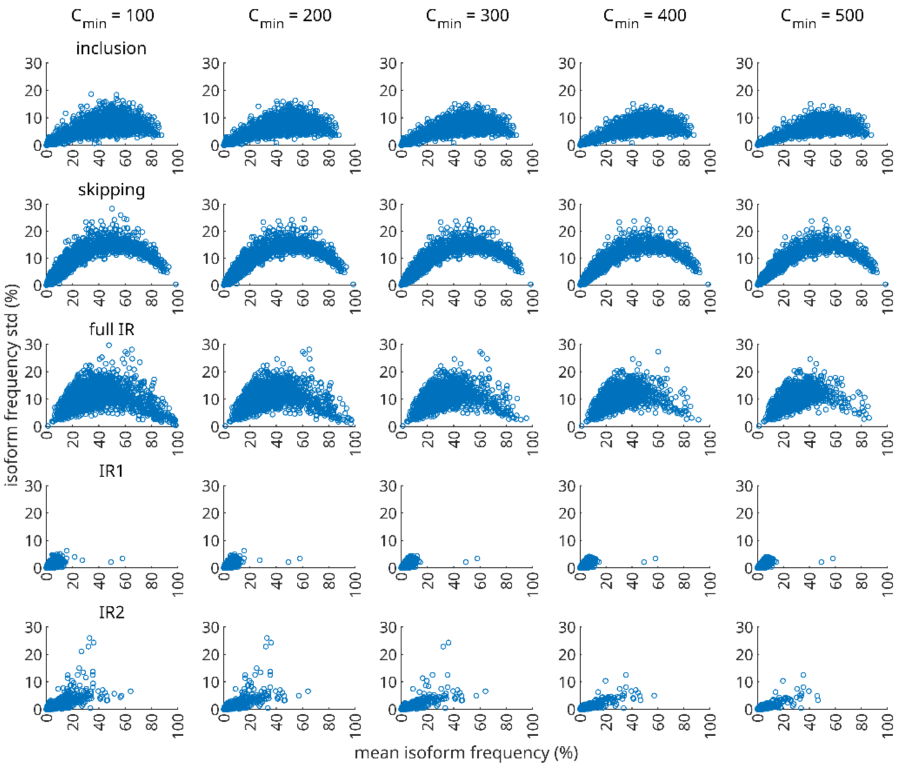

Supplementary Figure S6. Estimation of the nonlinear error model for model fitting and selection.

(**A**) The shape of error distribution from the error model is determined by the amplitude of isoform frequency deviation from the mean value. PSI or isoform frequencies as proportions follow the scaling law (44–46), in which the isoform frequency uncertainty was model using Equation 3, with the key parameter α indicating the error amplitude (Methods). The skewness of the error distribution depends on the amplitude of the uncertainty (or α). The distribution is highly skewed (with long tails) near 0 or 100% isoform frequency when the error is very large (α is much larger or smaller than 1; blue curves), whereas the distribution is mainly symmetric across all frequency values if the error is small (α is close to 1; red curves).

(**B**) Symmetric distribution of *RON* isoform frequency errors, defined as each replicate isoform frequency (n = 3) minus the mean isoform frequency across three replicates of single mutants. Color code: five *RON* isoforms and same as in panels (C)-(G).

(**C**) – (**G**) Isoform-specific error model calibration. Plotted are the mean isoform frequencies of the WT *RON* minigene and 405 effective single mutants (causing larger than 10% change in frequency of at least one isoform) across three biological replicates in HEK293T cells against the corresponding standard deviations. The curves show an error model fit to isoform-specific non-linear noise-mean relationship (Methods). The color code reflects the five different splice isoforms. The best-fit isoform-specific error model parameter values (α and b_0) were reported in the title of each subpanel.

(**H**) Isoform frequency error is insensitive to isoform sequencing read counts. *r*: Pearson correlation coefficient between the standard deviation of isoform frequency and the mean isoform sequencing read counts. Columns: distinct coverage cutoffs (100, 200, 300, 400 and 500) applied to filter the RNA-seq data. One point represents one minigene with combined mutations.

(**I**) The error-mean relation for each isoform is insensitive to distinct coverage cutoffs (100, 200, 300, 400 and 500, same as in C – G).

Supplementary Figure S7

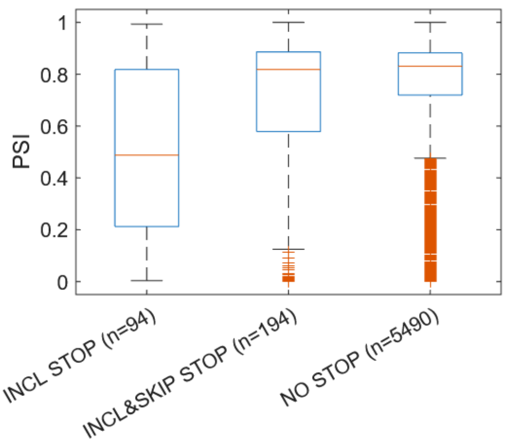

Supplementary Figure S7. PSI distribution of *RON* minigene populations harboring an in-frame stop codon in the inclusion isoform but not in skipping (left), in inclusion and skipping (middle) or in neither of the two isoforms (right). Even though a stop codon in the inclusion isoform is associated with a low PSI, nonsense-mediated decay (NMD) is unlikely to occur in *RON*, since the minigene lacks the ORF configuration required for NMD. Furthermore, the PSI shift in the INCL STOP population is downregulated by two splicing-effective point mutations (see Methods).

Supplementary Figure S8

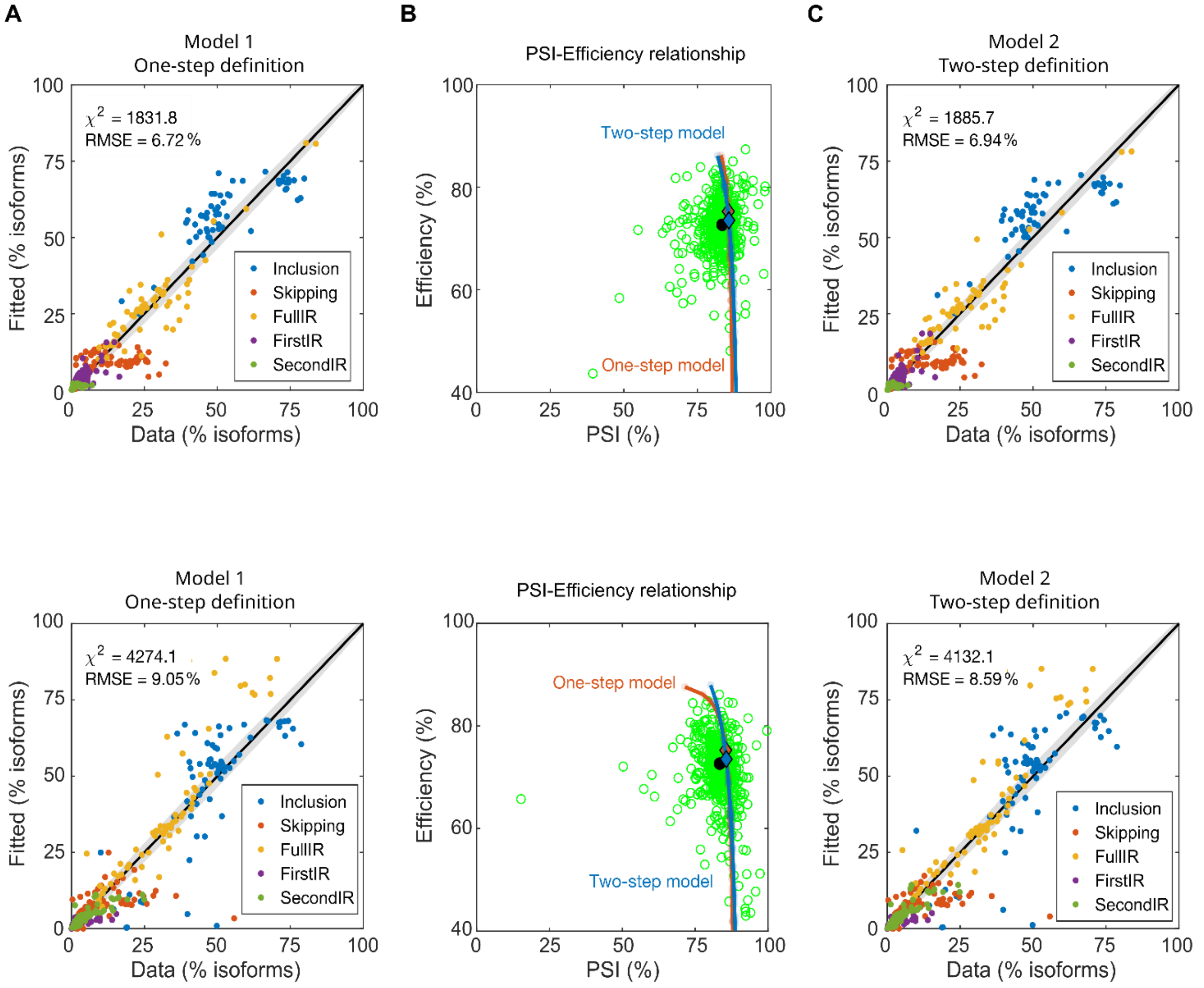

Supplementary Figure S8. Model fitting for mutations affecting outer consititutive exons of *RON* minigene.

(**A**) One-step AE definition model captures *RON* splicing modulated by the single mutations in the outer exons. The one-step model was fitted to the frequencies of all five *RON* isoforms for 405 effective single mutations in exons 1-3. Shown here is the fit to 62 mutations affecting exon 1 (top) and 6 mutations affecting exon 3 (bottom). Black lines: the diagonal line representing perfect data-model agreement; shade: isoform variability predicted by an error model estimated from the data (Methods). Corresponding fits to exon 2 mutations can be found in Fig. 2B.

(**B**) Independent AE-IR modulation by outer exon mutations were described by the both the one-step and two-step models. The data in green circles represent the mutations in the exon 1 (top) and exon 3 (bottom) of the *RON* minigene and the lines are simulations using the best fits of the one-step (red) and two-step (blue) model, respectively. Black dot: WT data; red and blue diamonds: fitted WT by one-step and two-step model, respectively.

(**C**) Two-step AE definition model captures *RON* splicing modulated by the single mutations in the outer exons. Same representation as in (**A**). Corresponding fits to exon 2 mutations can be found in Fig. 2D.

Supplementary Figure S9

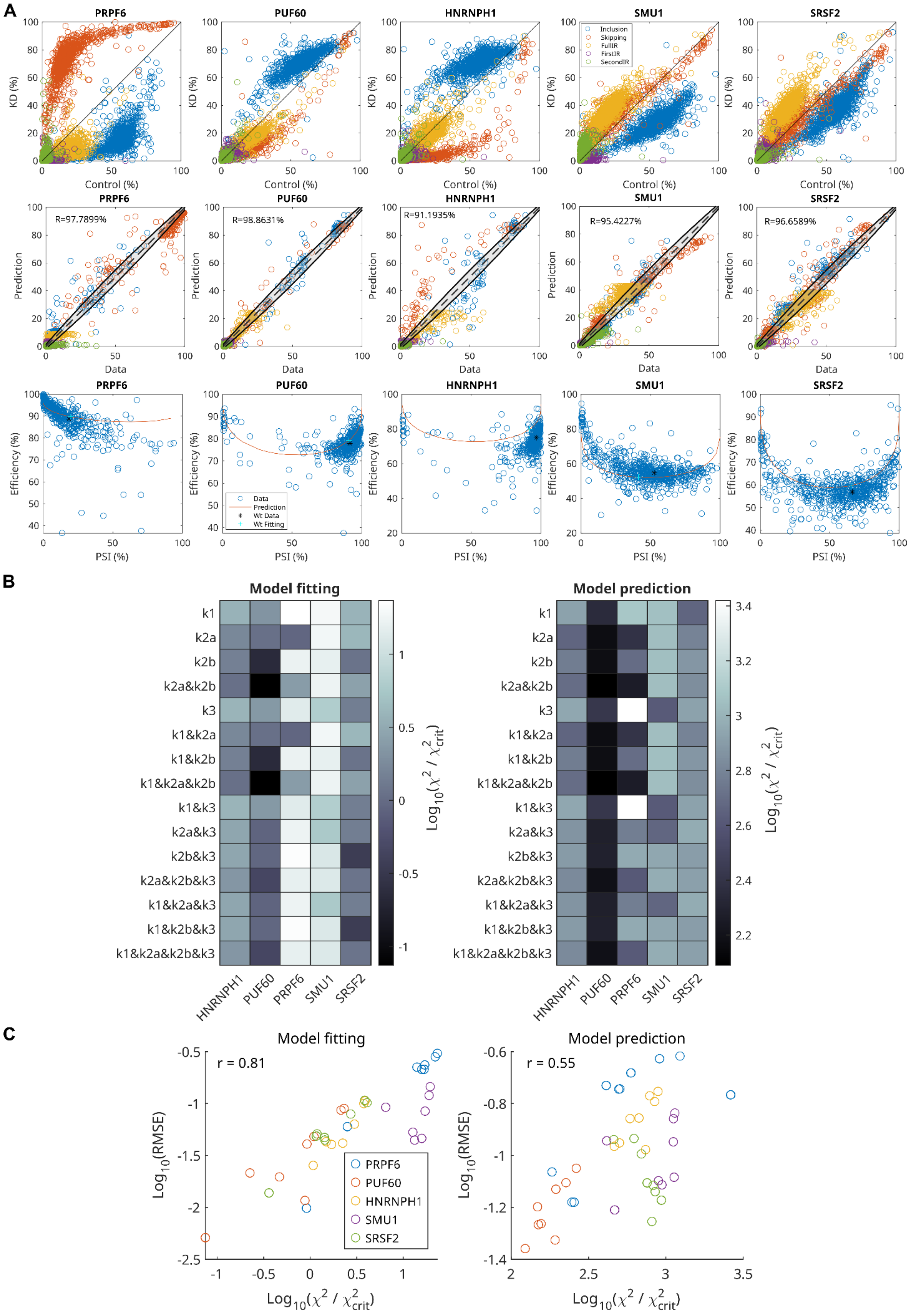

Supplementary Figure S9. Quantifying RBP KD effects on *RON* minigene splicing

(**A**) Systematically selected best models capture the RBP KD effects on isoform frequencies and coordinated AE-IR regulation.

Top row: Measured changes in isoform frequencies of KD scrambled control vs. the five RBP KDs for the WT *RON* minigene and 405 single mutants. Same representation as in Fig. 5A.

Middle row: Predicted *RON* single point mutant isoform frequencies for each KD generated based on the model variant that best fits the unmutated *RON* minigene (see Fig. 5B) are compared to the corresponding data. The results show strong linear correlation between predicted and measured values, indicating the selected best models faithfully capture the data (R: Pearson correlation). Same representation as in Fig. 5C.

Bottom row: the relationship between PSI and efficiency for the five RBPs. The blue circles show the measured PSI-efficiency for all AE mutations and the WT minigene (black dot), each RBP KD being represented in one column, alongside with a simulation of the best-fitting model variant (red line). All predictions accurately capture the observed drop in efficiency at intermediate PSI levels. Same representation as in Fig. 5D.

(**B**) Quantifying model selection on regulation modes of distinct RBPs using χ^2 metric. Model fitting (left) and prediction (right) results from the 15 model variants were characterized by the by the χ^2 value relative to the critical χ^2 value defined by the degree of freedom of each model variant at 95% confidence level. The model variants are indicated by the RBP-regulated parameter combinations on the left, which are given in the same order as in Fig. 5B (k_1: rate of exon 1 definition; k_{2a} and k_{2b}: rates of first- and second-step exon 2 (AE) definition, respectively; k_3: rate of exon 3 definition). Color code: $\text{Log}_{10}\chi^2$ ratio.

(**C**) Systematic model discrimination using χ^2 and RMSE metrics yields consistent conclusions. χ^2 and RMSE metrics quantifying the model fitting (left) and prediction (right) are highly correlated. The same/similar models are selected using both metrics, especially for RBP KDs yielding a very good fit (all except SMU1). *r*: Pearson correlation coefficient. Color code: RBP KDs.

Supplementary Fig. S10

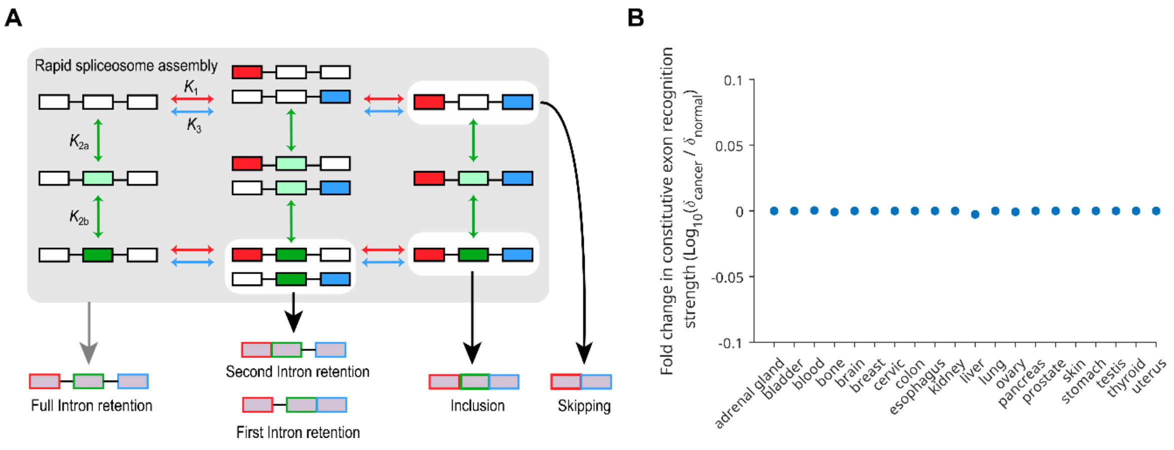

Supplementary Figure S10. Inference of transcriptome-wide PSI-efficiency relation in normal and cancerous tissues using reduced core model of two-step AE definition.

(**A**) A core two-step AE definition model simplified from the full model in Fig. 2 and Supplementary Fig. S5. This core model assumes that spliceosome assembly at each exon rapidly reaches equilibrium, governed by the equilibrium constants K_1 (for exon 1), K_3 (exon 3), K_{2a} and K_{2b} (for two steps in AE definition, respectively). Spliceosome binding states determine splicing decisions, and 4 out of the 12 binding states (in white boxes) catalyze active splicing reactions, whereas the remaining 8 spliceosome binding states (in grey shade) are not catalytically active and give rise to full intron retention isoforms. The fully defined state in which all exons are bound gives rise to the AE inclusion isoform. The AE will be skipped when the AE definition is completely absent but well established at the outer exons. A fully recognized AE paired with only one defined outer exon produces the partial intron retention isoforms.

(**B**) The core model was fitted to PSI-binned efficiency data of 20 tissues in normal and malignant conditions. The recognition strength of the outer (constitutive) exons is barely affected across all 20 cancer types. Details of the core model derivation and parameterization can be found in the Methods.

Supplementary Figure S11

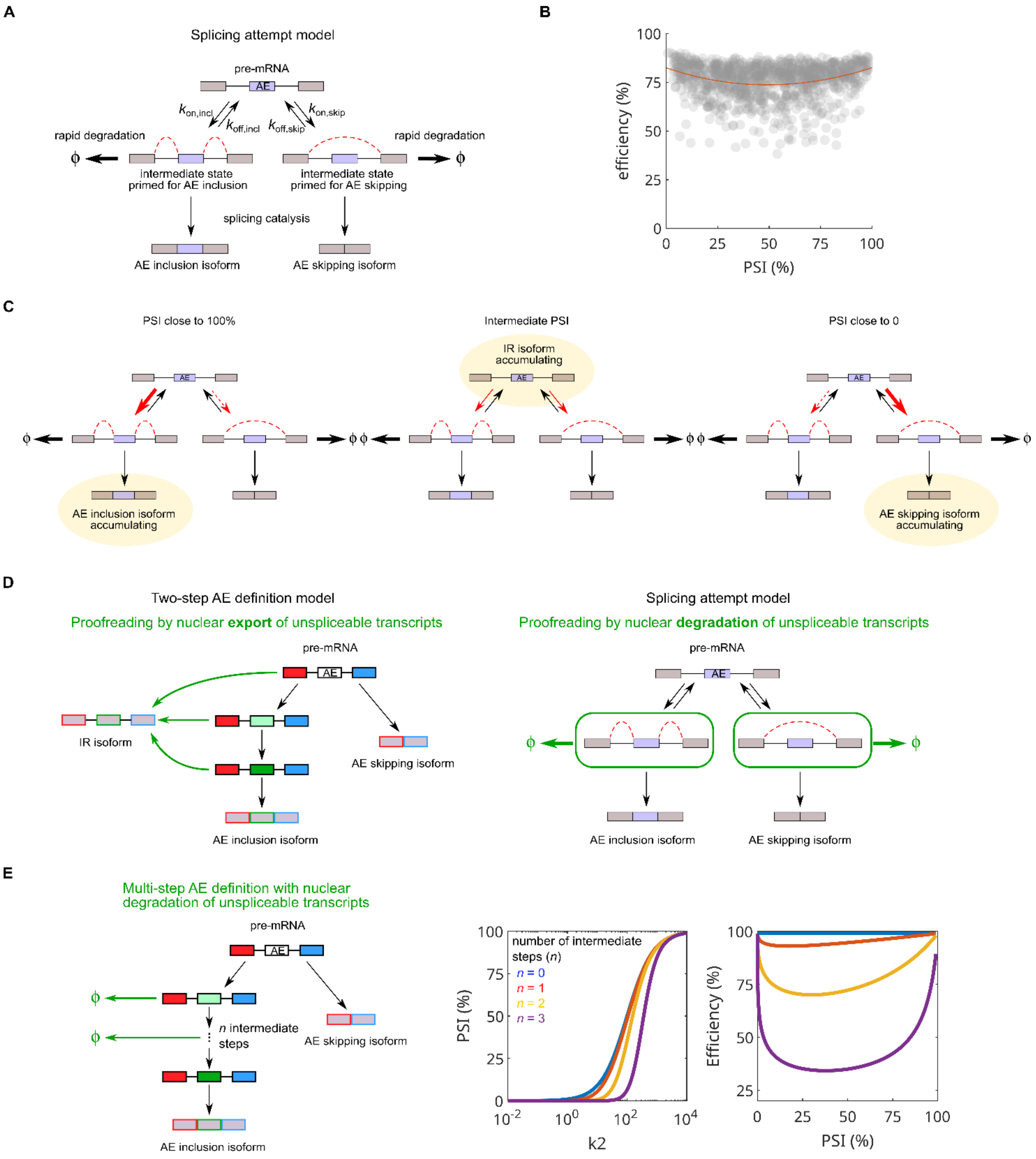

Supplementary Figure S11. PSI-efficiency relation in a model with fast turnover of transcripts with failed splicing attempts.

(**A**) Schematic representation of the splicing attempt model. The newly synthesized transcript can reversibly transition to two splicing intermediate states primed for either AE inclusion or skipping, both of which are subjected to rapid degradation or mediate the production of AE inclusion and skipping isoforms via irreversible splicing catalysis. $k_{on,incl}$ and $k_{on,skip}$ are the transition rates from native pre-mRNA to AE inclusion and skipping primed, respectively, while are the $k_{off,incl}$ and $k_{off,skip}$ rates of corresponding opposite reactions.

(**B**) Quantitative model simulation. To mimic the modulations on AE recognition across all exons in the human genome, the rates of splicing priming ($k_{on,incl}$ and $k_{on,skip}$) were randomly sampled following lognormal distribution (1000 samples), while the other parameters kept constant. Grey dots: PSI and efficiency values calculated for each sample. Black curve: polymomial fit.

(**C**) Qualitative analysis and interpretation of the splicing attempt model based on the simulation in (B). Left and right panels: an AE that is strongly included or skipped (imbalanced $k_{on,incl}$ and $k_{on,skip}$ values) has low frequency of splicing attempts or trials, as once arrived at the intermediate state the transcript will be immediately spliced before being degraded. Middle panel: a balanced choice of both inclusion and skipping decisions (similar $k_{on,incl}$ and $k_{on,skip}$ values) leads to repeated toggling between inclusion and skipping primed states, which delays the splicing catalysis, leading to an accumulation of unspliced transcripts (IR).

(**D**) Conceptual comparison of the two-step AE definition model proposed in Fig. 3B with the splicing attempt model (panel A). Both models share the same feature of kinetic proofreading on the AE decision, while they differ in details of implementing the mechanism. Proofreading is achieved via unclear export or degradation of the unspliceable transcripts in the two-step AE definition and the splicing attempt model, respectively (arrows highlighted in green).

(**E**) Implementating nuclear degradation of spliceosome assembly intermediates into multistep AE definition model generated little difference in describing the efficiency drop at intermediate PSIs. A model variant of multi-step AE definition was developed, in which the intermediate spliceosome assembly states are subjected to degradation instead of nuclear export (left panel). Splicing regulation by modulating the AE recognition strength (k_2) was simulated using this model variant with n intermediate states, n = 1, 2, 3 and 4 (color coded). The switch-like behavior of AE inclusion/skipping (middle panel) and the efficiency drop at intermediate PSIs (right panel) were recapitulated, in a manner qualitatively consistent with the original model in Fig. 3.

Supplementary Figure S12

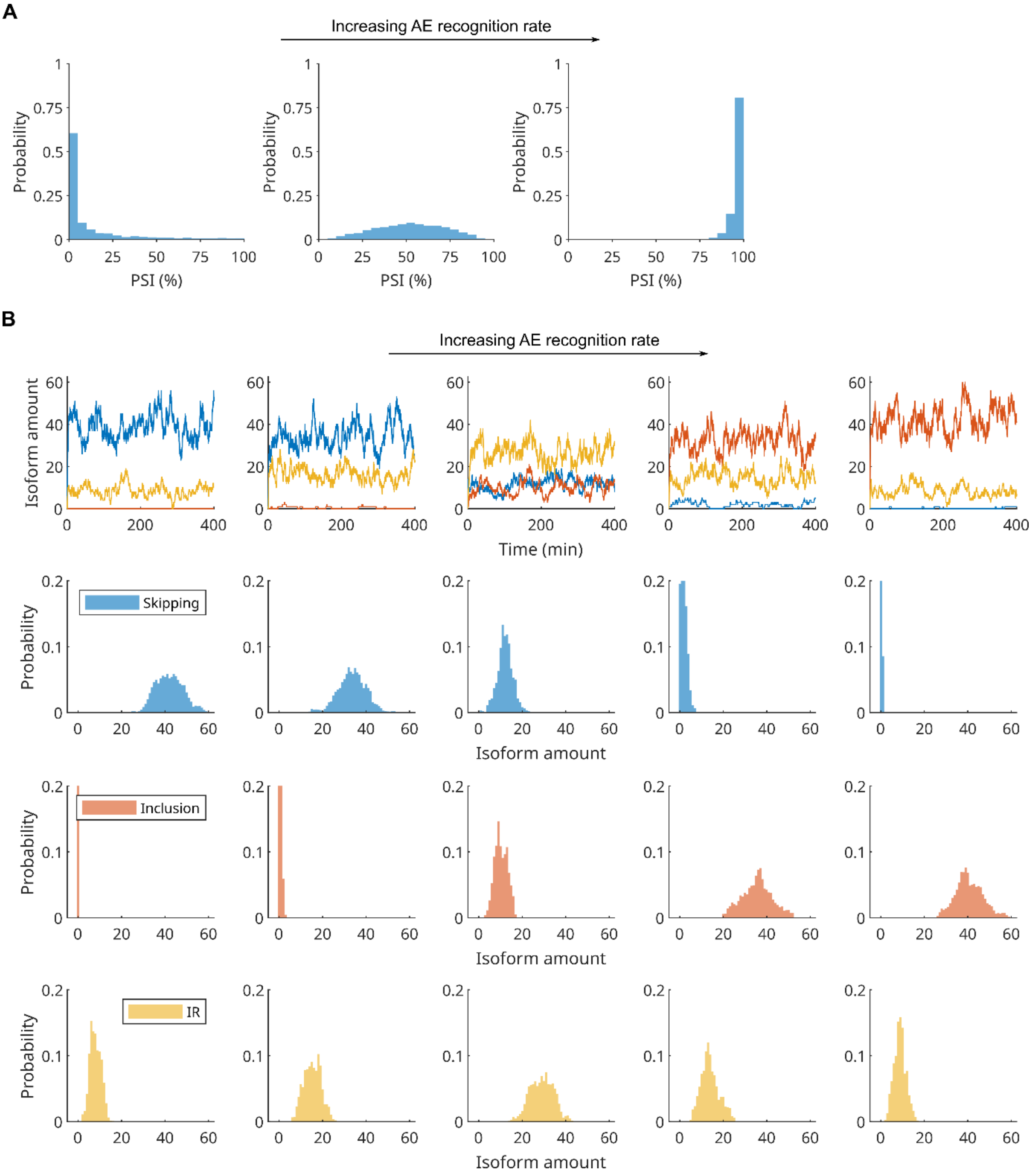

Supplementary Figure S12. Simulation of the splicing switch between AE inclusion and skipping in single cells.

(**A**) PSI gradually increases with AE recognition strength in individual simulated cells in a deterministic simulation with random parameter sampling. The two-step AE definition model allows for a splicing switch between AE inclusion and skipping isoforms, which is quantified by a sigmodal curve of PSI as a function of the AE definition rate (Fig. 3A, red curve). This sigmodal curve can be approximated using a Hill function $PSI = k_2^h/(K^h + k_2^h)$, where the half-saturation point is K = 0.3 and the Hill coefficient h = 1.6 (values obtainted from Fig. 3A). The parameter k_2 characterizes the proportional change in the first- and second-step AE definition rates (same as in Fig. 3A). To mimic the PSI switch in single cells, k_2 was randomly sampled following a log-normal distribution with different mean values (0.03, 0.3 and 3; n = 2000 per mean value; standard deivation = 2). The

resulting PSI showed unimodal distributions across different k_2 means, suggesting that AE decision is collectively regulated in all individual cells, rather than only in a subpopulation.

(**B**) Gradual regulation of AE inclusion, skipping and IR isoforms in stochastic simulations of single cells. Stochastic simulation of the two-step toy model (Fig. 3B, middle scheme) was performed using Gillespie algorithm at five different AE recognition strenghs k_2 = 0.03, 0.3, 3, 30 and 300 (columns). The reaction propensities of first- and second-step AE definition are k_2 and αk_2, respectively, where α = 1/30 (constant across all k_2 values). The other model parameters were the following: splicing catalysis rate k_{spli} = 1; rate of intron retention via nuclear export k_{ret} = 0.2; pre-mRNA synthesis rate vsyn = 5; isoform degradation rate k_{deg} = 0.1. First row: simulated temporal dynamics of AE inclusion (red), skipping (blue) and IR (yellow) isoforms in a single cell. Second to last rows: distributions of corresponding isoform abundance from the time courses in the first row. All distributions are unimodal, indicating a gradual shift from dominant skipping (left column) to inclusion (right column).

Supplementary Figure S13

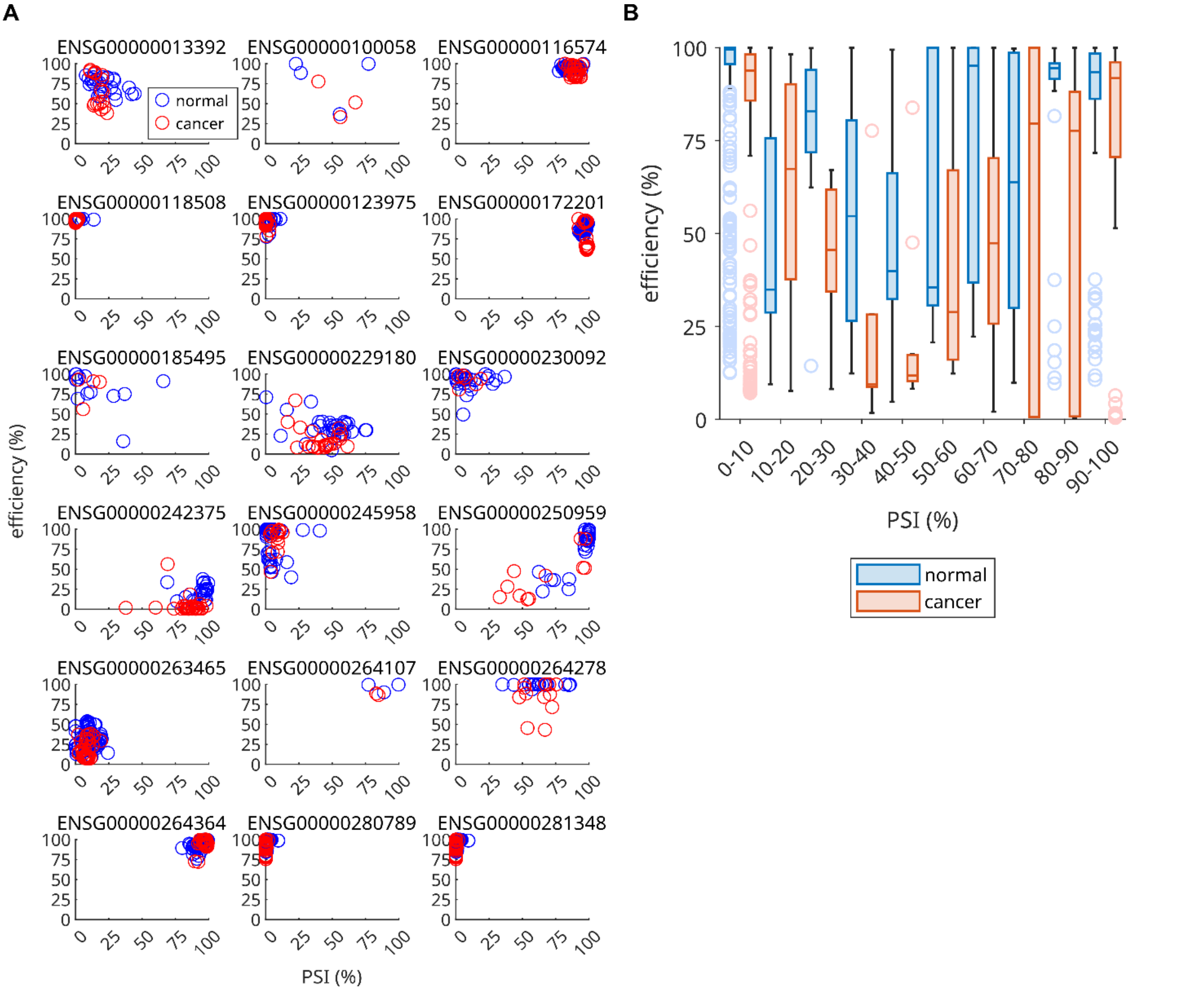

Supplementary Figure S13. Splicing efficiency drop at intermediate PSIs for three-exon genes in the MAJIQLOPEDIA datasets.

(**A**) 18 three-exon genes with the indicated Ensembl gene IDs were found in both normal and cancer tissue datasets. Their PSI and efficiency were plotted across 20 tissues in healthy (blue dots) and malignant (red dots) conditions.

(**B**) PSI-efficiency box plot after pooling all data points of the 18 genes in normal (blue) or cancer tissues (red).
